# Plastron-mediated direct H_2_O_2_ synthesis

**DOI:** 10.1038/s41467-026-76198-9

**Published:** 2026-08-04

**Authors:** Kang Wang, Vivekananda Sinha, Anthony J. Hayes, Alexandre Boucher, Alberto Roldan, David J. Morgan, Richard J. Lewis, Graham J. Hutchings, Marc Pera-Titus

**Affiliations:** 1 Cardiff Catalysis Institute, School of Chemistry, Cardiff University; Translational Research Hub, Cardiff, UK; 2 Cardiff School of Biosciences, Cardiff University, Cardiff, UK; 3 Max Planck-Cardiff Centre on the Fundamentals of Heterogeneous Catalysis FUNCAT, Cardiff Catalysis Institute, School of Chemistry, Cardiff University; Translational Research Hub, Cardiff, UK

**Keywords:** Heterogeneous catalysis, Chemical engineering, Catalyst synthesis

## Abstract

Gas-liquid-solid catalytic reactions are ubiquitous in the chemical industry and environmental chemistry. The efficacy of these reactions is constrained by the inherently low solubility of gases in liquids and poor mass transfer of reactants/products to/from the catalyst surface. We show that a gas layer trapped on a hydrophobic catalyst (plastron) substantially enriches local gas concentration, alleviating mass-transfer limitations and accelerating reaction rates. Using gold-palladium catalyst for direct hydrogen peroxide synthesis as a model, hydrogen/air plastrons on hydrophobic organosilica yield up to a 20-fold rate enhancement without sub-ambient temperatures, high pressures, or alcohol co-solvents. Combined with selective adsorption of butanethiol on gold-palladium nanoparticles, plastrons further inhibit undesirable decomposition pathways of hydrogen peroxide by repelling water from the catalytic center, eliminating the need for acid and halide promoters. This strategy serves as a new class of heterogeneous catalysts for gas-liquid-solid reactions, offering a route to chemical processes with reduced cost and carbon footprint.

## Abstract (web summary)

Gas–liquid–solid catalytic reactions are often limited by the low solubility of gases in liquids and inefficient mass transport at the catalyst interface. Here the authors demonstrate that a gas layer trapped on a hydrophobic catalyst surface (a plastron) enriches the local gas concentration, reducing mass-transfer constraints and enhancing catalytic performance.

## Introduction

Direct hydrogen peroxide (H_2_O_2_) synthesis can present a sustainable alternative to the current commercial process comprising the sequential hydrogenation and oxidation of an alkylanthraquinone precursor dissolved in a mixture of organic solvents^1,2^. This indirect route is highly capital-intensive, requires significant energy input [198.58 kWh / ton_H2O2_ (27.5%)], and leads to considerable CO_2_ emissions (∼3 kg CO_2eq_ / kg_H2O2_)^1,2^. Furthermore, transportation, storage, and handling of bulk H_2_O_2_ represent a major safety risk and contribute further to the CO_2_ footprint of the commercially sourced oxidant^3^. Despite the progress in catalyst design at the laboratory scale^4–6^, translation to industrially viable direct H_2_O_2_ synthesis processes has remained elusive. Insufficient H_2_O_2_ concentrations and the operational need for H_2_-lean gas mixtures—typically diluted with inert gases (e.g., N_2_, CO_2_) to suppress flammability risks—both compromise productivity and increase process costs^7^.

The rate of direct H_2_O_2_ synthesis in aqueous or alcoholic media is strongly governed by the dissolved gas concentrations, with r_H2O2_ ~[H_2_]^0-1^[O_2_]^0-1^ ^8,9^. The solvent plays a non-innocent mechanistic role: H_2_O_2_ formation is promoted by proton-mediated electron transfer resulting from heterolytic H_2_ activation and subsequent reaction with surface-bound intermediates^7,10^. Given the inherently low solubility of gases in water, elevated pressures ( > 30 bar) are typically required to achieve meaningful reaction rates, thereby raising processing costs^11^.

Hydrophobic interfaces have been exploited in electrochemical systems to improve gas accessibility. Gas-diffusion electrodes can sustain thin gas layers—or plastrons—that facilitate mass transport under imposed potentials and continuous gas feed^12,13^. However, plastrons arise under nonequilibrium conditions specific to electrochemical operation, and their existence or functional relevance in thermocatalytic, liquid-phase H_2_/O_2_ reaction environments is unknown. Whether analogous interfacial H_2_ + O_2_ reservoirs can form spontaneously on hydrophobically modified particulate catalysts, and whether such structures could enhance direct H_2_O_2_ synthesis at near-ambient pressure, remains unexplored.

Inspired by these observations, we hypothesize that the local enrichment of H_2_ and O_2_ at a hydrophobic catalyst surface—beyond concentrations predicted by Henry’s Law—via the formation of an interfacial H_2_ + O_2_ plastron can enhance the rate of H_2_O_2_ production in pure water at near-ambient pressure. To test this hypothesis, we prepared a series of hydrophobic catalysts consisting of AuPd nanoalloys supported on an organosilica support^5,14^. We systematically investigated plastron formation and regeneration on the catalysts, and evaluated its influence on both H_2_O_2_ formation and decomposition. Our results demonstrate that gas plastrons enable efficient direct H_2_O_2_ synthesis in water without the sub-ambient temperatures, elevated pressures, or alcohol co-solvents typically required for high activity and selectivity.

## Results and discussion

### Preparation of hydrophobic catalysts

**Fig. 1:**
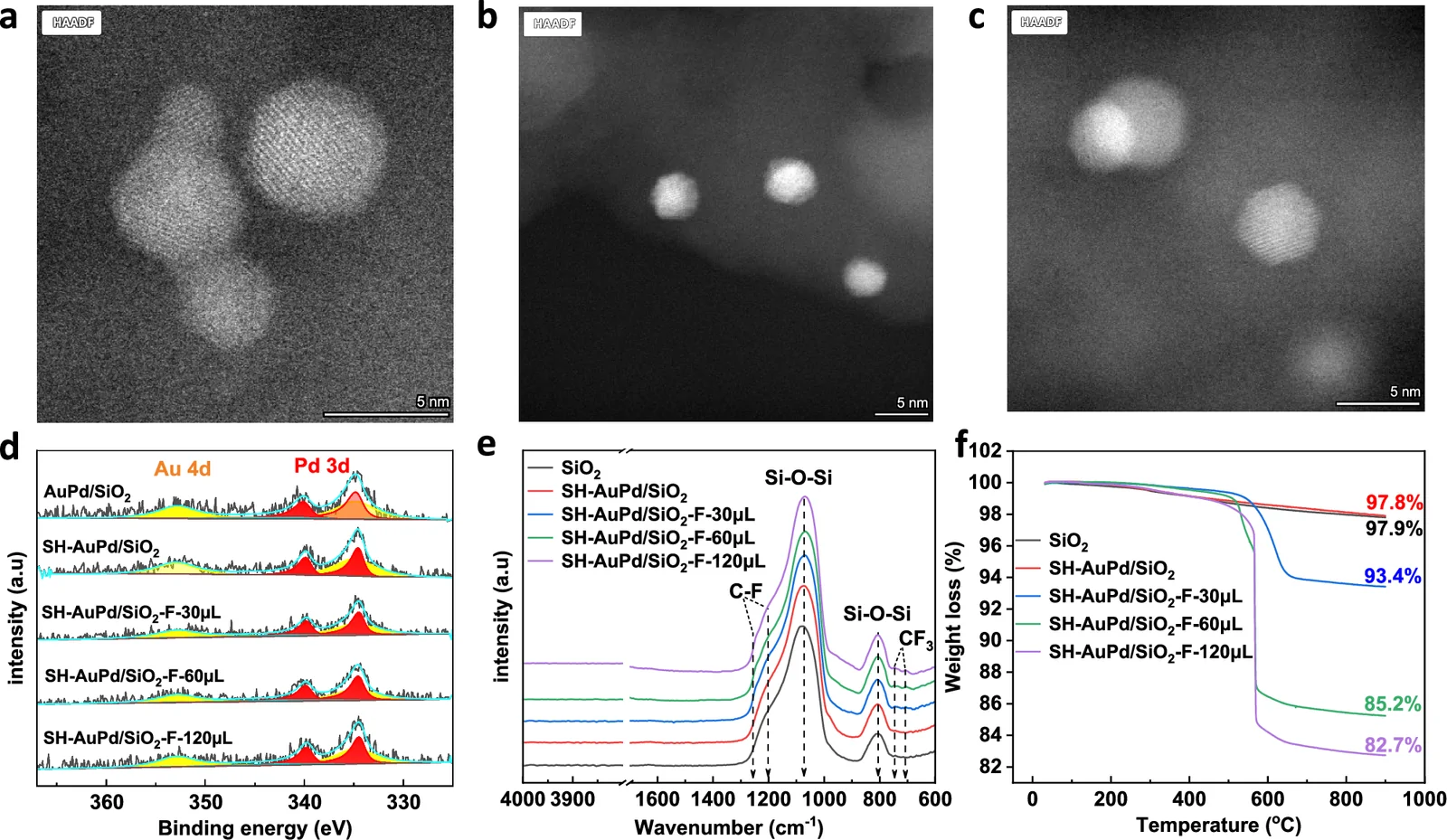
Characterization of catalysts. AC-STEM micrographs of AuPd nanoparticles in (**a**), AuPd/SiO_2_. **b** SH-AuPd/SiO_2_. **c** SH-AuPd/SiO_2_-F-60μL particles. **d** XPS spectra of Au 4 d and Pd 3 d core levels for AuPd/SiO_2_, SH-AuPd/SiO_2_, SH-AuPd/SiO_2_-F-30 μL, SH-AuPd/SiO_2_-F-60 μL, and SH-AuPd/SiO_2_-F-120 μL. **e** FT-IR spectra of pristine SiO_2_, SH-AuPd/SiO_2_, SH-AuPd/SiO_2_-F-30 μL, SH-AuPd/SiO_2_-F-60 μL, and SH-AuPd/SiO_2_-F-120 μL. **f** Thermal profiles of pristine SiO_2_, SH-AuPd/SiO_2_, SH-AuPd/SiO_2_-F-30 μL, SH-AuPd/SiO_2_-F-60 μL, and SH-AuPd/SiO_2_-F-120 μL.

In brief, an AuPd/SiO_2_ catalyst (0.25 wt.% metal loading, Au: Pd = 1:1 by weight, Supplementary Table 1) was prepared by the sol-immobilization method^15^, and grafted with 1H,1H,2H,2H-perfluorooctyltriethoxysilane (PFOTES) and octyltriethoxysilane (OTES) to impart hydrophobicity. Next, self-assembled butanethiol monolayers (SAMs) were deposited using a one-pot immersion protocol (Supplementary Fig. 1a–c)^16–18^. The metal loading and average size of AuPd nanoparticles (4.6 nm) remained unchanged after surface modification relative to the parent AuPd/SiO_2_ catalyst (Fig. 1a–c & Supplementary Fig. 2a–c). Energy-Dispersive X-ray Spectroscopy (EDS) analysis of the catalysts confirmed the formation of a AuPd nanoalloy and the grafting of PFOTES on SH-AuPd/SiO_2_-F-60 μL^19^. Metal immobilization and PFOTES grafting had minimal impact on the specific surface area of the different catalysts ( ~ 200 m^2^/g, Supplementary Table 1).

### Characterization of hydrophobic catalysts

X-ray photoelectron spectroscopy (XPS) confirmed the presence of metallic Pd and Au species on AuPd/SiO_2_, SH-AuPd/SiO_2_, and SH-AuPd/SiO_2_-F-60μL formulations (Fig. 1d)^20^. Thiol functionalization did not induce any detectable changes in the chemical state of either metal. Sulfur, fluorine, and carbon species demonstrated successful functionalization (Supplementary Fig. 3a–d). Fourier Transform Infrared (FT-IR) spectra evidenced the effective surface grafting of PFOTES with characteristic bands at 1170 cm^–1^ and 1245 cm^–1^, indicative of C-F bond vibrations^21,22^, with additional bands also visible at 745 cm^–1^ and 707 cm^–1^ that are consistent with symmetric stretching vibrations of CF_3_ moieties (Fig. 1e)^23,24^. The surface density of PFOTES chains was 0.374, 1.03, and 1.26 groups/nm^2^, respectively, measured from thermogravimetric (TG) analysis between 400 and 800 °C (Fig. 1f)^25^. The contact angles for SH-AuPd/SiO_2_-F-30μL, SH-AuPd/SiO_2_-F-60μL, and SH-AuPd/SiO_2_-F-120 μL in water (110°, 125°, 137°) were substantially higher than that of control SH-AuPd/SiO_2_ catalyst ( ~ 16°) (Supplementary Fig. 4a–d & Supplementary Table 1). These results evidence the effective surface modification and increasing hydrophobicity of the catalysts with higher PFOTES loading.

Analysis by diffuse reflectance infrared Fourier transform spectroscopy, using CO as a probe molecule, was used to assess surface accessibility of metal sites. CO-DRIFTS analysis of AuPd/SiO_2_ and AuPd/SiO_2_-F-60 μL catalysts reveals typical CO adsorption bands on Pd at ~2050–2100 cm^–1^ (linear mode) and ~1800–2000 cm^–1^ (bridged mode), indicating the presence of accessible metallic sites for CO binding (Supplementary Fig. 3e)^26^. In contrast, the thiol-functionalized samples, SH-AuPd/SiO_2_ and SH-AuPd/SiO_2_-F-60 μL, show no detectable CO adsorption features, which is attributed to strong coordination between Pd(0) centers and thiolate ligands, effectively blocking CO access to the active Pd sites^27,28^.

Density functional theory (DFT) calculations further substantiate this interpretation. The results show that butanethiol preferentially adsorbs on Pd sites of the AuPd(111) alloy surface, binding at bridge positions with Pd–S bond distances of ~2.31 Å and a strong adsorption energy of –1.57 eV (Supplementary Fig. 5). This selective chemisorption leads to effective occupation of Pd surface ensembles. The calculated binding configuration therefore provides molecular-level evidence that thiol ligands directly coordinate to Pd atoms, rationalizing the disappearance of CO adsorption bands observed experimentally upon thiol functionalization.

To further support this assignment, vibrational spectra were calculated for both gas-phase butanethiol and adsorbed butanethiol@AuPd(111) (Supplementary Fig. 5). Upon adsorption, the S–H stretching mode present in the gas phase disappears and is replaced by new vibrational features associated with Pd–S interactions, consistent with dissociative chemisorption of the thiol on the metal surface. In addition, the S–C vibrational mode shifts from approximately 900 cm^–1^ in the gas phase to around 1200 cm^-1^ after adsorption. This shift is accompanied by elongation of the S–C bond from 1.845 Å to 1.863 Å, indicating modification of the sulfur electronic environment upon coordination to Pd.

### Computational investigation of the active sites for H_2_O_2_ synthesis

To further clarify the nature of the active sites involved in direct H_2_O_2_ synthesis, density functional theory (DFT) calculations were performed on the AuPd(111) surface to investigate the adsorption behavior of H_2_O_2_, O_2_, H_2_O, and butanethiol (S-but). The calculations reveal that H_2_O_2_ adsorbs most favorably on top of Pd atoms with an adsorption energy of –1.03 eV and a Pd–O distance of 2.412 Å (Supplementary Fig. 6). Upon adsorption, the O–O bond length increases slightly from 1.483 Å in the gas phase to 1.488 Å at the AuPd surface, accompanied by the appearance of new vibrational modes at 500 and 3500 cm^–1^, indicating direct interaction between H_2_O_2_ and Pd surface atoms.

We next investigated the co-adsorption of butanethiol and H_2_O_2_ on AuPd(111). The calculations show that butanethiol preferentially adsorbs at a Pd–Pd bridge configuration, while H_2_O_2_ remains adsorbed on top of a Pd atom, resulting in a total adsorption energy of –2.58 eV (Supplementary Fig. 7). The adsorbed thiolate forms two Pd–S interactions with bond distances of 2.308 and 2.317 Å. Interestingly, the Pd–O distance for H_2_O_2_ decreases from 2.412 Å in the isolated adsorption state to 2.400 Å in the co-adsorbed configuration, suggesting a strengthened metal–adsorbate interaction through synergistic effects between the two adsorbates.

To further probe the active sites involved in the reaction pathway, we compared the co-adsorption of butanethiol with O_2_ and H_2_O on AuPd(111). O_2_ adsorbs preferentially on Pd-rich regions, forming a bridge configuration between neighboring Pd atoms with an adsorption energy of -3.34 eV (Supplementary Fig. 8). The Pd–O bond distances are approximately 2.080 Å, while the O–O bond elongates from 1.240 Å in the gas phase to 1.327 Å upon adsorption, demonstrating efficient activation of molecular oxygen at Pd ensembles. In contrast, H_2_O adsorption is considerably weaker (–1.57 eV), with the molecule adsorbing on top of a Pd atom at a distance of 2.643 Å.

The calculated adsorption energies follow the order: E_ads_(S-but/O_2_) > E_ads_(S-but/H_2_O_2_) > E_ads_(S-but/H_2_O). These results provide strong evidence that Pd surface atoms constitute the primary active sites for both O_2_ activation and H_2_O_2_ adsorption during direct synthesis. Importantly, the calculations further demonstrate that thiol functionalization does not block the ability of Pd ensembles to activate the O–O bond. Instead, butanethiol modifies the local adsorption environment by preferentially destabilizing water adsorption relative to O_2_ and H_2_O_2_. This effect suppresses the formation of surface hydroxyl species that are known to promote H_2_O_2_ decomposition^29^, while simultaneously favoring H_2_O_2_ stabilization at the catalyst interface. Collectively, these findings support the conclusion that direct H_2_O_2_ synthesis predominantly occurs on Pd-rich surface ensembles of the AuPd nanoalloy, whereas the thiol layer acts as a local interfacial modifier that enhances selectivity and suppresses H_2_O_2_ degradation.

### Experimental validation and quantification of plastron formation

**Fig. 2:**
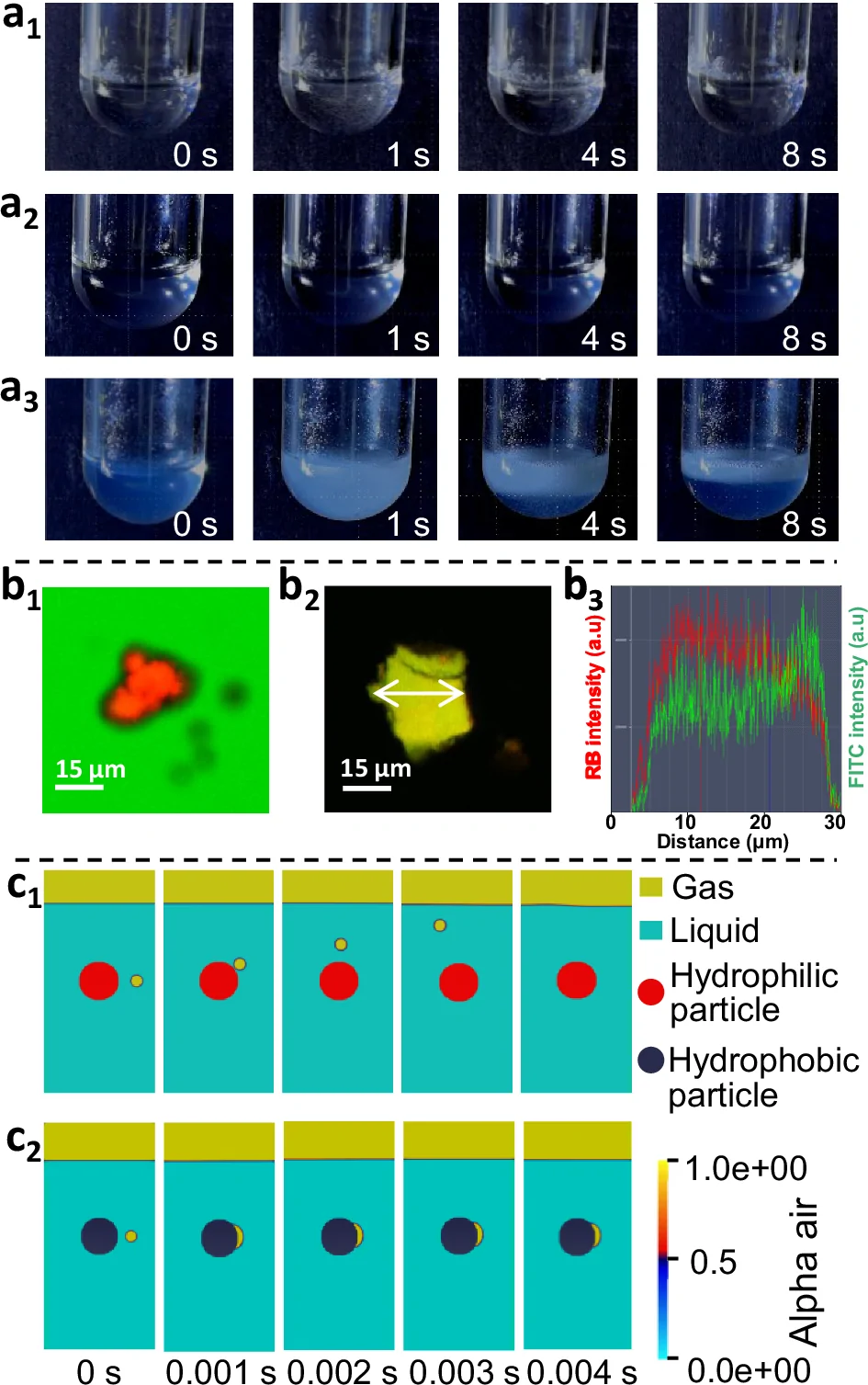
Characterization of plastrons on hydrophobic particles. **a** Optical images showing gas release from **a1**, pure water, **a2**, SH-AuPd/SiO_2_ and **a3**, SH-AuPd/SiO_2_-F-60 μL particles dispersed in water. Experimental conditions: 2 mg particles dispersed in 2 mL pure water, 4% H_2_:air, 3 bar, gas saturation for 20 min at 22 °C followed by depressurization. **b** Confocal micrographs showing **b1**, SiO_2_-F-RB and **b2**, SiO_2_-RB particles immersed in aqueous FITC solution, and **b3**, fluorescent intensity across the particle (white line in b2). The dark area, devoid of fluorescence, delineates the boundary between red hydrophobic particle and green FITC solution in (b1). **c** Simulated gas bubble migration dynamics in water with **c1**, hydrophilic particle (red, contact angle 16°) and **c2**, hydrophobic particle (purple, contact angle 125°).

To investigate plastron formation, different hydrophobic catalyst dispersions were prepared in pure water by ultrasonication using a pressure-resistant tube (Supplementary Fig. 1d & Supplementary Movie 1). The sealed tube was saturated with a 4% H_2_:air mixture at 3 bar, followed by depressurization. Only a few bubbles were generated in a control experiment without the catalyst and when using the hydrophilic SH-AuPd/SiO_2_ catalyst (Fig. 2a1, a2). In contrast, a dispersion containing hydrophobic SH-AuPd/SiO_2_-F-60μL particles shows a dramatic increase in bubble generation immediately after depressurization (Fig. 2a3). This behavior is attributed to the release of gas trapped in plastrons (gas layers, Supplementary Fig. 1e) formed on the surface of SH-AuPd/SiO_2_-F-60μL, which expand and detach as visible bubbles upon pressure release.

Beyond qualitative visualization, the plastron thickness was quantitatively estimated using a buoyancy-gravity equilibrium model (Supplementary Section S1.5). Based on the measured flotation behavior of the particles and the density difference between gas and liquid phases, the average plastron thickness surrounding the 200 nm silica particles was calculated to be approximately 30 nm. This thickness is consistent with a stable nanoscale gas layer capable of locally enriching dissolved reactant gases at the catalyst interface.

The temporal evolution and stability of the plastron were further quantified by monitoring the light transmittance (turbidity) of catalyst suspensions over time (Supplementary Fig. 9d). The SH-AuPd/SiO_2_-F-60 μL particles maintain nearly constant turbidity over at least 30 min, demonstrating persistent suspension stability associated with stable plastron retention. In contrast, the hydrophilic SH-AuPd/SiO_2_ particles progressively sediment within 30 min due to the absence of a stabilizing gas layer. Plastron stability was quantified as the ratio between the absorbance after 30 min and the initial absorbance, showing a clear positive correlation with particle hydrophobicity (Supplementary Fig. 9g, h). Zeta potential measurements revealed lower negative surface charge for SH-AuPd/SiO_2_ than SH-AuPd/SiO_2_-F-60μL (-36 mV *vs* -24 mV) (Supplementary Fig. 9e). This finding indicates that plastron formation, rather than electrostatic repulsion, plays a dominant role in maintaining dispersion stability in aqueous media. To investigate the relationship between hydrophobicity and plastron stability, two additional catalysts were synthesized, designated as SH-AuPd/SiO_2_-F-15 μL and SH-AuPd/SiO_2_-F-45 μL. The corresponding contact angle images are provided in Supplementary Fig. 4b, d. The correlation between hydrophobicity and plastron stability is illustrated in Supplementary Fig. 9f.

SH-AuPd/SiO_2_-F-30μL generate fewer bubbles than SH-AuPd/SiO_2_-F-60 μL, which dissipate within 8 s, consistent with its lower hydrophobicity and reduced plastron stability. The SH-AuPd/SiO_2_-F-120 μL formulation, despite exhibiting the highest contact angle, also shows less bubble release, because the particles float at the air-water interface suppressing effective plastron formation around suspended particles (Supplementary Fig. 9a–c).

Confocal microscopy was additionally used to directly visualize plastron formation on catalyst surfaces^30^. Hydrophilic and hydrophobic silica particles – modified with Rhodamine B (red fluorescence) to yield SiO_2_-RB and SiO_2_-F-RB, respectively – were dispersed into an aqueous solution of fluorescein isothiocyanate (FITC, green fluorescence). A distinct black halo devoid of fluorescence was observed surrounding the SiO_2_-F-RB particles (Fig. 2 b 1), indicating the presence of an interfacial air layer between the red fluorescent particle (hydrophobic) and surrounding aqueous phase (green FITC solution). In contrast, SiO_2_-RB particles (hydrophilic) display yellow fluorescence resulting from the overlap of red (Rhodamine B) and green (FITC) emissions against a non-luminous background (Fig. 2b2), confirming direct contact between the particle surface and the surrounding aqueous solution with no plastron formation. The intensity profile across the scan line (Fig. 2b3) shows maximum red intensity at the particle center and a gradual decrease toward the edges, inversely correlated with green intensity.

While advanced operando methods such as in-situ AFM could in principle provide additional interfacial information, their implementation under the present reaction conditions remains experimentally challenging because freely diffusing nanoparticles suspended in bulk liquid cannot currently be tracked with sufficient spatial and temporal resolution under reactive gas-liquid-solid conditions. Therefore, the combined use of buoyancy-based thickness estimation, time-resolved turbidity measurements, bubble-release analysis, and confocal microscopy provides a robust and experimentally accessible characterization of plastron formation, stability, and temporal evolution in the present catalytic system.

### Simulation of plastron formation

To support these findings, we conducted 2D numerical simulations on plastron formation in an air-water system using the volume of fluid (VOF) method^31^. Two types of particles were modeled: hydrophilic (contact angle 16°, representative of SH-AuPd/SiO_2_) and hydrophobic (contact angle 125°, representative of SH-AuPd/SiO_2_-F-60μL). For the hydrophilic particle, a bubble approaching the surface at 0.001 s slides past it and merges with the air-water interface by 0.004 s (Fig. 2c1). This behavior is driven by capillary forces pushing the bubble toward the surface in the microchannel. However, due to the low contact angle, the reduction in total surface energy upon bubble attachment is minimal. In this case, viscous resistance between the bubble and the particle dominates over capillary pinning, preventing formation of a stable contact line and hence no plastron is formed. In stark contrast, the hydrophobic particle immediately anchors the bubble within 0.001 s, resulting in the formation of a stable gas film (Fig. 2c2; meshing details in Supplementary Fig. 10a, b). Here, the higher contact angle leads to a substantial reduction in surface energy upon bubble attachment, while the capillary pinning force exceeds viscous resistance, stabilizing the bubble-solid interface and facilitating plastron formation. The gas film remains stable up to at least 0.035 s (Supplementary Fig. 10c). Analysis of velocity streamlines at 0.035 s reveals two almost symmetric major recirculation zones flanking the attached bubble (Supplementary Fig. 10d), alongside two weaker secondary vortices at the bubble flanks. These patterns reflect transitions between clockwise and counterclockwise flows, which help stabilize the plastron. Notably, the upper recirculation zone is larger than the lower one, suggesting that buoyancy forces accelerate the flow near the bubble’s upper contact point. However, due to strong capillary anchoring enabled by the high contact angle, the bubbles remain attached to the surface, preserving a stable plastron.

### Catalytic performance under plastron formation

**Fig. 3:**
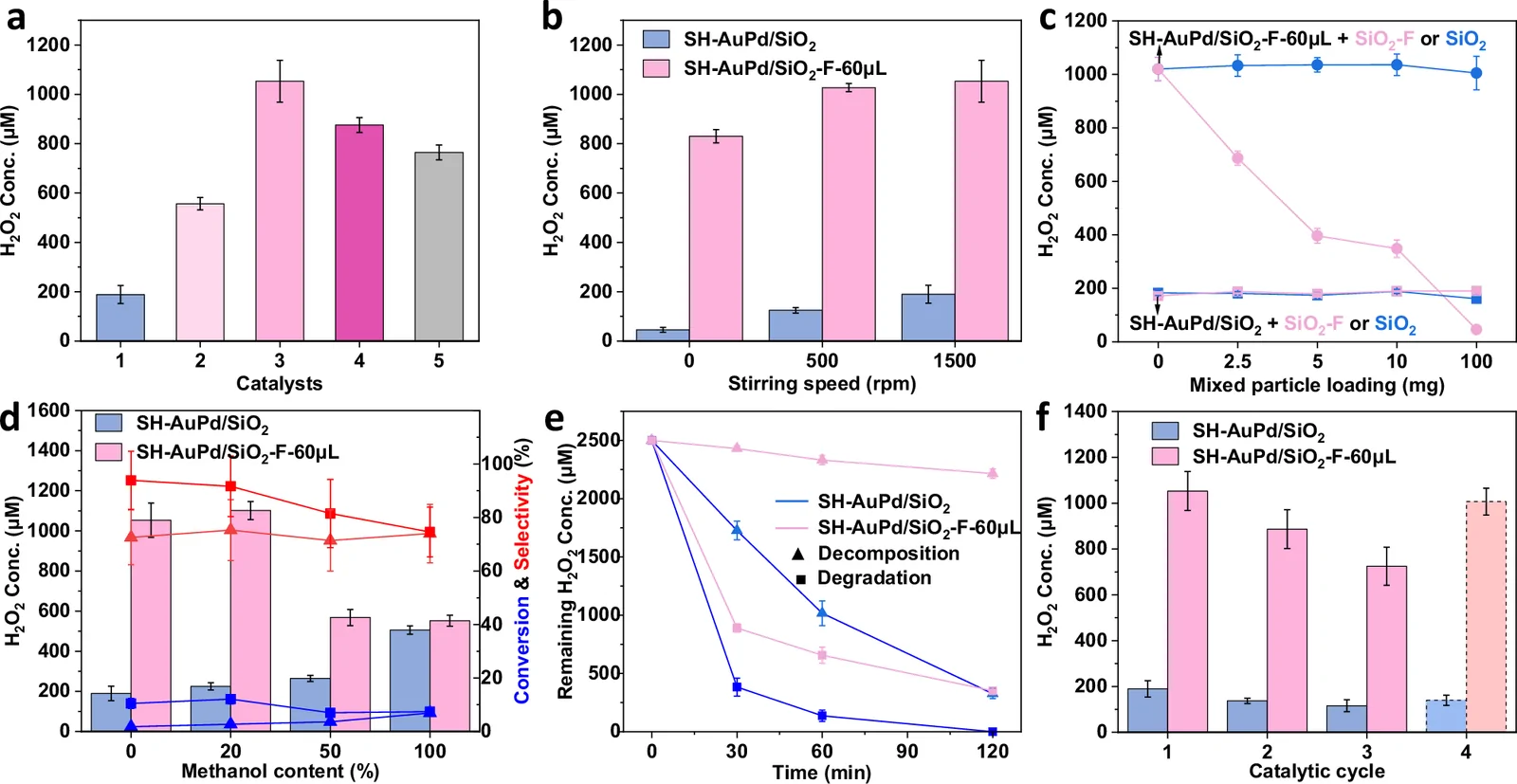
Catalytic results with/without plastron. **a** H_2_O_2_ concentration for: **a1**, SH-AuPd/SiO_2_, **a2**, SH-AuPd/SiO_2_-F-30 μL, **a3**, SH-AuPd/SiO_2_-F-60 μL, **a4**, SH-AuPd/SiO_2_-F−120 μL, and **a5**, SH-AuPd/SiO_2_-C. Reaction conditions: H_2_:Air = 4%, 3 bar, 22 °C, 30 min, 2.5 mg catalyst, 5 mL of deionized water, 1500 rpm. **b** H_2_O_2_ concentration for SH-AuPd/SiO_2_ and SH-AuPd/SiO_2_-F-60μL at variable stirring rates. Reaction conditions: H_2_:Air  = 4%, 3 bar, 22 °C, 30 min, 2.5 mg catalyst, 5 mL of deionized water, variable stirring rates. **c** H_2_O_2_ concentration for SH-AuPd/SiO_2_ and SH-AuPd/SiO_2_-F-60 μL particles combined with noncatalytic particles. Reaction conditions: H_2_:Air = 4%, 3 bar, 22 °C, 30 min, using either 2.5 mg of SH-AuPd/SiO_2_ or 2.5 mg of SH-AuPd/SiO_2_-F-60 μL combined with various amounts (2.5, 5, 10, 100 mg) of noncatalytic SiO_2_-F or SiO_2_ particles. **d** H_2_O_2_ concentration, H_2_ conversion, and H_2_O_2_ selectivity in methanol/water mixtures with schematic illustration of plastron behavior at variable particle wettability. Reaction conditions: H_2_:Air = 4%, 3 bar, 22 °C, 30 min, 5 mL of methanol/water mixture. **e** Time-evolution of H_2_O_2_ decomposition and degradation (Triangles, decomposition; Squares, degradation). Reaction conditions: 2.5 mg of catalyst, 5 mL of deionized water with 2500 μM H_2_O_2_, 3 bar air (decomposition) or 3 bar H_2_:N_2_ = 4% (degradation), 22 °C, 1500 rpm, 30, 60, and 120 min. **f** Recyclability and reuse of SH-AuPd/SiO_2_ and SH-AuPd/SiO_2_-F-60 μL particles. Reaction conditions: H_2_:Air = 4%, 3 bar, 22 °C, 30 min, 2.5 mg catalyst, 5 mL of deionized water, 1500 rpm. run 4 was carried out after re-doping the spent catalyst from run 3 with the same concentration of butanethiol as fresh catalyst. Error bars represent standard deviation.

We subsequently evaluated the performance of the catalysts for direct H_2_O_2_ synthesis in water at ambient temperature and 3 bar using a 4% H_2_/air mixture – conditions that lie safely outside the explosive region (13-59%) (Fig. 3a)^32^. The H_2_O_2_ concentration increased monotonically with the catalyst hydrophobicity, from 190 μM for non-hydrophobic SH-AuPd/SiO_2_ catalyst to 560 μM and 1050 μM for SH-AuPd/SiO_2_-F-30μL and SH-AuPd/SiO_2_-F-60μL, respectively (Fig. 3a1-a3). However, the most hydrophobic catalyst, SH-AuPd/SiO_2_-F-120μL, produced a lower concentration (870 μM), attributed to its flotation on water (Fig. 3a4). For comparison, SH-AuPd/SiO_2_-C grafted with OTES chains with a contact angle of 120° (Supplementary Fig. 4e)—yielded lower H_2_O_2_ concentration compared to SH-AuPd/SiO_2_-F-60μL (760 μM) with a slightly higher contact angle (125°) (Fig. 3a5). Two additional particles, SH-AuPd/SiO_2_-F-15μL and SH-AuPd/SiO_2_-F-45μL, with different degrees of hydrophobicity, exhibit catalytic activities of 290 μM and 750 μM, respectively, in agreement with the observed trend (Supplementary Fig. 9f). The H_2_O_2_ concentration for SH-AuPd/SiO_2_-F-60μL displayed a bell-shaped curve with catalyst loading, which is attributed to competitive H_2_O_2_ degradation activity (Supplementary Fig. 11a).

The SH-AuPd/SiO_2_ catalyst exhibits minimal activity towards H_2_O_2_ synthesis under static conditions due to prominent mass transfer limitations (Fig. 3b). Even upon stirring at 500 rpm, H_2_O_2_ concentration remains low at 125 μM, increasing only slightly to 190 μM at 1500 rpm. In contrast, SH-AuPd/SiO_2_-F-60 μL achieves 830 μM without stirring. Upon stirring, the H_2_O_2_ concentration increases modestly to 1020 μM at 500 rpm and remains nearly constant at 1050 μM even at 1500 rpm, highlighting the dominant role of plastron-mediated mass transport over mechanical agitation.

To further elucidate the role of gas plastrons in direct H_2_O_2_ synthesis and exclude any possible promotion effects from F-containing PFOTES grafted species^33,34^, we performed reactions using mixtures of SH-AuPd/SiO_2_ with either SiO_2_-F or SiO_2_ particles (Fig. 3c). No significant change in H_2_O_2_ concentration ( ~ 180 μM) was observed for SiO_2_-F or SiO_2_ at variable loadings, indicating that both particles do not hinder gas accessibility to SH-AuPd/SiO_2_, even if gas plastrons form on the SiO_2_-F surface. Additional control experiments revealed no activity toward H_2_O_2_ formation or degradation for both SiO_2_-F and SiO_2_ (Supplementary Fig. 11b). Collectively, these findings confirm that the observed promotive effect on SH-AuPd/SiO_2_-F-60μL does not result from halide-induced promotion of AuPd surfaces. Further addition of 10 mg of SiO_2_-F to a dispersion of SH-AuPd/SiO_2_-F-60μL led to a sharp decrease in H_2_O_2_ concentration from 1050 μM to 350 μM. Increasing the SiO_2_-F amount to 100 mg resulted in a further drop to 44 μM. This decline is attributed to competition for plastron formation between the inert SiO_2_-F particles and the active SH-AuPd/SiO_2_-F-60μL catalysts, which reduced the availability of gases for the reaction, with an observed decrease in H_2_ conversion consistent with this observation (Supplementary Fig. 11c). Conversely, the catalytic activity remained unaffected when SH-AuPd/SiO_2_-F-60μL was combined with hydrophilic SiO_2_ particles, consistent with the fact that unmodified SiO_2_ particles cannot induce plastron formation.

Plastron formation on SH-AuPd/SiO_2_-F-60μL was discouraged by introducing methanol as a low-surface tension co-solvent (22.5 mN/m) to decrease the contact angle (Fig. 3d & Supplementary Fig. 4f–i). Introducing 20% methanol slightly increased the H_2_O_2_ concentration from 1053 μM to 1100 μM. However, a sharp decline to 550 μM occurred when pure methanol was used. This was attributed to the disruption of plastron formation (Fig. 3d) preventing stable gas layer retention on the particle surface^35^. Interestingly, dropping methanol into a SH-AuPd/SiO_2_-F-60μL aqueous dispersion led to gas plastron release (Supplementary Fig. 5c2 & Supplementary Movie 2). This was not observed for SH-AuPd/SiO_2_ catalyst aqueous dispersion (Supplementary Fig. 5 c 4). For this catalyst, H_2_O_2_ production increased from 190 to 505 μM as methanol content increased owing to the higher gas solubility and no plastron formation^36^. For comparison, isopropanol with also low surface tension (22.9 mN/m) and lower contact angles (Supplementary Fig. 4j–m) was also used to check the generality. A similar trend was also observed, as shown in Supplementary Fig. 11d.

**Fig. 4:**
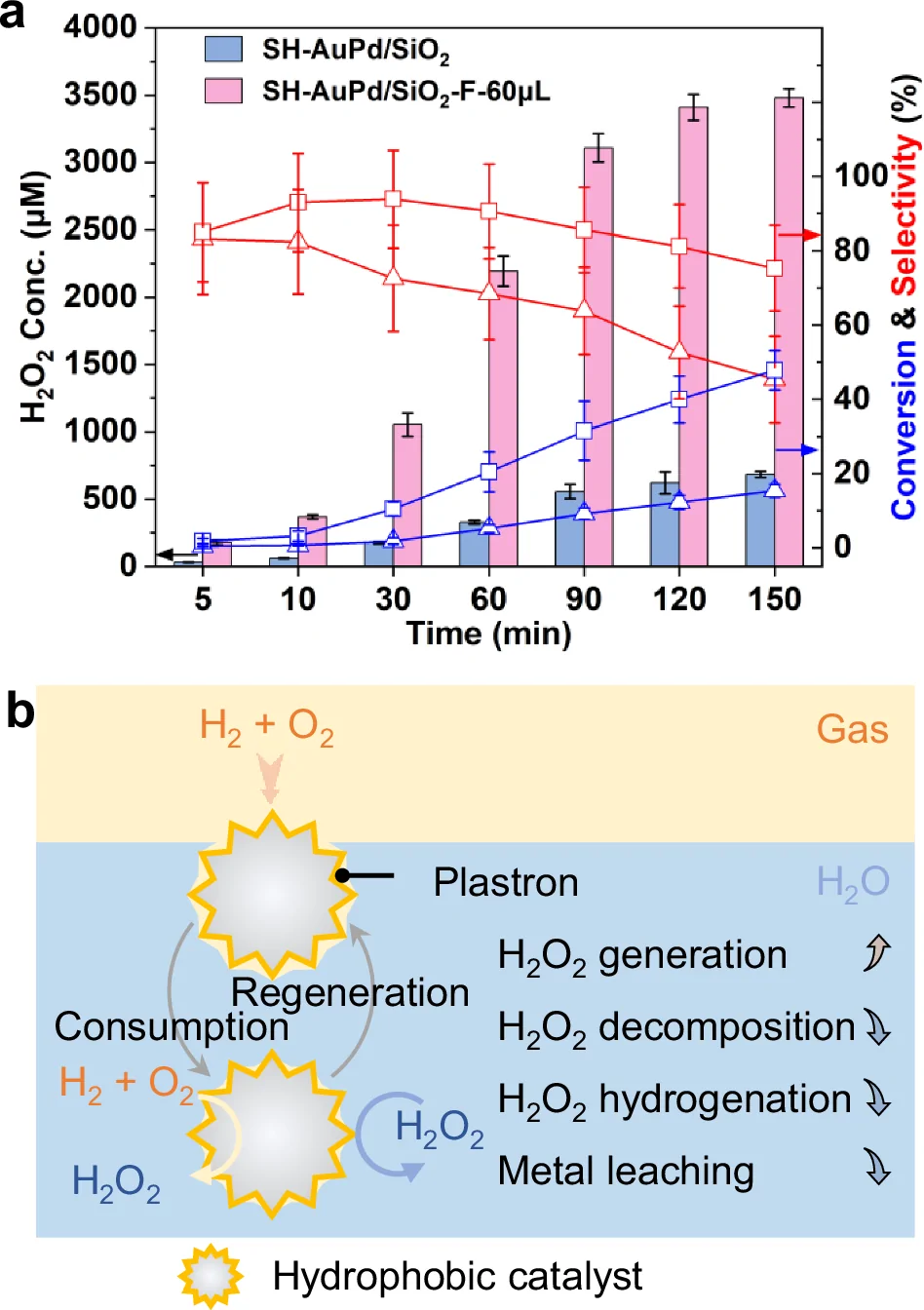
Kinetic profiles for H_2_O_2_ synthesis and proposed reaction process with plastron on hydrophobic catalysts. **a** Kinetic profiles for H_2_O_2_ direct synthesis for SH-AuPd/SiO_2_ and SH-AuPd/SiO_2_-F-60μL particles. Conversion and selectivity (triangle, SH-AuPd/SiO_2_; squares, SH-AuPd/SiO_2_-F-60μL). Reaction conditions: 2.5 mg of catalyst, 5 mL of deionized water, 3 bar H_2_:Air = 4%, 22 °C, 1500 rpm, variable times (5, 10, 30, 60, 90, 120, 150 min). **b** The advantages of the plastron catalysis in the direct synthesis of H_2_O_2_. Error bars represent standard deviation.

We monitored the kinetic profiles of H_2_O_2_ formation over the SH-AuPd/SiO_2_ and SH-AuPd/SiO_2_-F-60 μL catalysts (Fig. 4a). The H_2_O_2_ concentration increased almost linearly within 90 min for SH-AuPd/SiO_2_-F-60 μL, indicating sustained catalyst activity and stability throughout the reaction that coincides with plastron formation exhibiting a protective role reducing metal leaching, observed to be four times lower compared to SH-AuPd/SiO_2_ without plastron protection (Supplementary Table 2). Correspondingly, the H_2_O_2_ productivity reached 1900 mol_H2O2_ kg_metal_^–1^ h^–1^ for SH-AuPd/SiO_2_-F-60 μL, substantially higher than 300 mol_H2O2_ kg_metal_^–1^ h^–1^ for SH-AuPd/SiO_2_. The gradual decrease in selectivity after 30 min is mainly attributed to the progressive degradation of the generated H_2_O_2_. This pronounced activity is attributed to gas plastrons on SH-AuPd/SiO_2_-F-60μL, facilitating H_2_ and O_2_ transfer from the reactor headspace to the catalyst surface, thereby maintaining high local concentration of reactant gases. In addition, at a fixed H_2_ conversion of ~10%, SH-AuPd/SiO_2_-F-60μL shows a higher selectivity of 94% compared with SH-AuPd/SiO_2_ (64%). The presence of the plastron layer limits the direct interaction between H_2_O_2_ and the catalytic active sites, suppressing its subsequent degradation. We also evaluated the extent of H_2_O_2_ (2500 μM) decomposition and degradation over both catalysts (Fig. 3e). SH-AuPd/SiO_2_-F-60μL exhibits lower decomposition activity compared to the SH-AuPd/SiO_2_ analog. Similarly, H_2_O_2_ degradation rates for SH-AuPd/SiO_2_-F-60μL were found to be lower than for SH-AuPd/SiO_2_. Collectively, these findings highlight the critical role of gas plastrons in shielding catalytic sites from direct interaction with H_2_O_2_ in solution, thereby suppressing H_2_O formation and enhancing catalyst durability and performance^12^.

The apparent activation energies were obtained from Arrhenius plots based on the initial H_2_O_2_ formation rates measured at different temperatures. For the SH-AuPd/SiO_2_-F-60 μL catalyzed plastron system, the H_2_O_2_ production rate increased at higher temperatures (Supplementary Fig. 11e) indicating kinetically controlled behavior. In contrast, for SH-AuPd/SiO_2_ without plastron, the H_2_O_2_ production rate remained nearly constant over the investigated temperature range (Supplementary Fig. 11f), suggesting that the reaction is controlled by gas-liquid mass transfer rather than intrinsic surface kinetics. The corresponding apparent activation energies are presented in Supplementary Fig. 11g. The distinct difference in the activation energies between both systems demonstrates that the plastron configuration effectively reduces gas-liquid mass transfer resistances, thereby enabling intrinsic kinetic control.

The SH-AuPd/SiO_2_-F-60μL formulation was recycled twice. The net H_2_O_2_ concentration declined from 1050 μM to 880 μM and further to 700 μM. A similar deactivation trend was observed for the SH-AuPd/SiO_2_ catalyst, albeit with significantly lower absolute concentrations of 190, 140, and 120 μM, respectively. This deactivation can be attributed to moderate leaching of bonded thiol groups during washing and drying^37^. This interpretation is supported by an increase in the H_2_ conversion for the spent catalysts owing to enhanced gas-metal interactions as a result of reduced thiol group coverage; similarly, the H_2_O_2_ selectivity declined, which can be interpreted by the direct interaction between the metal and H_2_O_2_ (Supplementary Fig. 11h). This interpretation is supported by CO-DRIFTS analysis of the spent catalysts pointing out increased CO adsorption after use (Supplementary Fig. 3f). AC-STEM imaging and XPS analysis of the once-used spent catalyst revealed negligible variation in metal nanoparticle size and the chemical state (Supplementary Fig. 2d & Supplementary Fig. 3g, h). Importantly, re-doping the thrice-used SH-AuPd/SiO_2_-F-60 μL catalyst with butanethiol restored the catalytic activity, supporting this explanation (Fig. 3f). The recycled catalyst exhibits no significant differences in surface hydrophobicity, surface zeta potential, or particle size, as shown in Supplementary Figs. 4h, 5e and i.

We benchmarked the catalytic performance of SH-AuPd/SiO_2_-F-60μL against conventional catalysts for direct H_2_O_2_ synthesis at mild reaction conditions (**Entries 2-5;** Supplementary Table 3)^38–40^. The H_2_O_2_ productivity of SH-AuPd/SiO_2_-F-60μL is 1.5- to 30-fold higher than that of benchmark catalysts after normalizing by the metal content (1900 mol_H2O2_ kg_metal_^–1^ h^–1^ vs 66-1300 mol_H2O2_ kg_metal_^–1^ h^–1^). This catalyst also exceeds the performance of catalysts operated under conditions widely utilized for H_2_O_2_ direct synthesis (alcohol co-solvent, sub-ambient temperatures and elevated pressures) (**Entries 6-10;** Supplementary Table 3). Notably, under identical conditions, our previously reported AuPd/TiO_2_ catalyst produced no detectable H_2_O_2_ (Supplementary Fig. 11i)^37^.

Forming a gas layer between a hydrophobic catalyst and water combined with a butanethiol promoter not only significantly enhances gas accessibility but also effectively suppresses metal leaching, a primary cause of catalyst deactivation. Additionally, preferential extraction of water from the catalytic center enhances H_2_O_2_ stability against decomposition (Fig. 4b), providing a promising strategy to mitigate over-oxidation in a broad range of reactions. Beyond H_2_O_2_ synthesis, we consider that this plastron-enabled platform offers broad applicability to a range of gas-solubility-limited processes including those used in electrochemical and thermally-driven reactions. Overall, this work opens a new avenue for designing robust and selective catalytic systems through interface engineering.

## Methods

### Preparation of hydrophobic particles

Loading of AuPd alloy nanoparticles over SiO_2_: 0.403 mL of HAuCl_4_ ([Au] =12.4 mg/mL), 0.5 mL of PdCl_2_ ([Pd] = 10 mg/mL), and 1 mL of PVA (10 mg/mL, 1:1 w/w (Au+Pd): PVA) aqueous solutions were added to 140 mL of water. After stirring for 10 min, a 0.1 mM aqueous solution of NaBH_4_ (Au+Pd)/NaBH_4_ = 1: 10 mol/mol was added to the yellow solution under vigorous magnetic stirring. A black AuPd alloy sol was immediately formed. The colloidal suspension was stirred for 30 min. Then, 4 g of Aerosil^®^ 200 was added to the suspension, and the pH was decreased to 2 by adding concentrated sulfuric acid. After aging for 2 h, the suspension was filtered and washed with 1.5 L of deionized water, and the catalyst was dried at 110 °C (16 h) and calcined at 400 °C under air flow [100 mL(STP)/min] for 3 h at a heating rate of 10 °C/min. The calcined catalyst was subsequently reduced at 400 °C under 5% H_2_/Ar with a flow rate of 100 mL(STP)/min atmosphere for 3 h at a rate of 10 °C/min. The prepared catalyst was denoted as AuPd/SiO_2_ (Supplementary Fig. 1a).

Surface modification of AuPd/SiO_2_ particles: 0.5 g AuPd/SiO_2_ was dispersed into 30 mL of toluene in a 100-mL round-bottom flask, and 15, 30, 45, 60, or 120 µL of PFOTES were added. 2 mg PTSA was added as the catalyst. The mixture was heated to 112 °C in an oil bath for 1.5 h. After the reaction, the dispersion was filtered and washed with ethanol, and the filter cake was dried at 110 °C overnight. The modified catalyst was denoted as AuPd/SiO_2_-F-XμL, where X = 15, 30, 45, 60, or 120. The same protocol was used to graft (non-fluorinated) alkyl chains on AuPd/SiO_2_ using 60 µL of OTES instead of PFOTES. The modified catalyst was denoted as AuPd/SiO_2_-C (Supplementary Fig. 1b).

Self-assembled butanethiol monolayers of AuPd/SiO_2_ and AuPd/SiO_2_-F-XμL particles: 0.4 g of the as-prepared AuPd/SiO_2_ and AuPd/SiO_2_-F-XμL catalysts were dispersed into 20 mL of ethanol and 10 µL of butanethiol were added. This dispersion was stirred at 22 °C for 4 hours. After the reaction, the dispersion was filtered, washed with ethanol, and the filter cake was dried at 22 °C under vacuum. Both catalysts were denoted as SH-AuPd/SiO_2_ and SH-AuPd/SiO_2_-F-XμL, respectively (Supplementary Fig. 1c)^41^.

Preparation of SiO_2_-RB and SiO_2_-F-RB particles: A mixture consisting of 0.3 g of SiO_2_, 2 µL of APTES, 2 mg of PTSA, and 4 mg of RBITC was dispersed in 20 mL of toluene in a 50-mL round-bottom flask. The reaction was carried out at 112 °C for 1.5 h. After the reaction, the dispersion was filtered, and the resulting solid was washed with ethanol. The collected filter cake was subsequently dried at 110 °C overnight, yielding a pink solid denoted as SiO_2_-RB^42^. The same protocol was adapted to prepare RB-containing particles functionalized with fluorinated chains by including 36 µL of PFOTES in the initial dispersion. The sample was denoted as SiO_2_-F-RB.

### Plastron release tests

To investigate plastron formation, catalyst dispersions were prepared in pure water by ultrasonication using a 15-mL pressure-tolerance tube, 2 mg prepared catalysts were dispersed in 2 mL of water using a Sonics VCX-750 ultrasonic processor equipped with a tapered probe operating at 40% amplitude in pulsed mode (1 s on / 1 s off) for 5 s. The dispersion was purged with 4% H_2_/air (3 bar) for 20 min to saturate the aqueous phase, followed by rapid pressure release. The release of plastron was also demonstrated by dropping methanol into the particle aqueous dispersion. The catalyst dispersions were prepared by the aforementioned method. 100 µL of methanol was dropped into the catalyst dispersions at ambient pressure. The observation was shown in Supplementary Fig. 5c.

### Plastron stability tests

The light absorbance of particle dispersions was employed as a quantitative descriptor of plastron stability. Specifically, 2.5 mg of each particle sample was dispersed in 5 mL of water, and 3 mL of the resulting suspension was transferred to a UV-Vis cuvette for absorbance measurement at 600 nm. The corresponding absorbance values are presented in Supplementary Fig. 9g, h. Plastron stability was defined as the ratio of the absorbance at 30 min to that at 0 min, expressed as a percentage. The correlation between hydrophobicity and plastron stability is shown in Supplementary Fig. 9f.

### Catalytic tests

H_2_ and air were mixed in a cylinder with different ratios. Deionized water and different amounts of the given catalyst were added into a 15 mL pressure-tolerance tube with a magnetic stirrer. Prior to the reaction, the catalyst was dispersed in DI water using a Sonics VCX-750 ultrasonic processor equipped with a tapered probe at 40% amplitude in pulsed mode (1 s on / 1 s off) for 5 s. The tube was purged 3 times before the reaction. The reaction started when the tube was charged with the mixed gas at a given pressure. After the reaction, the catalyst was filtered with a syringe filter. The H_2_O_2_ concentration in the recovered liquid samples was analyzed using titanium oxalate method. Briefly, 1.5 mL of a potassium titanyl oxalate sulfuric acid solution was mixed with an equal volume filtrate^43^. Formation of a yellow peroxo-titanic acid complex was measured spectrophotochemically at 400 nm to calculate the H_2_O_2_ concentration using an external calibration method^44^. Besides, the catalytic activity results with error bars are reported as mean ± standard deviation from triplicate experiments.

The catalytic activity of the catalyst is evaluated from the H_2_O_2_ concentration after the reaction. The catalytic productivity was calculated as follows

1 $${{{\rm{P}}}}=\frac{{{{{\rm{n}}}}}_{{{{\rm{H}}}}2{{{\rm{O}}}}2}}{{{{{\rm{m}}}}}_{{{{\rm{cat}}}}}*{{{\rm{w}}}}*{{{\rm{t}}}}}$$

where P is the H_2_O_2_ productivity, n denotes the amount of H_2_O_2_ produced (in moles), m is the mass of the catalyst, w represents the metal loading (by weight) in the catalyst, and t is the reaction time.

The H_2_ conversion and H_2_O_2_ selectivity were determined using gas chromatography (GC). Upon completion of the reaction, the unreacted gases were collected using a syringe and subsequently analyzed by GC using a Varian gas chromatograph (GC) equipped with a CPSIL5CB column (50 m × 0.33 mm i.d.) and a flame ionization detector (FID).

H_2_ conversion (Eq.( 2)), H_2_O_2_ selectivity (Eq.( 3)) and H_2_O_2_ production rate (Eq.( 4)) were defined as follows:

2 $${{{{\rm{H}}}}}_{2}{{{\rm{Conversion}}}}=\frac{{{{{\rm{mmol}}}}}_{{{{\rm{H}}}}2({{{\rm{t}}}}0)}\,-{{{{\rm{mmol}}}}}_{{{{\rm{H}}}}2({{{\rm{t}}}}1)}}{{{{{\rm{mmol}}}}}_{{{{\rm{H}}}}2({{{\rm{t}}}}0)}}\times 100\%$$

3 $${{{{\rm{H}}}}}_{2}{{{{\rm{O}}}}}_{2}{{{\rm{Selectivity}}}}\left(\%\right)=\frac{{{{{\rm{H}}}}}_{2}{{{{\rm{O}}}}}_{2}{{{\rm{detected}}}}\left({{{\rm{mmol}}}}\right)}{{{{{\rm{H}}}}}_{2}{{{\rm{consumed}}}}\left({{{\rm{mmol}}}}\right)}\times 100\%$$

### Apparent activation energy measurements

A total of 2.5 mg of SH-AuPd/SiO_2_ or SH-AuPd/SiO_2_-F-60 μL particles was dispersed in 5 mL of water via sonication. The reaction conditions were identical to those employed for the direct synthesis of H_2_O_2_. The reaction was conducted at four different temperatures: 273, 293, 313, and 333 K. The concentration of H_2_O_2_ was determined at reaction times of 10, 20, and 30 min. The apparent activation energy was derived from Arrhenius plots based on the initial H_2_O_2_ formation rates, as presented in Supplementary Fig. 11e–j.

### Characterization of hydrophobic particles

Rhodamine B-modified silica particles were visualized in a confocal microscope using glass well slides. Particles were carefully immobilized onto the concave surface of the wells and 10 µL of an aqueous FITC solution (100 ppm) was added. The preparation was cover slipped, and the edges were sealed using clear nail varnish to prevent any possible leakage or evaporation.

The Fourier-transformed infrared spectra (FT-IR) of the different particles were measured from 500 to 4000 cm^–1^ on a Bruker Tensor 27 spectrometer equipped with a HgCdTe (MCT) detector and operated with OPUS software. The spectra were measured after 256 scans.

The metal loading in the catalysts was measured by microwave plasma atomic emission spectroscopy (MP-AES) using an Agilent Technologies 4200 instrument using a three-point calibration. Before the analyses, 10 mg of the given catalyst was digested in 1 mL of concentrated *aqua regia* for 16 h in a 10 mL volumetric flask. After digestion, the solution was diluted to 10 mL and filtered using a hydrophilic syringe filter.

The total metal leaching from supported catalysts was quantified through the analysis of post-reaction solutions using an Agilent 7900 ICP-MS equipped with an I-AS auto-sampler using 5-point calibration using certified reference materials from Perkin Elmer and certified internal standards from Agilent. All calibrants were matrix matched.

The morphology and particle size distribution of the catalysts were inspected by high-angle annular dark-field scanning transmission electron microscopy (HAADF-STEM) and energy-dispersive X-ray spectroscopy (EDXS) analysis in an aberration-corrected ThermoFisher Spectra 200 scanning transmission electron microscope. Samples were prepared using a dry deposition method. Briefly, the powdered catalyst was evenly dispersed onto a carbon-coated copper grid. Prior to loading into the microscope, the grids were plasma-cleaned for 5 min to eliminate surface contaminants and improve sample adhesion. ImageJ software was used to statistically analyze the particle size. For each sample, at least 100 particles were measured.

The surface composition of the catalysts was analyzed by X-ray photoelectron spectroscopy (XPS) on a Kratos Axis Ultra DLD spectrometer using monochromatic Al Kα radiation (hν = 1486.6 eV). Samples were mounted using double-sided adhesive tape, and binding energies were referenced to the C(1 s) binding energy of adventitious carbon contamination taken at 284.8 eV. The spectra were recorded using a pass energy of 160 eV for survey scans, while 40 eV was employed for detailed regional scans. CasaXPS v2.3.27 using a Shirley background and modified Wagner elemental sensitivity factors as supplied by the instrument manufacturer.

CO-DRIFTS was tested on Bruker Tensor 27 spectrometer and operated with OPUS software. The samples were loaded in a DRIFT Tensor 27 spectrometer and flushed with N_2_ for 30 min to record a baseline. The sample was then exposed to 10% CO/N_2_ until complete saturation of the Pd sites was achieved. The system was then flushed with pure N_2_ until there was no presence of atmospheric CO and then the spectra were recorded.

The thermal profiles of the different particles were measured by thermogravimetric analysis (TGA). The particles (∼10 mg in a 100-μL alumina crucible) were treated from 30 °C to 900 °C using a heating rate of 10 °C/min under airflow [30 mL(STP)/min].

The contact angle of the particles was measured by the Sessile drop method using a Dataphysics OCA 35 instrument by depositing a small drop (repeated three times per sample) of 5 μL of water on pellets formed from powder. The pellets were prepared using at least 200 mg of particle powder and then subjected to compression under a load of 3 tons using a press for 5 min. The shape of the drops was observed and used to measure the contact angle.

The zeta potential of particle dispersions in water was measured using a Malvern Zetasizer Nano ZS zeta potential analyzer at 25 °C. Around 800 μL of well-dispersed particle dispersions were transferred into a Malvern Folded Capillary Cell DTS1070. Each measurement was then passed in 100 runs. The repeatability for each dispersion was confirmed after 3 measurements.

RB-modified particles were visualized and scanned on a Zeiss LSM880 Airyscan Fast upright confocal microscope (Zeiss, Jena, Germany) using a 63x/1.4 plan-apochromat oil immersion objective. Appropriate excitation and detection parameters were used for sequential acquisition of red fluorescent signal from RB-modified silica particles (excitation maximum [ex max]: 546 nm; emission maximum [em max]: 567 nm) and green, fluorescent signal from aqueous FITC solution (ex max: 495 nm; em max: 519 nm). Confocal optical sections were taken through individual particles and their surrounding aqueous milieu to visually assess the spatial relationship between the red and green fluorescent signals. Images (16 bit) were presented as 2D or 3D red/green overlays. A line profile tool was used to generate a histogram of red/green, fluorescent intensity values across individual fluorescent particles in selected image fields. The histograms depict the total number of pixels at each fluorescent intensity value across the arbitrarily positioned line (i.e., distance in µm along horizontal axis and pixel intensity value along vertical axis).

The UV-Vis light absorption spectra of particle dispersion in water were measured on an Agilent Technologies Cary 60 UV-Vis monochromator spectrophotometer. The scanning wavelength range of UV-Vis light was set between 200 and 800 nm, whereas the scanning speed was set at medium speed. The baseline was measured by testing deionized water as reference solution.

### Density functional theory (DFT) calculations

The Vienna Ab-initio Simulation Package (VASP) version 6.4 software was employed throughout this work^45,46^. The revised PBE functional from Perdew, Burke, and Ernzerhof by Hammer and Hansen (RPBE) was used to calculate the exchange–correlation energy^47^. Projected-augmented wave (PAW) pseudopotentials were employed to describe the core electrons^48,49^. Dispersion corrections were included through Grimme’s dispersion correction scheme, DFT-D3^50^. The plane-wave kinetic cutoff was set to 550 eV, the electronic energy convergence threshold set to 1×10^–6^ eV, and the ionic convergence to 0.02 eV/Å. Electronic smearing around the Fermi level was described using the order-2 Methfessel-Paxton method in metal bulk and slabs, with a smearing parameter of 0.2 eV and a k-spacing of 0.2 pts.Å^-1^^51^. Metal slabs were separated by 15 vacuum layers along the c-axis, preventing interaction between periodic images (the vacuum layer was appropriately adjusted to accommodate the adsorbent at the surface). Molecules in the gas phase were described by a single gamma-centered k-point in a 15 × 15 × 15 Å vacuum box, using Gaussian smearing around the Fermi level with a smearing parameter of 0.05 eV. Frequencies were calculated via the finite-difference method implemented in VASP, with electronic energy convergence set to 1×10^-9 ^eV and ionic convergence to 1 × 10^–8 ^eV.

The models used to construct the training set for AuPd-alloy global optimization were built from a face-centered-cubic (fcc) *(4x4x4)* AuPd supercell with a fixed composition of Au_32_Pd_32_. A total of 49 initial cells were created with a lattice parameter calculated by below equation 

4 $$a=\frac{{a}_{{Pd}}^{{expt}}+{a}_{{Au}}^{{expt}}}{2}\times {{{\mathcal{U}}}}\left(\min=0.95,\max=1.05\right)$$

where ${a}_{{Pd}}^{{expt}}=3.890{{{{\AA }}}}$, ${a}_{{Au}}^{{expt}}=4.078{{{{\AA }}}}$ represents the experimental lattice constant of each metal^52,53^, and ${{{\mathcal{U}}}}\left(\min=0.95,\max=1.05\right)$ induces a $\pm 5\%$ perturbation on the lattice. The resulting cells were relaxed by optimising atomic positions and cell parameters. In addition, a set of optimised structures was selected, and Gaussian noise was injected in atomic positions before undergoing another round of ionic relaxation. The final dataset contained 950 DFT single points.

The atomic environment was described by combining atom-centered symmetry functions (ACSF-G2–2 bodies) with smooth overlap of atomic positions (SOAP, many-bodies) descriptors^54–56^. The hyperparameters for ACSF-G2 were identical for both metal species: $\eta \in \{0.00,\,0.01,\,0.07,\,0.10,\,0.50\}\,[{{{{\AA }}}}^{-2}]$, ${R}_{s}\in \left\{0.00,\,1.50,\,3.00,\,4.50\right\}\,[{{{{\AA }}}}]$, while for SOAP descriptors, the radial degree was set to $n\in \left\{1,\,2,\,3\right\}\,[\varnothing ]$, and the angular moment of the spherical harmonics used for angular description was set to $l\in \{1,\,2,\,3,\,4\}$. Let k represent the number of elements present in the dataset (k=2), and ${{{\rm{card}}}}(x)$ represent the number of elements in the set x, the dimension of the fingerprint describing each atom in the system is given by below equation 

5 $$\dim \left({{{{\mathcal{F}}}}}_{i}\right)=k\times {{{\rm{card}}}}\left(\eta \right)\times {{{\rm{card}}}}\left({R}_{s}\right)+\frac{k(k+1)}{2}\times \frac{{{{\rm{card}}}}\left(n\right)({{{\rm{card}}}}\left(n\right)+1)}{2}\times {{{\rm{card}}}}\left(l\right)$$

which gives $$\dim \left({{{{\mathcal{F}}}}}_{i}\right)=112$$.

The Behler-Parinello high-dimensional neural network (HDNN) trained had a structure of (112-25-20-1)^54^, i.e., two hidden layers containing 25 and 20 neurons, respectively. The hyperbolic-tangent activation function was used, and the input data was normalized prior to the training via below equation 

6 $${{{{\mathcal{F}}}}}_{i}^{(\,\,j){\prime} }=\frac{{{{{\mathcal{F}}}}}_{i}^{(\,\,j)}-\left\langle {{{\boldsymbol{{{\mathscr{F}}}}}}}^{(\,\,{{{\boldsymbol{j}}}})}\right\rangle }{{{{\rm{std}}}}\left({{{\boldsymbol{{{\mathscr{F}}}}}}}^{(\,\,{{{\boldsymbol{j}}}})}\right)}$$

setting the mean value of the j-th element of the fingerprint to zero across the whole dataset, and its standard deviation to 1.0. The model was trained for 300 epochs with an exponential learning rate decay starting at 5e^–4^, decaying by a factor of 0.99 every 5 epochs. The trained model achieves root-mean-squared errors (RMSEs) of 3 meV/atom and 0.094 eV/Å on the test set.

Global optimization of the fcc-AuPd alloy was carried out at constant volume and composition (Au_32_Pd_32_) using a Monte Carlo selection algorithm. An initial pool of structures is generated by constructing a set of Au_32_Pd_32_ structures at constant volume obtained during the construction of the training set. At each iteration, a worker selects a structure from its own pool using a Monte Carlo trial. The energy of the candidate, ${E}_{i}$, is compared to that of the current most stable structure in the pool, ${E}_{0}$, via Eq. 7

7 $$p={e}^{\frac{-\Delta E}{{k}_{B}T}},\Delta E={E}_{i}-{E}_{0}$$

where the simulation temperature, T is set to 300 K and ${k}_{B}$ represent Boltzmann’s constant (8.6173e^–5^ eV.K^–1^). After determining p, a value $\sigma \sim {{{\mathcal{U}}}}({{ \min }=0.0,{ \max }=1.0})$ is sampled. If $\sigma \le p$, the structure is accepted and undergoes a global optimisation (GO) step. Otherwise, a new structure is picked and evaluated until the condition $\sigma \le p$ is met. This procedure ensures the algorithm is biased toward selecting low-laying structures. Upon selection, one of the four GO operators is applied to the candidate:

1. *Pair-mutation*; two atoms are selected, subject to the constraint that they must have two different chemical elements associated and are swapped,
2. *Cluster-shuffle*; An atom is randomly selected in the structure, and all atoms within a given cut-off radius around this atom are shuffled,
3. *Block-shuffle*; A fraction of the supercell is randomly selected, and all chemical elements of selected atoms are randomly shuffled,
4. *Full shuffle*; Completely reshuffle the supercell.

The different hyperparameters associated with each operator and the values selected in the present work are described in Table 1. Upon application of the selected GO operator, the candidate undergoes local optimization using the trained MLFF, and the resulting energy is compared to the highest energy in the pool. If ${E}_{i} < {E}_{\max }$, the structure of highest energy is deleted from the pool and replaced by the new candidate. Otherwise, the candidate is discarded.

**Table 1:** Hyperparameters of the global optimization operators used in the present work

Operator (probability)	Hyperparameters	Hyperparameter sampling
Pair-mutation (40%)	None	None
Cluster-shuffle (25%)	Cut-off radius (R_cut_)	${{{{\rm{R}}}}}_{{{{\rm{cut}}}}}=3.00{{{{\AA }}}}$
Block-shuffle (25%)	Cell fraction (x)	${{{\rm{x}}}}= \max \left(3,\,{{\mathrm{int}}}\left({{{\rm{\alpha }}}}{{{\rm{N}}}}\right)\right),\,{{{\rm{\alpha }}}} \sim {{{\mathscr{U}}}}\left(0.0,\,0.2\right)$
Full shuffle (10%)	None	None

N represents the number of atoms present in the supercell

### Computer fluid dynamics (CFD) simulations

2D numerical simulation of plastron formation in an air-water system (density ratio: 1/1000 kg/m^3^; kinematic viscosity: 1.148 × 10^–5^ m^2^/s for air and 1 × 10^–6^ m^2^/s for water; interfacial tension: 0.072 N/m) was performed using the Volume of Fluid (VOF) method in the open-source CFD software OpenFOAM-v1906. The solver used was interFoam, which is designed to track the interface between two incompressible, immiscible fluids without considering heat transfer.

In interFoam, the simulation begins by solving a scalar transport equation for the volume fraction of the continuous phase, based on the initial and boundary conditions. From the resulting volume fraction field, the interface curvature was computed and used to evaluate the surface tension force according to Brackbill’s Continuum Surface Force (CSF) model^57^. The surface tension force was then included in the momentum equation. Finally, the momentum equation was solved to obtain the velocity and pressure fields. To accurately represent the multiphase nature of the flow, an additional compression term involving the relative velocity between the phases was added to the volume fraction transport equation.

#### Governing equations

The following equations were solved in this study to simulate gas plastron formation. The mass and momentum conservation equations (Eqs.( 8)–(9)) were solved along with the scalar transport equation for the volume fraction as given in Eq. 10

8 $$\nabla .{{{\bf{U}}}}=0$$

9 $$\frac{\partial {{{\rm{\rho }}}}{{{\bf{U}}}}}{\partial {{{\rm{t}}}}}+\nabla .({{{\rm{\rho }}}}{{{\bf{U}}}}.{{{\bf{U}}}})=-{\nabla {{{\rm{p}}}}}^{*}+\nabla .{{{\boldsymbol{\tau }}}}+{{{{\bf{f}}}}}_{{{{\bf{b}}}}}$$

10 $$\frac{\partial {{{{\rm{\alpha }}}}}_{{{{\rm{cont}}}}}}{\partial {{{\rm{t}}}}}+\nabla .({{{{\rm{\alpha }}}}}_{{{{\rm{cont}}}}}{{{\bf{U}}}})+\nabla .({{{{\rm{\alpha }}}}}_{{{{\rm{cont}}}}}*(1-{{{{\rm{\alpha }}}}}_{{{{\rm{cont}}}}})*{{{{\bf{U}}}}}_{{{{\bf{r}}}}})=0$$

In Eq.( 5), ${{{\rm{\rho }}}}$ is the density of the effective phase, U is the velocity vector, ${{{\boldsymbol{\tau }}}}$ is the shear stress tensor, ${a}_{{{{\rm{cont}}}}}$ is the volume fraction of the continuous phase, ${{{{\bf{U}}}}}_{{{{\bf{r}}}}}$ is the relative velocity vector, and ${{{{\bf{f}}}}}_{{{{\bf{b}}}}}$ is the body force per unit mass which includes gravity and surface tension forces as given in Eq. 11

11 $${{{{\bf{f}}}}}_{{{{\bf{b}}}}}={{{\bf{g}}}}.{{{\bf{x}}}}\nabla {{{\rm{\rho }}}}+{{{\bf{f}}}}$$

where $\nabla {{{{\rm{p}}}}}^{*}$ is the modified pressure obtained by subtracting the hydrostatic pressure (ρ**gx**) from the total pressure, **g** is the gravitational acceleration vector and **x** is the position vector, and **f** is the volumetric surface tension force that was computed from the continuum surface force model as given in Eq. 12

12 $${{{\bf{f}}}}={{{\rm{\sigma }}}}*{{{\boldsymbol{\kappa }}}}*\nabla {{{{\rm{\alpha }}}}}_{{{{\rm{cont}}}}}$$

where σ is the surface tension and **κ** is the surface curvature that was calculated by Eq. 13

13 $${{{\boldsymbol{\kappa }}}}=-\nabla .\widehat{{{{\bf{n}}}}}$$

The unit normal vector $\widehat{{{{\bf{n}}}}}$ was defined as

14 $$\widehat{{{{\bf{n}}}}}=\frac{\nabla {{{{\rm{\alpha }}}}}_{{{{\rm{cont}}}}}}{|\nabla {{{{\rm{\alpha }}}}}_{{{{\rm{cont}}}}}|}$$

Near the particle surface, **∇α** is asymmetric and hence should be modified as given in Eq. 15

15 $$\widehat{{{{\bf{n}}}}}=\cos {{{\rm{\theta }}}}.\widehat{{{{{\bf{n}}}}}_{{{{\bf{wall}}}}}}+\sin {{{\rm{\theta }}}}.{{{\bf{t}}}}$$

where **t** is the tangential interface direction calculated from the neighboring cell volume fraction values.

The final expression of the curvature (**κ**) becomes

16 $${{{\boldsymbol{\kappa }}}}=-{{{\boldsymbol{\nabla }}}}.(\cos {{{\rm{\theta }}}}.\widehat{{{{{\bf{n}}}}}_{{{{\bf{wall}}}}}}+\sin {{{\rm{\theta }}}}.{{{\bf{t}}}})$$

where θ is the contact angle between the particle surface and the effective phase in the domain, and $\widehat{{{{{\bf{n}}}}}_{{{{\bf{wall}}}}}}$ is the unit normal vector at the particle surface.

In Eq.( 6), the relative velocity, **U**_**r**_ was calculated as given in Eq. 17

17 $${{{{\bf{U}}}}}_{{{{\bf{r}}}}}=\frac{{{{{\rm{C}}}}}_{{{{\rm{\alpha }}}}}*|{{{\bf{U}}}}|*\nabla {{{{\rm{\alpha }}}}}_{{{{\rm{cont}}}}}}{|\nabla {{{{\rm{\alpha }}}}}_{{{{\rm{cont}}}}}|}$$

where C_*a*_ is the artificial compression term supplied by the user.

The density and viscosity of the effective phase was calculated as the summation of individual densities and viscosities of the phases weighted by their respective volume fractions as given in Eqs. 18 and 19

18 $${{{\rm{\rho }}}}={{{{\rm{\rho }}}}}_{{{{\rm{cont}}}}.}*{{{{\rm{\alpha }}}}}_{{{{\rm{cont}}}}.}+{{{{\rm{\rho }}}}}_{{{{\rm{disp}}}}.}*(1-{{{{\rm{\alpha }}}}}_{{{{\rm{cont}}}}.})$$

19 $${{{\rm{\mu }}}}={{{{\rm{\mu }}}}}_{{{{\rm{cont}}}}.}*{{{{\rm{\alpha }}}}}_{{{{\rm{cont}}}}.}+{{{{\rm{\mu }}}}}_{{{{\rm{disp}}}}.}*(1-{{{{\rm{\alpha }}}}}_{{{{\rm{cont}}}}.})$$

The multidimensional universal limiter for explicit solution (MULES) algorithm was used to keep the *a* values between 0 and 1. The velocity and pressure fields were obtained by employing the PIMPLE algorithm which is the combination of the PISO (pressure implicit with splitting of operators) and SIMPLE (semi-implicit method for pressure linked equations) to ensure a flexible time stepping along with the improved convergence and stability for multiphase simulations. More details can be found in a previous publication^58^.

#### Computational domain and boundary conditions

The computational domain consisted of a rectangular microchannel (300 * 600 * 10 μm) with approximately 43,000 cells resulting in a hexahedral grid size of 2 μm. A 100-μm cylindrical hole was created at 300 μm height beside which a bubble of 30 μm diameter was placed. The computational domain with the boundaries and the mesh is given in Supplementary Fig. 6a, b. The boundary condition for the volume fraction field was set to zeroGradient at the channel walls, inletOutlet at the top and constant AlphaContactAngle at the particle surface. For the velocity field, noSlip boundary condition was set at the channel walls and the particle surface and pressureInletOuletVelocity boundary condition was set at the top. For the pressure field, a fixedFluxPressure boundary condition was set at the channel walls and particle surface, and a totalPressure boundary condition was set for the top. More details about these boundary conditions can be found in a previous study^45^.

The Courant number (Co) was used as descriptor to reach stable solution. It is defined as the ratio of the fluid velocity to the numerical speed with which the fluid crosses one cell in one time step as follows:

20 $${{{\rm{Co}}}}=\frac{|{{{\bf{U}}}}|\Delta t}{\Delta x}$$

where Δx and Δt are the time and position step, respectively.

An adjustable time stepping method was adopted to automatically modify the time step for faster convergence. Due to the small grid size, a time stepping of 10^–8 ^s was chosen (Courant number ∼0.2) to reach a stable solution.

## Supplementary information


Supplementary Information
Description of Additional Supplementary Files
Supplementary Movie 1
Supplementary Movie 2
Transparent Peer Review file


## Source data


Source Data


## Data Availability

All data are available in the main text and supplementary materials. Source data are provided with this paper and in Figshare with https://figshare.com/s/306b9b5111baac75502f. Data are available from the corresponding authors upon request. Source data are provided with this paper.
