# Supplementary material for: Plastron-mediated direct H2O2 synthesis
Source: Nat Commun. 2026 Aug 4;17:9389. doi: 10.1038/s41467-026-76198-9 (PMC13534698; doi:10.1038/s41467-026-76198-9)
Supplement: Supplementary file 1 [file 41467_2026_76198_MOESM1_ESM.pdf]

Supplementary Information

Plastron-Mediated Direct H_2O_2 Synthesis

Kang Wang,[a] Vivekananda Sinha,[a] Anthony J. Hayes,[b] Alexandre Boucher,[a] Alberto Roldan,[a] David J. Morgan,[c] Richard J. Lewis,[c] Graham J. Hutchings,[c] Marc Pera-Titus[a,*]

a) Cardiff Catalysis Institute, School of Chemistry, Cardiff University; Translational Research Hub, Maindy Road, Cardiff, CF24 4HQ, UK.

b) Cardiff School of Biosciences, Cardiff University, Cardiff, CF10 3AX, UK.

c) Max Planck-Cardiff Centre on the Fundamentals of Heterogeneous Catalysis FUNCAT, Cardiff Catalysis Institute, School of Chemistry, Cardiff University; Translational Research Hub, Maindy Road, Cardiff, CF24 4HQ, UK.

Table of contents

Section S1: Supplementary Methods.

Supplementary Figure 1. Diagrammatic representation of catalyst preparation. a, sol-immobilization method used for catalyst preparation. **b**, surface modification of SiO_2. **c**, surface modification of AuPd alloy nanoparticles. **d**, Schematic representation of reactor set-up for the direct synthesis of H_2O_2. **e**, Schematic illustration of a hydrophobic, catalytic particle with a gas plastron surrounding it.

Supplementary Figure 2. Representative STEM-HAADF images and EDS spectra of catalysts (gold, palladium), and particle size distributions. a, AuPd/SiO_2; **b**, SH-AuPd/SiO_2; **c**, SH-AuPd/SiO_2-F-60µL particles; **d**, spent SH-AuPd/SiO_2-F-60µL particles. Conditions: Samples were prepared using a dry deposition method onto a carbon-coated copper grid, and the grids were plasma-cleaned for 2 min. Sulfur was not detected due to electron-beam induced damage of the samples.[1]

Supplementary Figure 3. XPS and CO-DRIFTS data. High-resolution narrow scan of AuPd/SiO_2, SH-AuPd/SiO_2, SH-AuPd/SiO_2-F-30µL, SH-AuPd/SiO_2-F-60µL, and SH-AuPd/SiO_2-F-120µL for **a**, S(2p); **b**, C(1s), the carbon numbering is shown in fig. **d**; and **c**, F(1s); **d**, The molecular structure and labeled carbon of butanethiol and 1H,1H,2H,2H-perfluorooctyltriethoxysilane **e**, CO-DRIFTS spectra of pristine SiO_2, AuPd/SiO_2, SH-AuPd/SiO_2, AuPd/SiO_2-F-60µL, SH-AuPd/SiO_2-F-60µL. **f**, CO-DRIFTS spectra of spent SH-AuPd/SiO_2-F-60µL. High-resolution narrow scan of the thrice-used SH-AuPd/SiO_2-F-60µL for **g**, Au(4d) and Pd(3d); **h**, S 2p, **i**, C(1s), the carbon numbering is shown in fig. **d**. The weak interaction between CO and the Au surface in **e** leads to only faint CO-Au vibrational features.[2]

Supplementary Figure 4. Contact angle of water with pellets based on **a**, SH-AuPd/SiO_2; **b**, SH-AuPd/SiO_2-F-15 µL;**c,** SH-AuPd/SiO_2-F-30 µL; **d**, SH-AuPd/SiO_2-F-45 µL; **e**, SH-AuPd/SiO_2-F-60 µL; **f**, SH-AuPd/SiO_2-F-120 µL; **g**, SH-AuPd/SiO_2-C-60 µL; and **h**, spent SH-AuPd/SiO_2-C-60 µL. Contact angle for **i**, pure water; **j**, 20% methanol in water; **k**, 50% methanol in water; and **l**, 100% methanol with SH-AuPd/SiO_2-F-60 µL pellet. Contact angle for **m**, pure water; **n**, 20% isopropanol in water; **o**, 50% isopropanol in water; and **p**, 100% isopropanol with SH-AuPd/SiO_2-F-60 µL pellet.

Supplementary Figure 5. Butanethiol adsorption on AuPd(111). Optimized geometry of butanethiol adsorbed on the AuPd(111) surface and calculated IR-spectra for butanethiol@AuPd (black) compared to gas-phase butanethiol (blue).

Supplementary Figure 6. H_2O_2 adsorption on AuPd(111). Optimized geometry of H_2O_2 adsorbed on the AuPd(111) surface and calculated IR-spectra for H_2O_2@AuPd(111) (black) compared to gas-phase H_2O_2 (blue).

Supplementary Figure 7. H_2O_2/butanethiol co-adsorption on AuPd(111). Optimized geometry of co-adsorbed H_2O_2/butanethiol on the AuPd(111) surface and calculated IR-spectra for gas-phase H_2O_2 (blue) and adsorbed butanethiol@AuPd(111) (red).

Supplementary Figure 8. O_2/butanethiol and H_2O co-adsorption on AuPd(111). Optimized geometry of (a) O_2 and (b) H_2O co-adsorbed with butanethiol on AuPd(111), together with calculated IR-spectra of the co-adsorbed systems, isolated gas-phase molecules, and adsorbed butanethiol@AuPd(111).

Supplementary Figure 9. Turbidity and zeta potential data. Optical images showing gas release from **a**, SH-AuPd/SiO_2-F-30μL; and **b**, SH-AuPd/SiO_2-F-120μL particles dispersed in water. Experimental conditions: 4% H_2/air, 3 bar, saturated for 20 min at 22 °C. The pressure was released at 0 s. **c**, c1,SH-AuPd/SiO_2-F-60μL particles aqueous dispersion. c2, Dropping methanol into SH-AuPd/SiO_2-F-60μL particles dispersion. c3, SH-AuPd/SiO_2 particles aqueous dispersion. c4, Dropping methanol into SH-AuPd/SiO_2 particles dispersion. Experimental conditions: ambient pressure, 22 °C, 100 μL methanol. **d**, Optical image of pure water in a cuvette, and SH-AuPd/SiO_2, SH-AuPd/SiO_2-F-60μL, and SH-AuPd/SiO_2-F-120μL particles dispersed in water in a cuvette. Conditions: 2.5 mg particles dispersed in 5 mL of water. **e**, Zeta potential of SH-AuPd/SiO_2, SH-AuPd/SiO_2-F-60μL, and Spent SH-AuPd/SiO_2-F-60μL dispersions in water. **f**, Correlation between the catalytic activity, plastron stability, and hydrophobicity of different particles. Particles: 16°: SH-AuPd/SiO_2, 61°: SH-AuPd/SiO_2-F-15μL, 110°: SH-AuPd/SiO_2-F-30μL, 117°: SH-AuPd/SiO_2-F-45μL, 125°: SH-AuPd/SiO_2-F-60μL, 137°: SH-AuPd/SiO_2-F-120μL. **g**, Time-evolution of absorbance of different hydrophobicity particle dispersions. Particles are SH-AuPd/SiO_2, SH-AuPd/SiO_2-F-15μL, SH-AuPd/SiO_2-F-30μL, SH-AuPd/SiO_2-F-45μL, SH-AuPd/SiO_2-F-60μL, and SH-AuPd/SiO_2-F-120μL **h**, Plastron stability of the SH-AuPd/SiO_2-F-60μL particle. **i**, Particle size distribution of SH-AuPd/SiO_2-F-60μL and Spent SH-AuPd/SiO_2-F-60μL. **j**, ^{1}H NMR spectrum of the filtrate after solution. Experimental conditions: 10 ppm CH_3CN was used as the internal standard.

Supplementary Figure 10. Volume of Fluid simulation results. a, Computational domain with boundaries and **b**, hexahedral grid elements for calculations with the VoF method. **c**, Plastron stability for a hydrophobic particle at 0.015 s, 0.02 s, 0.025 s, 0.035 s. **d**, Streamline analysis after bubble attachment on a hydrophobic particle at 0.035 s.

Supplementary Figure 11. Control experiments and catalyst reusability. a, Evolution of the H_2O_2 concentration for SH-AuPd/SiO_2-F-60μL particles at variable loading. Reaction conditions for H_2O_2 production: 5 mL of deionized water, H_2:Air=4%, 3 bar, 22 °C, 1500 rpm, 30 min. Reaction conditions for H_2O_2 degradation: 5 mL 2500 μM H_2O_2 solution, H_2:N_2=4%, 3 bar, 22 °C, 1500 rpm, 30 min, **b**, H_2O_2 synthesis and degradation activity of SiO_2-F and SiO_2 particles (circles, synthesis; squares, degradation). Reaction conditions for H_2O_2 synthesis: 2.5 mg particles, 5 mL H_2O, 3 bar H_2:Air = 4%, 22 °C, 1500 rpm, 30 min. Reaction conditions for H_2O_2 degradation: 2.5 mg particles, 5 mL of H_2O_2 (2500 μM), 3 bar H_2:N_2 = 4%, 22 °C, 1500 rpm, 30 min. **c**, H_2 conversion and H_2O_2 selectivity for SH-AuPd/SiO_2-F-60μL particles combined with noncatalytic particles. Reaction conditions: 2.5 mg of SH-AuPd/SiO_2-F-60μL combined with various amounts (2.5, 5, 10,100 mg) of noncatalytic SiO_2-F or SiO_2 particles, 5 mL H_2O, 3 bar H_2:Air = 4%, 22 °C, 1500 rpm, 30 min. **d**, H_2O_2 concentration in isopropanol/water mixtures at variable particle wettability. Reaction conditions: 5 mL of isopropanol/water mixture, H_2:Air=4%, 3 bar, 22 °C, 1500 rpm, 30 min. **e**, Kinetic plots of $-Ln(C_{H2O2})$ against time for the H_2O_2 direct synthesis catalyzed by SH-AuPd/SiO_2-F-60μL with plastron. **f**, Kinetic plots of $-Ln(C_{H2O2})$ against time for the H_2O_2 direct synthesis catalyzed by SH-AuPd/SiO_2 without plastron. **g**, Variation of TOF at time t=0 with reciprocal temperature for SH-AuPd/SiO_2-F-60μL and SH-AuPd/SiO_2. **h**, Conversion of H_2 and Selectivity of H_2O_2 for SH-AuPd/SiO_2, SH-AuPd/SiO_2-F-60μL, and thrice-used SH-AuPd/SiO_2-F-60μL particles. Reaction conditions: 2.5 mg particles, 5 mL of deionized water, H_2:Air=4%, 3 bar, 22 °C, 1500 rpm, 30 min. **i**, Evolution of the H_2O_2 concentration for AuPd/TiO_2 and SH-AuPd/SiO_2-F-60μL particles. Reaction conditions: 2.5 mg of catalyst, 5 mL of deionized water, 3 bar H_2:Air = 4%, 22 °C, 1500 rpm, 30 min.

Supplementary Table 1. Main properties of catalytic particles.

Supplementary Table 2. Metal leaching tests.

Supplementary Table 3. Catalytic activity comparisons against state-of-the-art catalysts for direct synthesis of H_2O_2.

Section S1. Supplementary Methods

S1.1. Materials

Commercial silica particles (Aerosil® 200), ethanol (99%), palladium(II) chloride (98%), gold(III) chloride (~50% Au basis), sodium borohydride ($NaBH_4$, 98%), poly(vinyl alcohol) (PVA, 80% hydrolyzed), sulfuric acid (95%-98%), potassium tetraoxalate dihydrate (98%), 3-aminopropyltriethoxysilane (APTES, 98%), rhodamine B isothiocyanate, mixed isomers (RBITC), fluorescein isothiocyanate (FITC, 98%), octyltriethoxysilane (OTES, 97%), p-toluenesulfonic acid monohydrate (PTSA, 98%), and hydrogen peroxide (H_2O_2, 50 wt.% in water) were purchased from Sigma-Aldrich. 1H,1H,2H,2H-perfluorooctyltriethoxysilane (PFOTES, 95%) was purchased from Fluorochem. All the chemicals were used as received without further purification.

S1.2. Catalyst leaching tests

After completion of the reactions, the catalysts recovered from different batches were collected via centrifugation, washed three times with acetone, and subsequently dried overnight at 25 °C in a vacuum oven. The recovered catalysts were then analyzed using Microwave Plasma-Atomic Emission Spectroscopy (MP-AES). The solution after reaction was analyzed by inductively coupled plasma mass spectrometry (ICP-MS) to assess possible Au and Pd leaching.

S1.3. H_2O_2 degradation tests

A total of 2.5 mg of each catalyst was dispersed in 5 mL aqueous H_2O_2 solution with an initial concentration of 2500 μM at 22 °C. The headspace of the tube was filled with 3 bar air. The mixture was stirred at 1500 rpm for different times, after which the residual H_2O_2 concentration was measured using UV-Vis method as stated above. The hydrogenation tests were carried out under the same conditions, but the gas atmosphere in the headspace was switched to 3 bar 4% H_2/N_2. Control experiments indicate that both SiO_2-F and SiO_2 particles display no catalytic activity for H_2O_2 formation and H_2O_2 degradation.

S1.4. Catalyst recycling tests

100 mg SH-AuPd/SiO_2-F-60μL was used for H_2O_2 direct synthesis. After 30 min, the catalyst was separated by centrifugation, washed with acetone, and dried under vacuum at 25 °C. Subsequently, 80 mg of once-used SH-AuPd/SiO_2-F-60μL catalyst was used to run another 30 min reaction and recycle it. Following this, 50 mg of twice-used SH-AuPd/SiO_2-F-60μL catalyst was used for the re-doping step as stated below. 2.5 mg of each spent SH-AuPd/SiO_2-F-60μL catalyst was used for the reusability tests.

Re-doping butanethiol monolayers of spent SH-AuPd/SiO_2-F-60μL particles: The 50 mg twice-used SH-AuPd/SiO_2-F-60μL catalysts were dispersed into 3 mL of ethanol, and 2 μL of butanethiol (similar concentration to preparing fresh catalyst) was added. This dispersion was stirred at 22 °C for 4 hours. After the re-doping, the dispersion was centrifuged and washed with ethanol, and the pellet was dried at 25 °C under vacuum.

S1.5. Calculation of gas film thickness in plastrons

Assuming spherical particle geometry and applying a force balance between gravity and buoyancy (**Supplementary Figure 1e**), the average gas film (plastron) thickness on this catalyst is estimated as below. Let's consider a hydrophobic, spherical particle (d=200 nm, density = 2.2 g/cm^3) with a pre-formed plastron suspended in water by

assuming that the gravity force on the particle equals the buoyancy force. We can then write:

$$\frac{\pi d_p{}^3}{6} g\rho_p = \frac{\pi}{6}\left(d_p + 2d_0\right)^3 g\rho_w \; + \; 3\pi\mu_w U d_p \tag{S1}$$

where d_w and μ_w are the water density and viscosity, respectively, g is the acceleration of gravity (9.81 m/s^2), ρ_p and d_p are the particle density and diameter, respectively, d_0 is the gas film thickness, and U is the settling velocity.

Assuming that U ~ 0, eq S1 can be rewritten as follows

$$\frac{\pi}{6}\left(d_p + 2d_0\right)^3 g\rho_w = \frac{\pi}{6} d_p{}^3 g\rho_p \tag{S2}$$

Eq S2 can be further simplified to eq S3

$$\left(d_p + 2d_0\right)^3 \rho_w = d_p{}^3 \rho_p \tag{S3}$$

Taking into account the silica and water densities (2.2 and 1.0 g/cm^3 at 25 °C), eq S3 can be rewritten as follows

$$\frac{\rho_p}{\rho_w} = \left(\frac{d_p + 2d_0}{d_p}\right)^3 = \frac{2.2}{1.0} = 2.2 \tag{S4}$$

From eq S4 we can compute the gas film thickness:

$$\frac{d_p + 2d_0}{d_p} = 1.3 \tag{S5}$$

$$d_p + 2d_0 = 1.3d_p \tag{S6}$$

$$d_0 = 0.15d_p \rightarrow \mathbf{d_0 = 30\ nm} \tag{S7}$$

S1.6. Calculation of the frequency of gas regeneration in the plastrons

The volume of the gas film in the plastron was computed by eq S8

$$\mathrm{V(plastron) = V(particle + plastron) - V(particle)} \tag{S8}$$

The number of organosilica particles was computed by eq S9

$$\mathrm{No.\,particles} = \frac{\mathrm{m(total)}}{\mathrm{m(particle)}} \tag{S9}$$

The gas film volume in the plastrons was computed by eq S10:

$$\mathrm{V(total\ plastron) = No.\,(particle) * V(plastron)} \tag{S10}$$

The gas contains 4% H_2 and 20% O_2. Assuming that all the H_2 contained in each plastron is consumed during the reaction, the H_2 volume in the plastrons, $V(H_2)$, can be computed using the expression

$$V(H_2) = V(\text{total plastron}) * 4\% \tag{S11}$$

H_2O_2 is generated by all the H_2 and O_2 contained in the plastrons:

$$n(H_2O_2) = n(H_2) = \frac{PV(H_2)}{RT} \tag{S12}$$

where P is the gas pressure (3 bar), T is the temperature (298.15 K) and R is the gas constant (8.314 $J \cdot mol^{-1} \cdot K^{-1}$).

Based on the experimental results, H_2O_2 generated with minimal decomposition or hydrogenation. Taking into account the reaction time (30 min), the gas plastron regenerates 704 times during the reaction. From this value, the frequency of gas regeneration in the plastron can be computed as follows:

$$\textbf{Frequency} = \frac{704}{1800\ s} = 0.39\ s^{-1} \tag{S13}$$

S1.7. Characterization of product and gas. The mixture of the reaction solution and potassium titanyl oxalate acid solution was also analyzed using an Agilent Technologies Cary 60 UV-Vis monochromator spectrophotometer. The UV-Vis spectra were recorded over a wavelength range of 200-800 nm at a medium scanning speed. The baseline was established using a 1:1 mixture of deionized water and potassium titanyl oxalate acid solution.

Supplementary Figure 1. Diagrammatic representation of catalyst preparation. a, sol-immobilization method used for catalyst preparation. **b**, surface modification of SiO_2. **c**, surface modification of AuPd alloy nanoparticles. **d**, Schematic representation of reactor set-up for the direct synthesis of H_2O_2. **e**, Schematic illustration of a hydrophobic, catalytic particle with a gas plastron surrounding it.

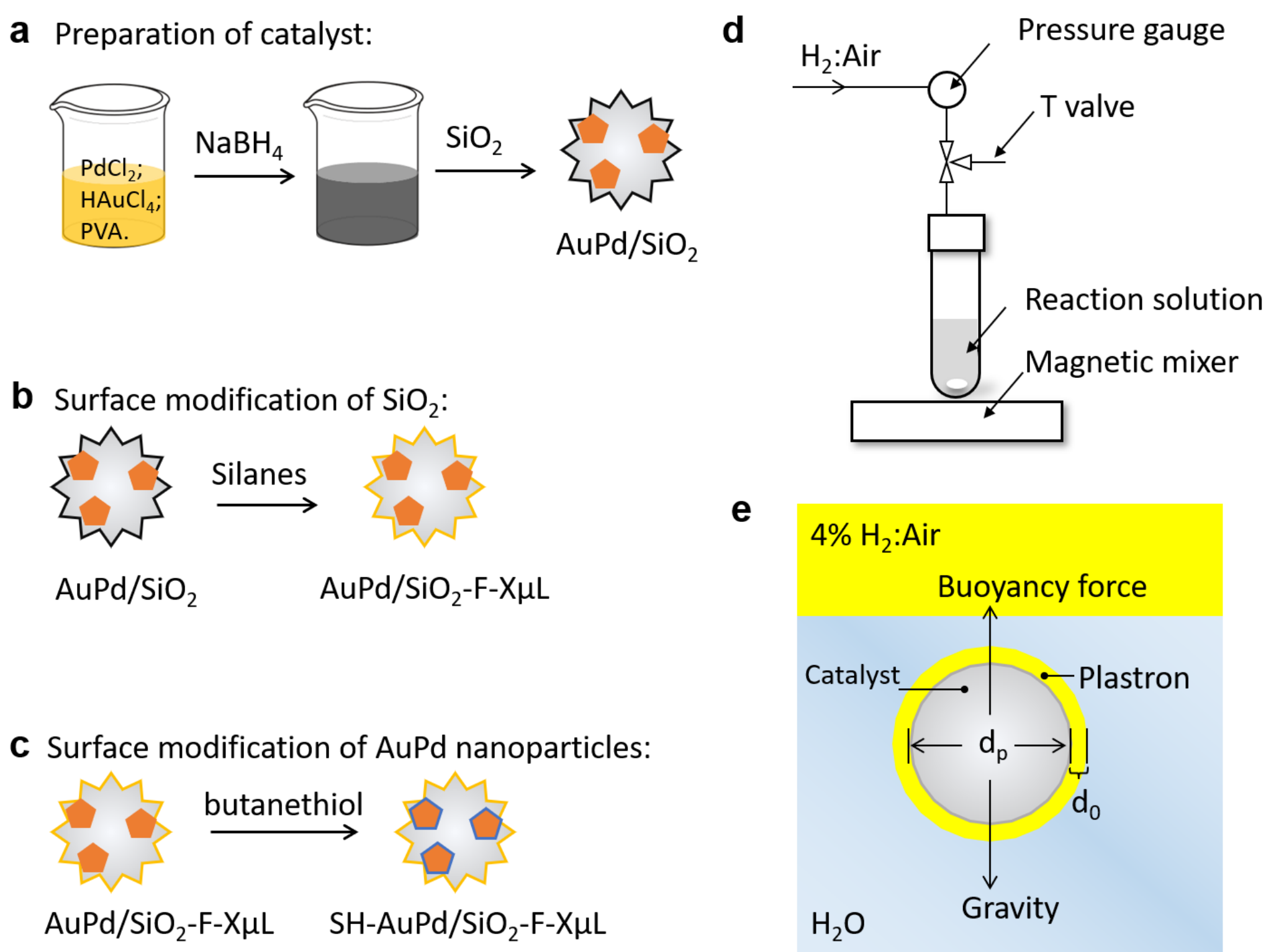

Supplementary Figure 2. Representative STEM-HAADF images and EDS spectra of catalysts (gold, palladium), and corresponding particle size distributions. **a**, AuPd/SiO_2; **b**, SH-AuPd/SiO_2; **c**, SH-AuPd/SiO_2-F-60μL particles; **d**, spent SH-AuPd/SiO_2-F-60μL particles. Conditions: Samples were prepared using a dry deposition method onto a carbon-coated copper grid, and the grids were plasma-cleaned for 2 min. Sulfur was not detected due to electron-beam induced damage of the samples.[1]

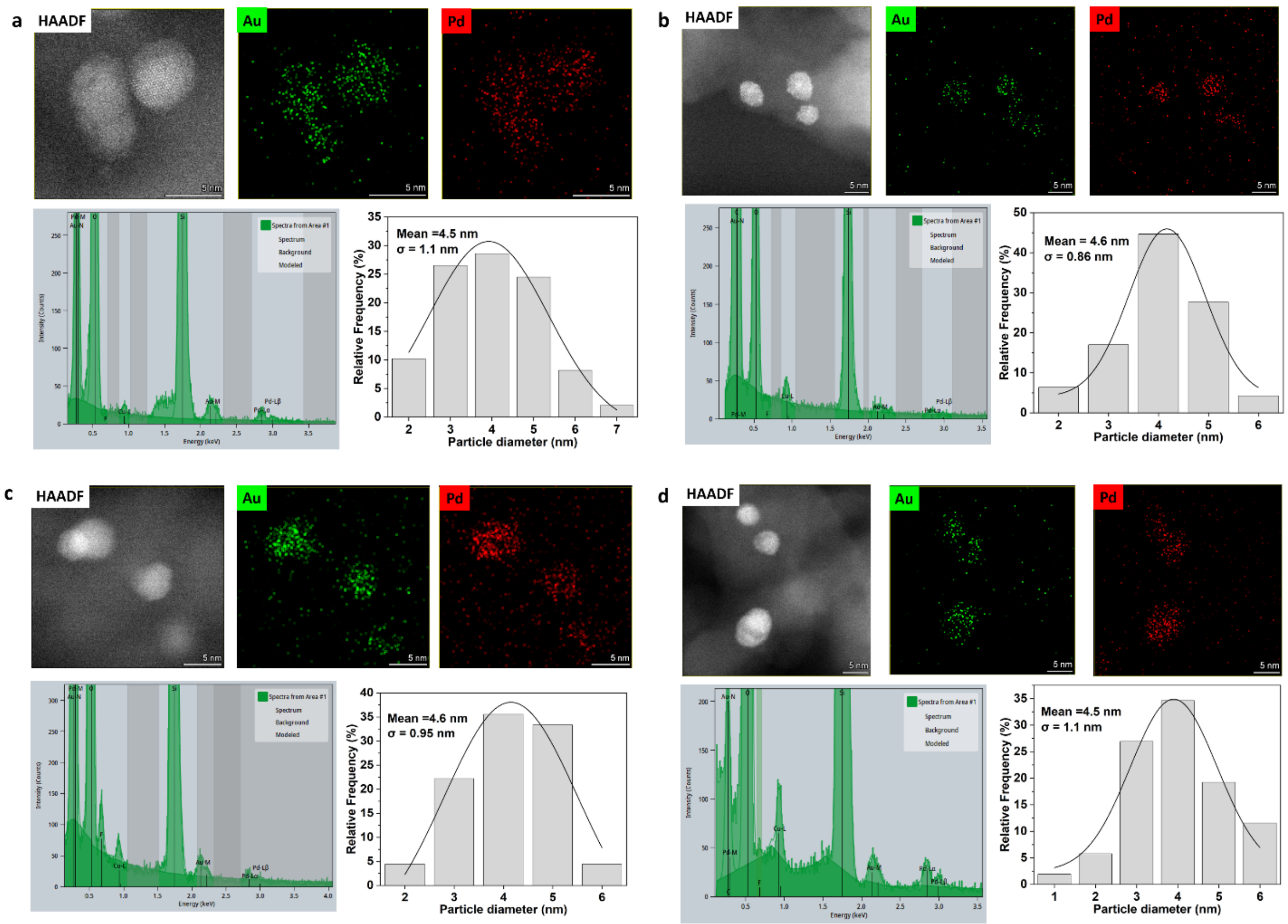

Supplementary Figure 3. XPS and CO-DRIFTS data. High-resolution narrow scan of AuPd/SiO_2, SH-AuPd/SiO_2, SH-AuPd/SiO_2-F-30μL, SH-AuPd/SiO_2-F-60μL, and SH-AuPd/SiO_2-F-120μL for **a**, S(2p); **b**, C(1s), the carbon numbering is shown in fig. **d**; and **c**, F(1s); **d**, The molecular structure and labeled carbon of butanethiol and 1H,1H,2H,2H-perfluorooctyltriethoxysilane **e**, CO-DRIFTS spectra of pristine SiO_2, AuPd/SiO_2, SH-AuPd/SiO_2, AuPd/SiO_2-F-60μL, SH-AuPd/SiO_2-F-60μL. **f**, CO-DRIFTS spectra of spent SH-AuPd/SiO_2-F-60μL. High-resolution narrow scan of the thrice-used SH-AuPd/SiO_2-F-60μL for **g**, Au(4d) and Pd(3d); **h**, S 2p, **i**, C(1s), the carbon numbering is shown in fig. **d**. The weak interaction between CO and the Au surface in **e** leads to only faint CO-Au vibrational features.[2]

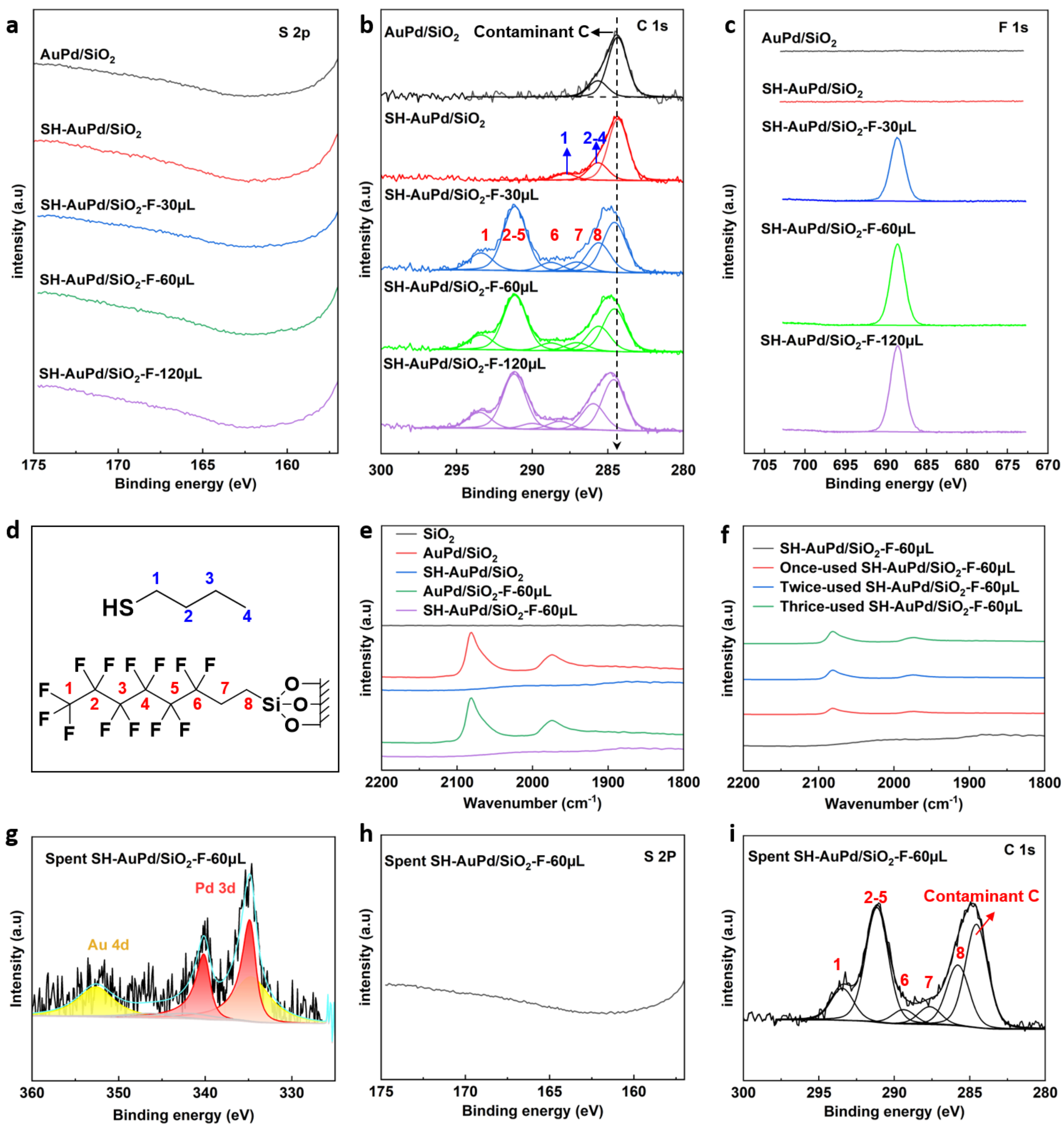

Supplementary Figure 4. Contact angle of water with pellets based on **a**, SH-AuPd/SiO_2; **b**, SH-AuPd/SiO_2-F-15 µL; **c,** SH-AuPd/SiO_2-F-30 µL; **d**, SH-AuPd/SiO_2-F-45 µL; **e**, SH-AuPd/SiO_2-F-60 µL; **f**, SH-AuPd/SiO_2-F-120 µL; **g**, SH-AuPd/SiO_2-C-60 µL; and **h**, spent SH-AuPd/SiO_2-C-60 µL. Contact angle for **i**, pure water; **j**, 20% methanol in water; **k**, 50% methanol in water; and **l**, 100% methanol with SH-AuPd/SiO_2-F-60 µL pellet. Contact angle for **m**, pure water; **n**, 20% isopropanol in water; **o**, 50% isopropanol in water; and **p**, 100% isopropanol with SH-AuPd/SiO_2-F-60 µL pellet.

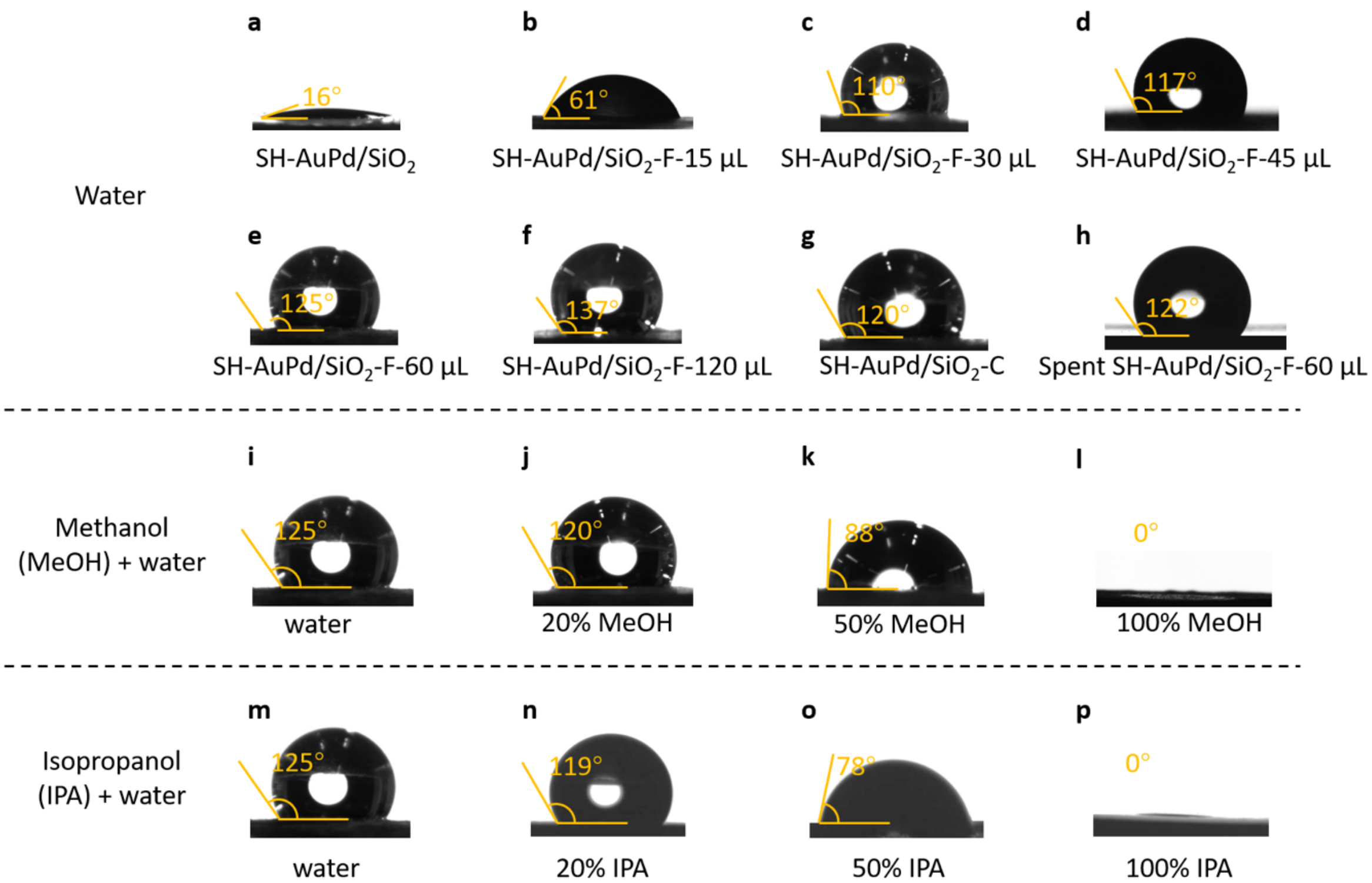

Supplementary Figure 5. Butanethiol adsorption on AuPd(111). Optimized geometry of butanethiol adsorbed on the AuPd(111) surface and calculated IR-spectra for butanethiol@AuPd (black) compared to gas-phase butanethiol (blue).

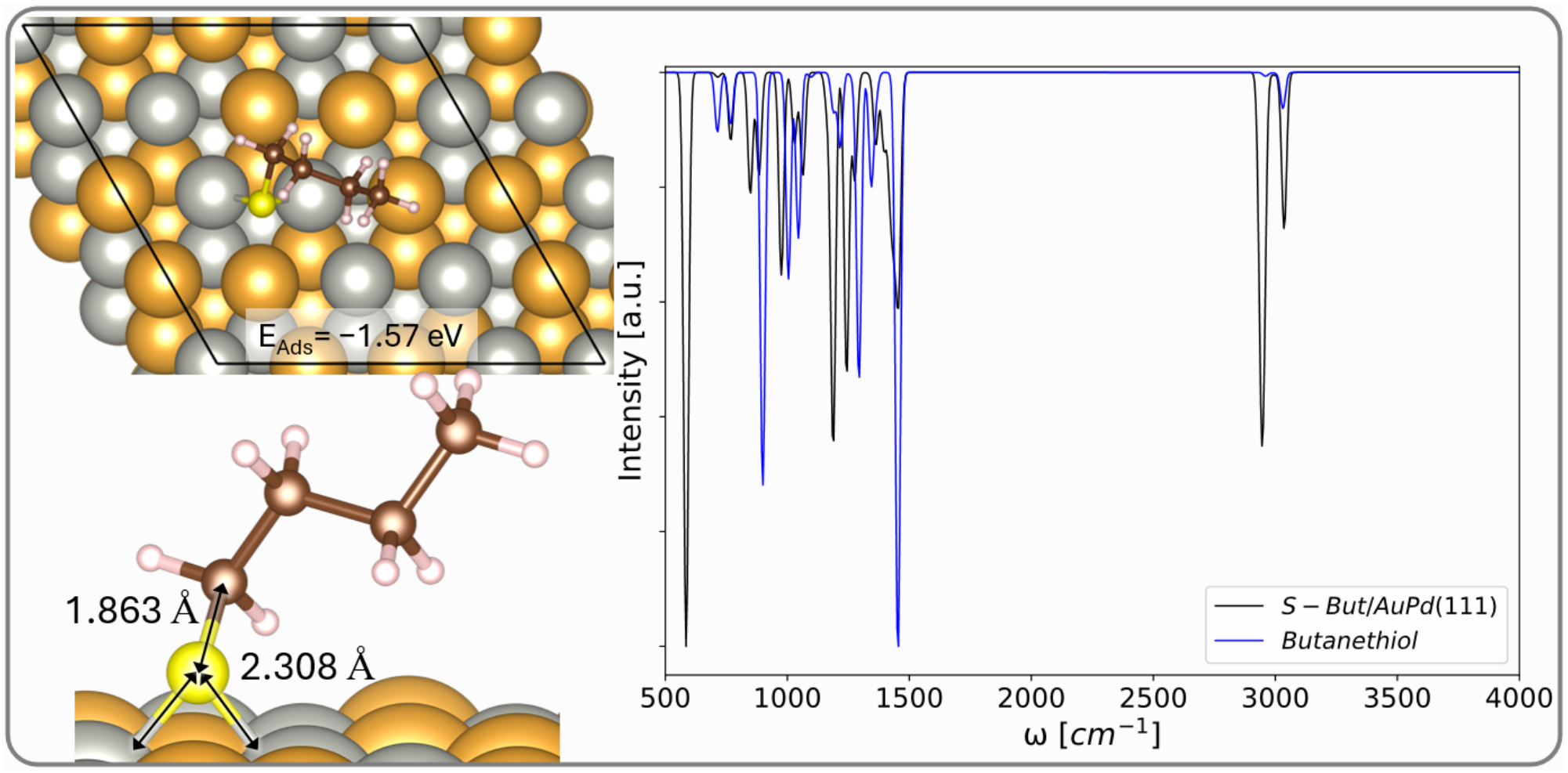

Supplementary Figure 6. H_2O_2 adsorption on AuPd(111). Optimized geometry of H_2O_2 adsorbed on the AuPd(111) surface and calculated IR-spectra for H_2O_2@AuPd(111) (black) compared to gas-phase H_2O_2 (blue).

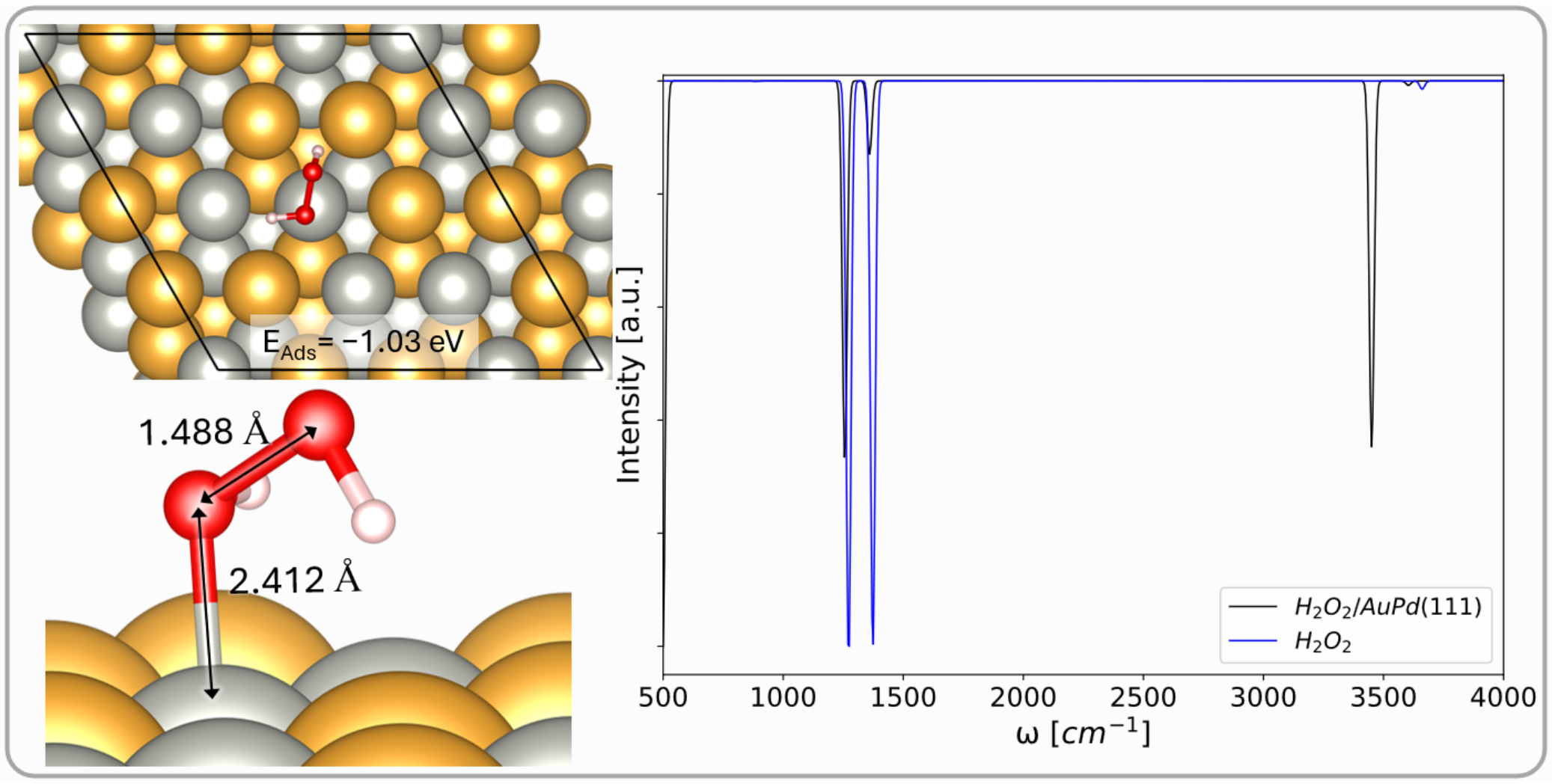

Supplementary Figure 7. H_2O_2/butanethiol co-adsorption on AuPd(111). Optimized geometry of co-adsorbed H_2O_2/butanethiol on the AuPd(111) surface and calculated IR-spectra for gas-phase H_2O_2 (blue) and adsorbed butanethiol@AuPd(111) (red).

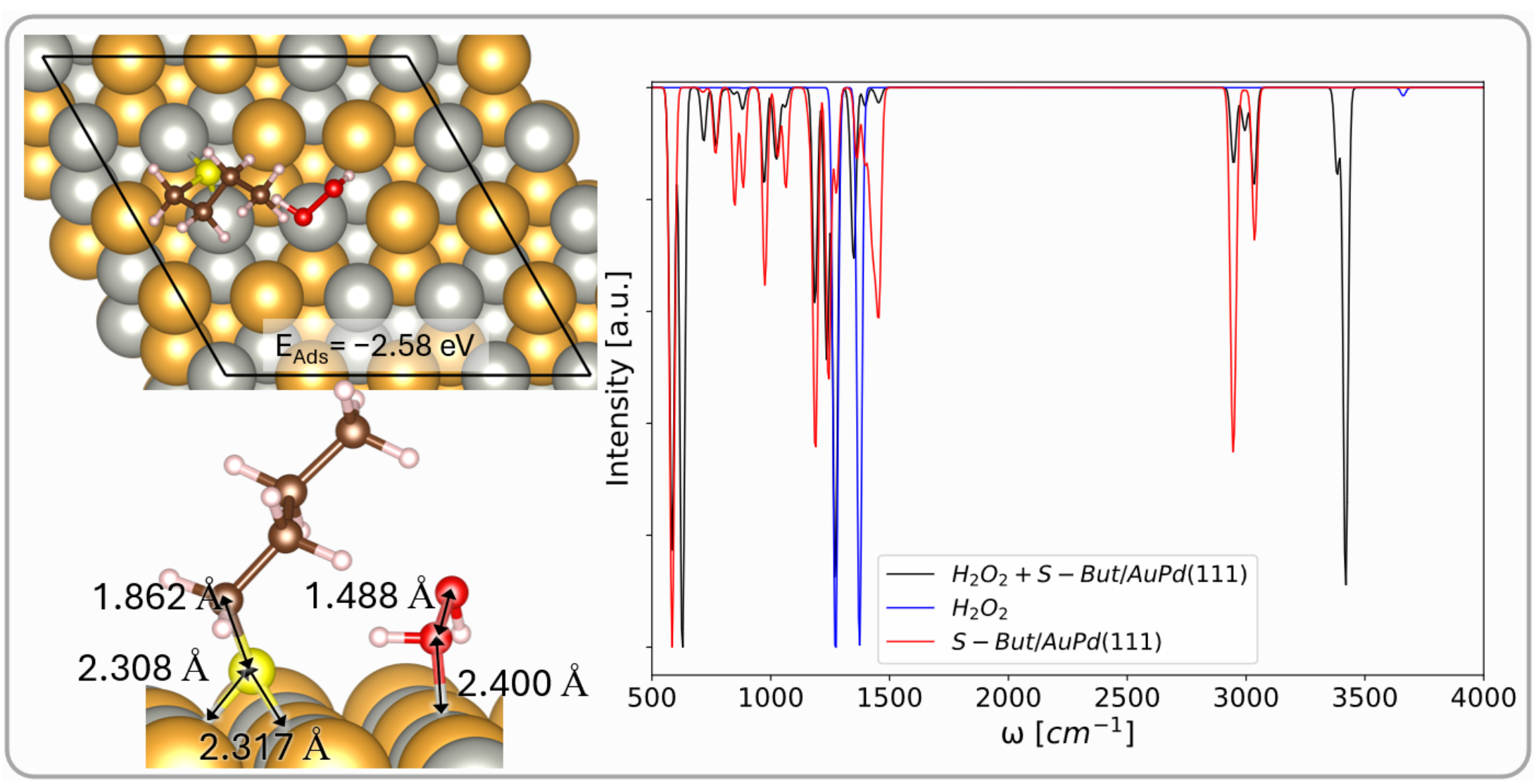

Supplementary Figure 8. O_2/butanethiol and H_2O co-adsorption on AuPd(111). Optimized geometry of (a) O_2 and (b) H_2O co-adsorbed with butanethiol on AuPd(111), together with calculated IR-spectra of the co-adsorbed systems, isolated gas-phase molecules, and adsorbed butanethiol@AuPd(111).

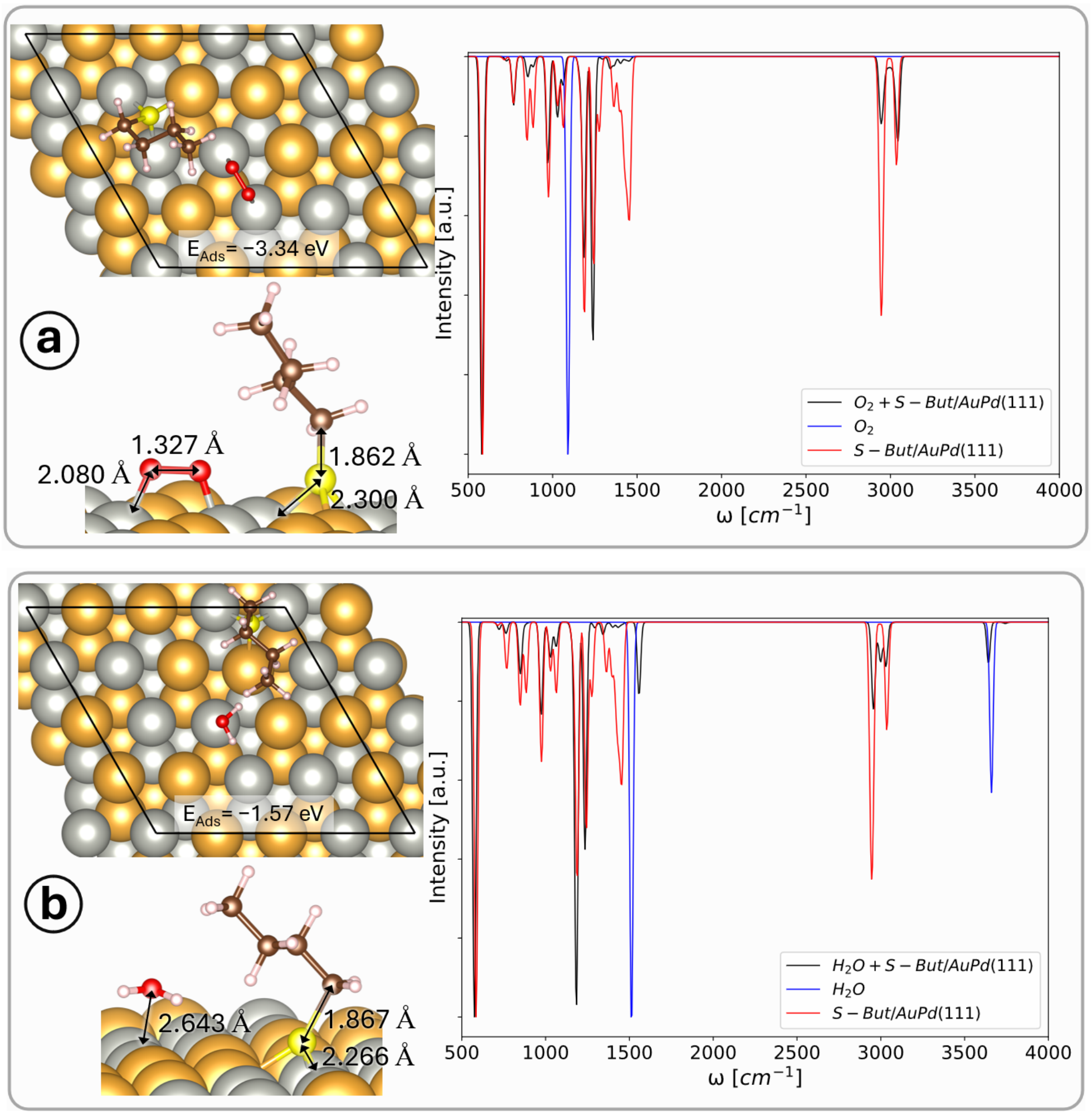

Supplementary Figure 9. Turbidity and zeta potential data. Optical images showing gas release from **a**, SH-AuPd/SiO$_2$-F-30μL; and **b**, SH-AuPd/SiO$_2$-F-120μL particles dispersed in water. Experimental conditions: 4% H_2/air, 3 bar, saturated for 20 min at 22 °C. The pressure was released at 0 s. **c**, c1,SH-AuPd/SiO$_2$-F-60μL particles aqueous dispersion. c2, Dropping methanol into SH-AuPd/SiO$_2$-F-60μL particles dispersion. c3, SH-AuPd/SiO$_2$ particles aqueous dispersion. c4, Dropping methanol into SH-AuPd/SiO$_2$ particles dispersion. Experimental conditions: ambient pressure, 22 °C, 100 μL methanol. **d**, Optical image of pure water in a cuvette, and SH-AuPd/SiO$_2$, SH-AuPd/SiO$_2$-F-60μL, and SH-AuPd/SiO$_2$-F-120μL particles dispersed in water in a cuvette. Conditions: 2.5 mg particles dispersed in 5 mL of water. **e**, Zeta potential of SH-AuPd/SiO$_2$, SH-AuPd/SiO$_2$-F-60μL, and Spent SH-AuPd/SiO$_2$-F-60μL dispersions in water. **f**, Correlation between the catalytic activity, plastron stability, and hydrophobicity of different particles . Particles: 16°: SH-AuPd/SiO$_2$, 61°: SH-AuPd/SiO$_2$-F-15μL, 110°: SH-AuPd/SiO$_2$-F-30μL, 117°: SH-AuPd/SiO$_2$-F-45μL, 125°: SH-AuPd/SiO$_2$-F-60μL, 137°: SH-AuPd/SiO$_2$-F-120μL. **g**, Time-evolution of absorbance of different hydrophobicity particle dispersions. Particles are SH-AuPd/SiO$_2$, SH-AuPd/SiO$_2$-F-15μL, SH-AuPd/SiO$_2$-F-30μL, SH-AuPd/SiO$_2$-F-45μL, SH-AuPd/SiO$_2$-F-60μL, and SH-AuPd/SiO$_2$-F-120μL **h**, Plastron stability of the SH-AuPd/SiO$_2$-F-60μL particle. **i**, Particle size distribution of SH-AuPd/SiO$_2$-F-60μL and Spent SH-AuPd/SiO$_2$-F-60μL. **j**, ^{1}H NMR spectrum of the filtrate after reaction. Experimental conditions: 10 ppm CH_3CN was used as the internal standard.

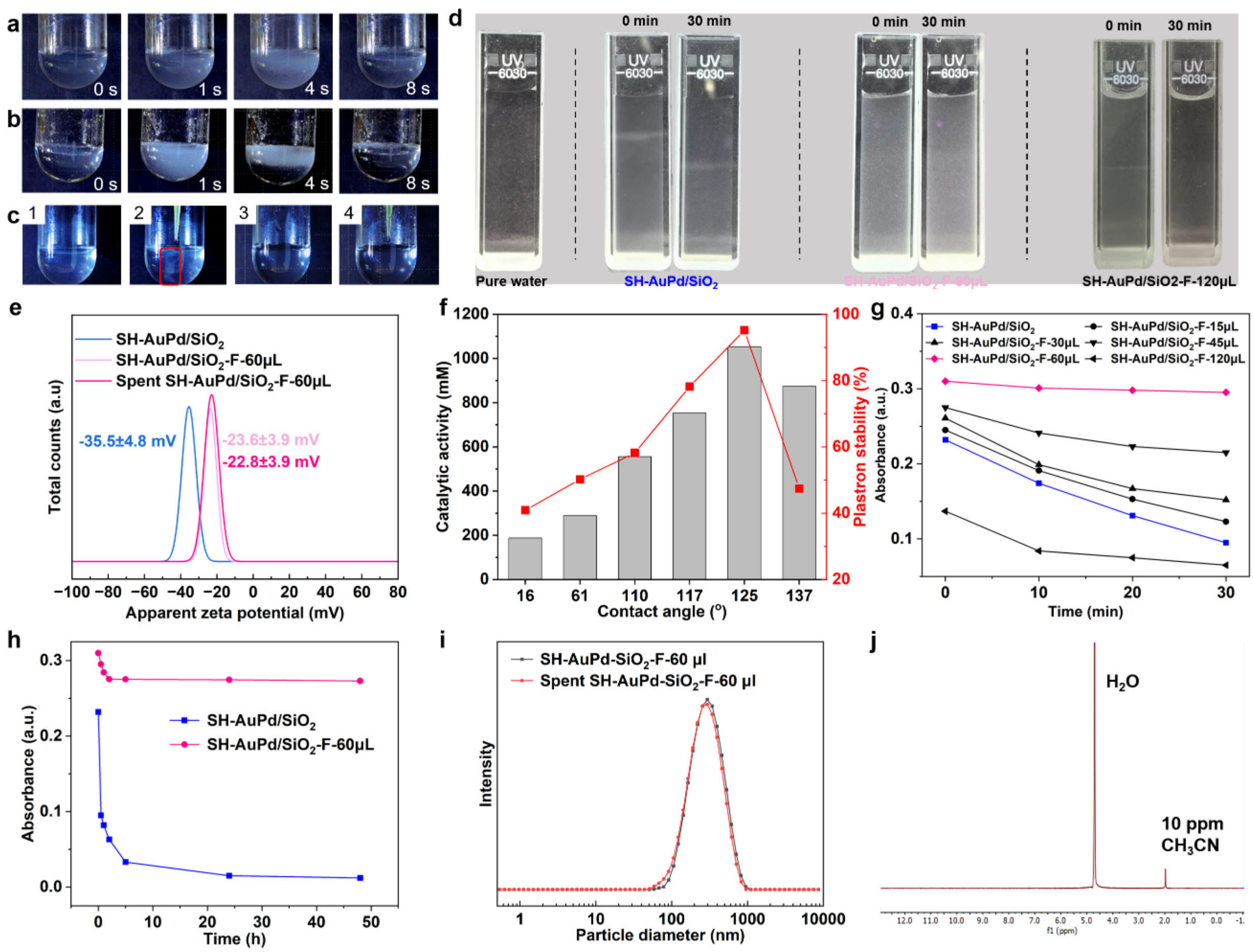

Supplementary Figure 10. Volume of Fluid simulation results. a, Computational domain with boundaries and **b**, hexahedral grid elements for calculations with the VoF method. **c**, Plastron stability for a hydrophobic particle at 0.015 s, 0.02 s, 0.025 s, 0.035 s. **d**, Streamline analysis after bubble attachment on a hydrophobic particle at 0.035 s.

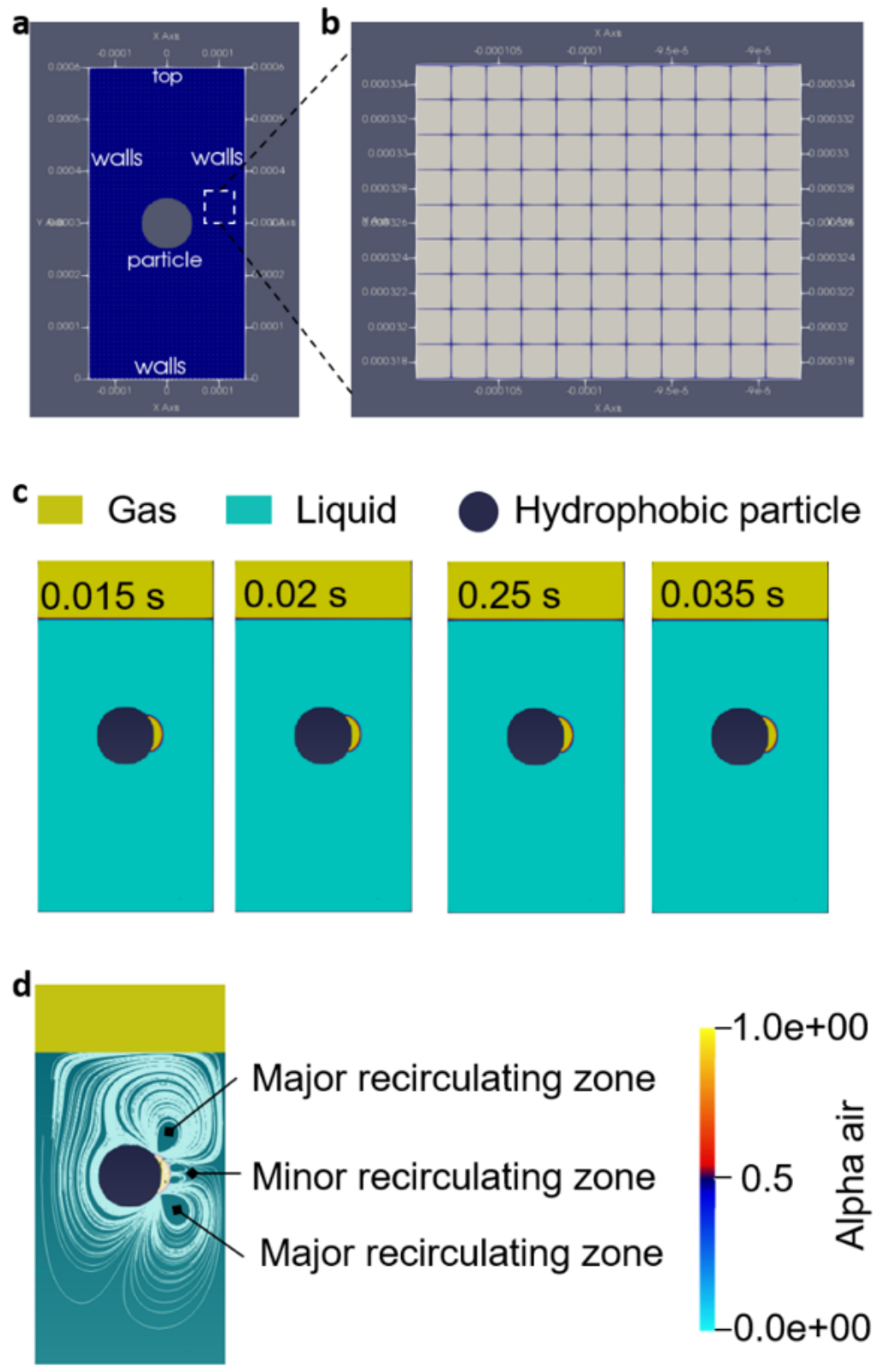

Supplementary Figure 11. Control experiments and catalyst reusability. a, Evolution of the H_2O_2 concentration for SH-AuPd/SiO_2-F-60μL particles at variable loading. Reaction conditions for H_2O_2 production: 5 mL of deionized water, H_2:Air=4%, 3 bar, 22 °C, 1500 rpm, 30 min. Reaction conditions for H_2O_2 degradation: 5 mL 2500 μM H_2O_2 solution, H_2:N_2=4%, 3 bar, 22 °C, 1500 rpm, 30 min, **b**, H_2O_2 synthesis and degradation activity of SiO_2-F and SiO_2 particles (circles, synthesis; squares, degradation). Reaction conditions for H_2O_2 synthesis: 2.5 mg particles, 5 mL H_2O, 3 bar H_2:Air = 4%, 22 °C, 1500 rpm, 30 min. Reaction conditions for H_2O_2 degradation: 2.5 mg particles, 5 mL of H_2O_2 (2500 μM), 3 bar H_2:N_2 = 4%, 22 °C, 1500 rpm, 30 min. **c**, H_2 conversion and H_2O_2 selectivity for SH-AuPd/SiO_2-F-60μL particles combined with noncatalytic particles. Reaction conditions: 2.5 mg of SH-AuPd/SiO_2-F-60μL combined with various amounts (2.5, 5, 10,100 mg) of noncatalytic SiO_2-F or SiO_2 particles, 5 mL H_2O, 3 bar H_2:Air = 4%, 22 °C, 1500 rpm, 30 min. **d**, H_2O_2 concentration in isopropanol/water mixtures at variable particle wettability. Reaction conditions: 5 mL of isopropanol/water mixture, H_2:Air=4%, 3 bar, 22 °C, 1500 rpm, 30 min. **e**, Kinetic plots of $-\text{Ln}(C_{H2O2})$ against time for the H_2O_2 direct synthesis catalyzed by SH-AuPd/SiO_2-F-60μL with plastron. **f**, Kinetic plots of $-\text{Ln}(C_{H2O2})$ against time for the H_2O_2 direct synthesis catalyzed by SH-AuPd/SiO_2 without plastron. **g**, Variation of TOF at time t=0 with reciprocal temperature for SH-AuPd/SiO_2-F-60μL and SH-AuPd/SiO_2. **h**, Conversion of H_2 and Selectivity of H_2O_2 for SH-AuPd/SiO_2, SH-AuPd/SiO_2-F-60μL, and thrice-used SH-AuPd/SiO_2-F-60μL particles. Reaction conditions: 2.5 mg particles, 5 mL of deionized water, H_2:Air=4%, 3 bar, 22 °C, 1500 rpm, 30 min. **i**, Evolution of the H_2O_2 concentration for AuPd/TiO_2 and SH-AuPd/SiO_2-F-60μL particles. Reaction conditions: 2.5 mg of catalyst, 5 mL of deionized water, 3 bar H_2:Air = 4%, 22 °C, 1500 rpm, 30 min. Error bars represent standard deviation.

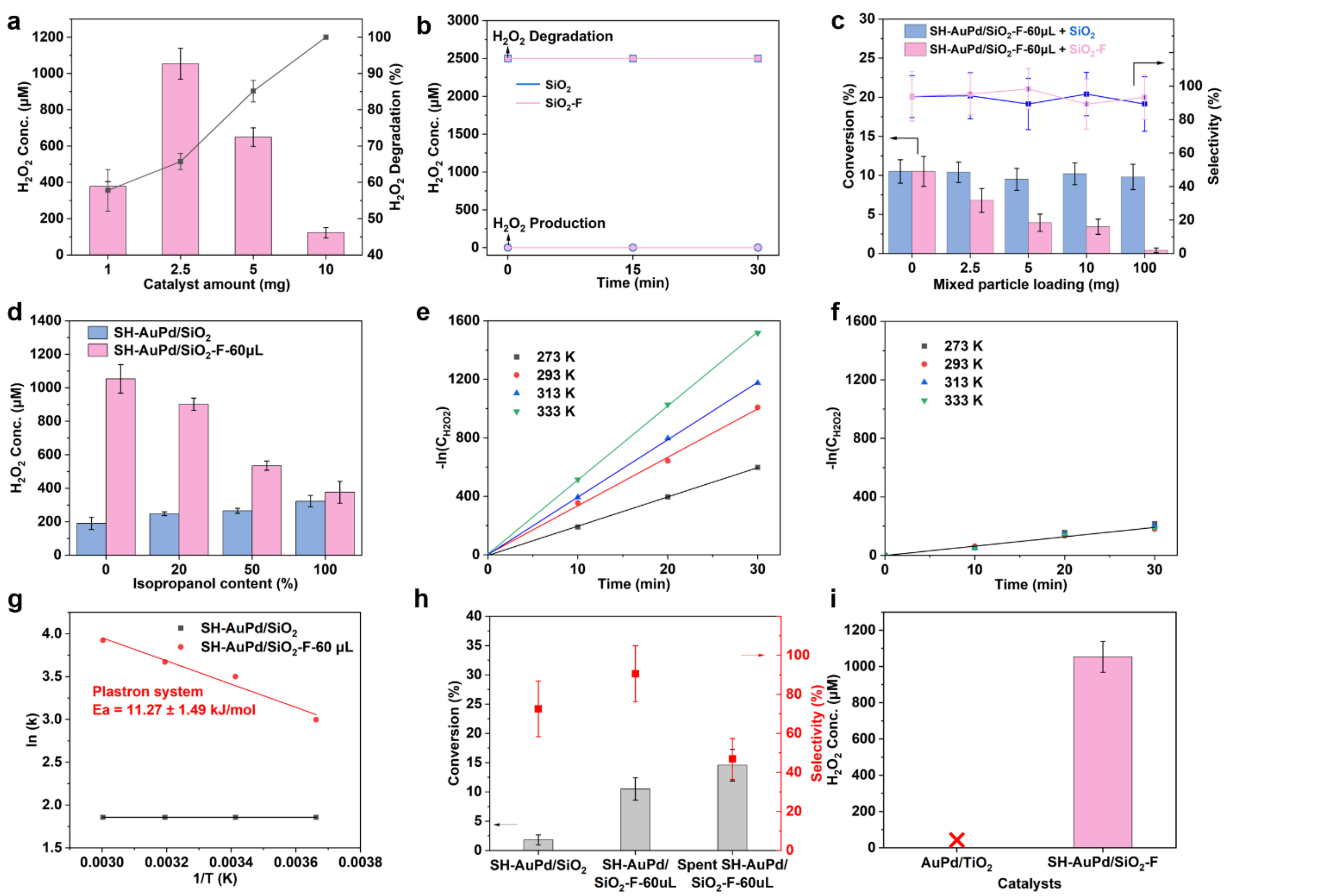

Supplementary Table 1. Main properties of catalytic particles.

Catalysts	Au (wt. %)[a]	Pd (wt. %)[a]	Grafting (wt. %)[b]	$\bar{D}_{alloy}$ (nm)[c]	Contact angle (°)[d]	Specific surface area (m^2/g)[e]
SiO_2	-	-	-	-	-	190
SH-AuPd/SiO_2	0.12	0.12	0	4.6 ± 0.86	16	210
SH-AuPd/SiO_2-F-30 μL	0.12	0.11	4.5	4.6 ± 0.90	110	-
SH-AuPd/SiO_2-F-60 μL	0.11	0.11	12.7	4.6 ± 0.95	125	196
SH-AuPd/SiO_2-F-120 μL	0.10	0.10	15.2	4.6 ± 0.88	137	-

[a] Measured by MP-AES; [b] The 1H,1H,2H,2H-perfluorooctyltriethoxysilane grafting amount is measured by TGA in the temperature range from 30 °C to 900 °C; [c] Measured by AC-STEM, [d] Measured by Dataphysics OCA 35 tensiometer, [e] Measured by N_2 adsorption/desorption at -196 °C (BET method).

Supplementary Table 2. Metal leaching tests.

Catalysts	Au (wt. %)[a]	Pd (wt. %)[a]	Au (μg/L)[a]	Pd (μg/L)[a]
SH-AuPd/SiO_2 (recycled)	0.12 ± 0.02	0.12 ± 0.02	-	-
SH-AuPd/SiO_2-F-60 μL (recycled)	0.11 ± 0.03	0.11 ± 0.02	-	-
SH-AuPd/SiO_2 (Filtrate after reaction)	-	-	4.50 ± 0.25	3.35 ± 0.12
SH-AuPd/SiO_2-F-60 μL (Filtrate after reaction)	-	-	0.35 ± 0.07	1.55 ± 0.05

[a] Measured by ICP-MS.

Supplementary Table 3. Catalytic activity comparisons against state-of-the-art catalysts for direct synthesis of H_2O_2.

Entry	Productivity /mol_{H2O2} kg_{metal}^{-1} h^{-1}	H_2O_2 conc. /ppm	Catalyst	Conditions (Temp., Pre., Solv.)	Gas composition	Ref.
1	1900	35	2.5 mg, 0.125%Au-0.125%Pd/SiO_2-F-60μL	25 °C, 3 bar, 5 mL H_2O	4% H_2/Air	This work
2	Max < 500	25.5	100mg, 1%Pd/resin	25 °C, 1 bar, 300 mL methanol /acetonitrile	H_2 flow 1 mL/min<br>O_2 flow 24 mL/min	Ref[3]
3	121	340	135 mg, 1.2%Pd-0.9%Au/sulphated ZrO_2	20 °C, 1 bar, 100 mL, 0.03 M H_2SO_4 methanolic solution	4% H_2/O_2 flow 50 ml/min	Ref[4]
4	66	150	135 mg, 2.5%PdO/F^- doped ZrO_2	20 °C, 1 bar, 100 mL, 0.03 M H_2SO_4 aqueous	4% H_2/O_2 flow 50 ml/min	Ref[5]
5	1300	200	10 mg, 0.5% Au-0.5% Pd/TiO_2	25 °C, 40 bar, 8.5 mL H_2O	420 psi 5% H_2/N_2<br>160 psi 25% O_2/N_2	Ref[6]
6	1000	480	10 mg, 2.5% Au-2.5% Pd/TiO_2	20 °C, 40 bar, 8.5 mL H_2O	420 psi H_2/CO_2<br>160 psi O_2/CO_2	Ref[7]
7	320	799	30 mg, 2.4%Au-2.3%Pd@HZSM-5	2 °C, 40 bar, 10 g of H_2O	10% O_2, 5% H_2, 45% Ar, 40% He	Ref[8]
8	1360	1100	10 mg, 3 wt % Pd-2 wt % Sn/TiO_2	2 °C, 40 bar, methanol (5.6 g), and water (2.9 g)	25% O_2/CO_2<br>5% H_2/CO_2	Ref[9]
9	3200	3200	10 mg, 2.5% Au-2.5% Pd/Acid-treated carbon	2 °C, 40 bar, methanol (5.6 g), and water (2.9 g)	25% O_2/CO_2<br>5% H_2/CO_2	Ref[10]
10	10559	NR	5 mg, 4.1%Pd_L/Pd-0.9%Sn-NW	0 °C, 40 bar, 5.54 g of MeOH, and 3.0 g of H_2O	10% O_2, 5% H_2, 85% Ar	Ref[11]

NR: not reported.

Supplementary References

1. Azcárate JC, Fonticelli MH, Zelaya E. Radiation damage mechanisms of monolayer-protected nanoparticles via TEM analysis. *The Journal of Physical Chemistry C* **121**, 26108-26116 (2017).
2. Carter JH*, et al.* Synergy and anti-synergy between palladium and gold in nanoparticles dispersed on a reducible support. *ACS catalysis* **6**, 6623-6633 (2016).
3. Sandri F*, et al.* The role of acetonitrile in the direct synthesis of hydrogen peroxide over palladium supported by ion-exchange resins. *Catalysis Communications* **174**, 106585 (2023).
4. Menegazzo F, Manzoli M, Signoretto M, Pinna F, Strukul G. H_2O_2 direct synthesis under mild conditions on Pd–Au samples: effect of the morphology and of the composition of the metallic phase. *Catalysis Today* **248**, 18-27 (2015).
5. Melada S, Rioda R, Menegazzo F, Pinna F, Strukul G. Direct synthesis of hydrogen peroxide on zirconia-supported catalysts under mild conditions. *Journal of Catalysis* **239**, 422-430 (2006).
6. Santos A*, et al.* Direct synthesis of hydrogen peroxide over Au–Pd supported nanoparticles under ambient conditions. *Industrial & Engineering Chemistry Research* **58**, 12623-12631 (2019).
7. Crole DA, Freakley SJ, Edwards JK, Hutchings GJ. Direct synthesis of hydrogen peroxide in water at ambient temperature. *Proceedings of the Royal Society A: Mathematical, Physical and Engineering Sciences* **472**, 20160156 (2016).
8. Jin Z*, et al.* Direct synthesis of pure aqueous H_2O_2 solution within aluminosilicate zeolite crystals. *ACS Catalysis* **11**, 1946-1951 (2021).
9. Freakley SJ*, et al.* Palladium-tin catalysts for the direct synthesis of H_2O_2 with high selectivity. *Science* **351**, 965-968 (2016).
10. Edwards JK*, et al.* Switching off hydrogen peroxide hydrogenation in the direct synthesis process. *Science* **323**, 1037-1041 (2009).
11. Li H-c*, et al.* Layered Pd oxide on PdSn nanowires for boosting direct H_2O_2 synthesis. *Nature Communications* **13**, 6072 (2022).
