# Supplementary material for: Plastron-mediated direct H2O2 synthesis
Source: Nat Commun. 2026 Aug 4;17:9389. doi: 10.1038/s41467-026-76198-9 (PMC13534698; doi:10.1038/s41467-026-76198-9)
Supplement: Supplementary file 2 [file 41467_2026_76198_MOESM2_ESM.pdf]

Description of Additional Supplementary Files

File name: Supplementary Movie 1

Description: Plastron release from catalysts with different hydrophobicity by depressurization. a, pure water, **b**, SH-AuPd/SiO_2, **c**, SH-AuPd/SiO_2-F-30 μL, **d**, SH-AuPd/SiO_2-F-60 μL, **e**, SH-AuPd/SiO_2-F-120 μL particles dispersed in water. Experimental conditions: 2 mg catalysts dispersed in 2 mL pure water by ultrasonication, 4% H_2:air, 3 bar, gas saturation for 20 min at 22 °C followed by depressurization, the pressure is removed at 0 s.

File name: Supplementary Movie 2

Description: Plastron release from catalysts with different hydrophobicity by adding low surface tension methanol. a, SH-AuPd/SiO_2, **b**, SH-AuPd/SiO_2-F-60 μL. Experimental conditions: 2 mg catalysts dispersed in 2 mL pure water by ultrasonication. 100 μL of methanol is added to the dispersion.
