# Supplementary material for: Plastron-mediated direct H2O2 synthesis
Source: Nat Commun. 2026 Aug 4;17:9389. doi: 10.1038/s41467-026-76198-9 (PMC13534698; doi:10.1038/s41467-026-76198-9)
Supplement: Supplementary file 3 [file 41467_2026_76198_MOESM5_ESM.pdf]

nature portfolio

Peer Review File

Plastron-Mediated Direct H2O2 Synthesis

Corresponding Author: Professor Marc Pera-Titus

This file contains all reviewer reports in order by version, followed by all author rebuttals in order by version.

Version 0:

Reviewer comments:

Reviewer #1

(Remarks to the Author)
Title: Plastron-Mediated Direct H2O2 Synthesis
Recommendation: Major Revision
Comment: This work reports a catalytic strategy based on hydrophobic interfacial engineering, in which thiol ligands and fluorinated silane modifiers are introduced onto the surface of AuPd/SiO2 catalysts to construct a stable gas plastron, thereby regulating the gas-liquid-solid three-phase interfacial structure. The authors propose that the presence of the gas plastron significantly enhances the local concentrations of H2 and O2 in the vicinity of the catalytic active sites, while simultaneously limiting the direct contact between the generated H2O2 and the metal surface, thus enabling efficient and selective direct H2O2 synthesis under ambient temperature and pressure in aqueous media. This catalytic system exhibits a high H2O2 production rate and selectivity without the need for acidic or halide promoters, and to some extent suppresses the subsequent degradation of H2O2, demonstrating the potential advantages of interfacial structure regulation in gas-liquid reactions. Overall, this study provides a novel strategy for modulating reaction interfaces via gas plastrons to enhance gas-liquid-solid reaction performance, and further expands the scope of hydrophobic interfacial engineering in catalytic systems. However, after careful examination of the manuscript, several key scientific questions and mechanistic interpretations remain to be further clarified, as detailed below.
1.In the characterization of AuPd/SiO2 modified withPFOTES, OTES, and SAMs, the authors primarily rely on FT-IR spectra and XPS to confirm the presence of PFOTES and SAMs. However, the specific binding sites of these functional groups are inferred only indirectly, mainly based on the disappearance of CO adsorption signals in CO-DRIFTS, which is interpreted as site blocking and thus coordination between thiols and metal sites (particularly Pd). Such evidence remains circumstantial and lacks direct structural characterization of the functional group binding locations. It is therefore recommended that more direct coordination evidence be provided to robustly support the proposed core mechanistic interpretation.

2.The manuscript does not provide a clear and experimentally verifiable definition of the true active sites responsible for the direct H2O2 synthesis. The CO-DRIFTS results only demonstrate that thiols block the CO adsorption sites on Pd, which is insufficient to identify whether H2O2 formation occurs on Au sites, Pd sites, or at the Au–Pd interfacial regions. It is therefore recommended that additional experimental evidence and/or theoretical validation be provided to explicitly clarify the nature and location of the active sites involved in H2O2 production.

3.The authors provide indirect evidence for the existence of the gas plastron based on the amount of bubble generation and confocal microscopy observations. However, key metrics such as the plastron thickness, stability, and its temporal evolution have not been quantitatively established. It would be highly beneficial to further characterize the dynamic formation process and long-term stability of the plastron, e.g., via in situ AFM or other suitable operando/interfacial probing techniques, to strengthen the central arguments of the manuscript.

4.In the manuscript, the authors tune the hydrophobicity of the catalysts by varying the PFOTES loading and attempt to correlate the hydrophobic nature of the catalyst with the ease of plastron formation and the resulting catalytic performance. However, excessively hydrophobic catalysts tend to float in the electrolyte, which suppresses plastron formation and leads to performance deactivation, thereby complicating the proposed correlation. It would therefore be valuable to design an expanded set of catalysts with graded hydrophobicity to systematically establish a correlation model linking contact angle, plastron stability, and catalytic performance.

5. The statement in the manuscript: “The H2O2 concentration increased …… to 560 μM and 1050 μM for SH-AuPd/SiO2-F-60 μL and SH-AuPd/SiO2-F-30 μL, respectively.” appears to be inconsistent with the labeling/order shown in Figure 3a1-a3.

Please verify and correct the correspondence between the sample labels and the reported H_2O_2 concentrations to avoid confusion.

6.The manuscript reports a pronounced bell-shaped dependence of the H_2O_2 concentration on catalyst loading for the SH-AuPd/SiO_2-F-60 μL catalyst, which is attributed to "competitive H_2O_2 decomposition activity." However, this explanation is currently based on qualitative reasoning and lacks direct experimental support. It is recommended that the authors provide additional evidence, such as H_2O_2 decomposition kinetics measurements or other relevant validation experiments, and discuss alternative physical factors that may contribute to the observed bell-shaped trend, in order to strengthen the robustness of the proposed mechanistic interpretation.

7.The enhanced selectivity is attributed to the "exclusion of H_2O_2 by the gas plastron, which reduces its contact with the catalyst surface." However, no direct evidence is provided to demonstrate the interfacial exclusion of H_2O_2 (e.g., interfacial concentration measurements, adsorption energetics, or in situ/operando characterization). Moreover, thiol modification itself can block Pd sites and suppress H_2O_2 decomposition, which is intertwined with the proposed plastron effect, making it difficult to disentangle the respective contributions of surface site blocking and gas-plastron-induced interfacial regulation. Clarification through targeted control experiments or additional mechanistic evidence would be necessary to deconvolute these effects.

8.The manuscript reports only two cycling runs, and a noticeable activity decay is observed. This raise concerns regarding whether the gas plastron can be sustainably maintained during long-term operation and whether the hydrophobic layer may be degraded by reactants and/or products. It is recommended that the authors extend the reaction duration to several hours and monitor both the plastron (gas-shield) stability and the evolution of catalytic activity over time. In addition, surface analyses of the post-reaction catalysts (e.g., XPS and contact-angle measurements) are suggested to evaluate the integrity of the hydrophobic layer and possible surface energy changes after operation.

9.The identification of the active sites and their mechanistic roles remain unclear. The manuscript emphasizes the formation of self-assembled monolayers (SAMs) of butanethiol to modulate surface properties and reports that CO-DRIFTS shows no CO adsorption after thiol treatment, suggesting Pd sites are blocked. Yet, the reaction proceeds efficiently, raising critical questions: What are the true active sites? If Pd is fully covered by thiols, how is H_2 activation occurring? Could there be exposed Au-Pd interfacial sites or defect sites contributing to activity? Do the hydrophobic chains (PFOTES/OTES) exclusively modify the SiO_2 support, or do they partially cover the metal nanoparticles as well? It is recommended that additional experiments be conducted to probe the chemical state of the metals under reaction conditions, and/or that theoretical studies be provided to elucidate the mechanistic role of the metal–support interface during the reaction.

Reviewer #2

(Remarks to the Author)
This manuscript presents a well-designed study on the use of gas layer trapped on a hydrophobic catalyst (plastron) to enhance direct H_2O_2 synthesis under mild conditions. The work combining catalyst synthesis, advanced characterization, simulation, and catalytic testing is commendable, which offers significant theoretical supports toward the development of direct H_2O_2 synthesis. However, some issues still need to be addressed before the further consideration of this manuscript.

1. While confocal microscopy and 2D numerical simulations suggest visualize plastron formation on catalyst surface, in-situ Raman/IR spectroscopy techniques are suggested to be further employed to directly analyze the reaction sites and the processes of direct H_2O_2 synthesis.
2. Please perform a more detailed kinetic study at different temperatures to determine the apparent activation energy (Ea) for H_2O_2 formation with and without the plastron. A significant difference in Ea would provide strong evidence for a change in the rate-limiting step or mechanism.
3. The production rate of H_2O_2 in the recycling experiment significantly decreased. The authors discussed that the observed increase in H_2 conversion for the spent catalyst could be attributed to enhanced gas-metal interactions due to the reduced coverage of thiol groups. Please provide XPS S 2p spectra of the fresh and spent catalysts to directly demonstrate the loss of S species. In addition, the Zeta potential, hydrophobicity, particle size distribution and other aspects of the catalyst before and after reaction should also be analyzed to comprehensively investigate the reasons for the decrease in activity.
4. Although SH-AuPd/SiO_2-F-60μL exhibits lower decomposition activity compared to the SH-AuPd/SiO_2, the H_2O_2 decomposition rate of SH-AuPd/SiO_2-F-60μL remains very high, which contradicts the fact that the hydrophobic modification significantly inhibits the decomposition process of H_2O_2. The stability of the catalyst may be an important factor influencing the decomposition of H_2O_2.
5. Figure 4a shows the Kinetic profiles for H_2O_2 synthesis over time, but H_2 conversion and H_2O_2 selectivity data of SH-AuPd/SiO_2 for the first ten minutes are missing.
6. Potential leaching of other elements (e.g., Si from support, F from PFOTES) and their impact on the activity/selectivity should be supplemented, such as the promoting effect of leaked halides.
7. The author needs to reorganize the layout, format and sequence of the images throughout the entire article. The current version of the readability of the images and writing require further refinement.
8. Minor. The reaction conditions of Supplementary Figure 7a were repeated.

Version 1:

Reviewer comments:

Reviewer #1

(Remarks to the Author)
The revised version is good enough and can be accepted in its current state.

Reviewer #2

(Remarks to the Author)
The authors have carefully addressed my comments. I suggest accept the revised manuscript for publication.

Open Access This Peer Review File is licensed under a Creative Commons Attribution 4.0 International License, which permits use, sharing, adaptation, distribution and reproduction in any medium or format, as long as you give appropriate credit to the original author(s) and the source, provide a link to the Creative Commons license, and indicate if changes were made.
In cases where reviewers are anonymous, credit should be given to 'Anonymous Referee' and the source.
The images or other third party material in this Peer Review File are included in the article's Creative Commons license, unless indicated otherwise in a credit line to the material. If material is not included in the article's Creative Commons license and your intended use is not permitted by statutory regulation or exceeds the permitted use, you will need to obtain permission directly from the copyright holder.
To view a copy of this license, visit https://creativecommons.org/licenses/by/4.0/

Answers to the comments of Reviewer 1

Comment: This work reports a catalytic strategy based on hydrophobic interfacial engineering, in which thiol ligands and fluorinated silane modifiers are introduced onto the surface of AuPd/SiO_2 catalysts to construct a stable gas plastron, thereby regulating the gas-liquid-solid three-phase interfacial structure. The authors propose that the presence of the gas plastron significantly enhances the local concentrations of H_2 and O_2 in the vicinity of the catalytic active sites, while simultaneously limiting the direct contact between the generated H_2O_2 and the metal surface, thus enabling efficient and selective direct H_2O_2 synthesis under ambient temperature and pressure in aqueous media. This catalytic system exhibits a high H_2O_2 production rate and selectivity without the need for acidic or halide promoters, and to some extent suppresses the subsequent degradation of H_2O_2, demonstrating the potential advantages of interfacial structure regulation in gas-liquid reactions. Overall, this study provides a novel strategy for modulating reaction interfaces via gas plastrons to enhance gas-liquid-solid reaction performance, and further expands the scope of hydrophobic interfacial engineering in catalytic systems. However, after careful examination of the manuscript, several key scientific questions and mechanistic interpretations remain to be further clarified, as detailed below.

We thank the reviewer for the warm consideration of our manuscript.

1.In the characterization of AuPd/SiO_2 modified with PFOTES, OTES, and SAMs, the authors primarily rely on FT-IR spectra and XPS to confirm the presence of PFOTES and SAMs. However, the specific binding sites of these functional groups are inferred only indirectly, mainly based on the disappearance of CO adsorption signals in CO-DRIFTS, which is interpreted as site blocking and thus coordination between thiols and metal sites (particularly Pd). Such evidence remains circumstantial and lacks direct structural characterization of the functional group binding locations. It is therefore recommended that more direct coordination evidence be provided to robustly support the proposed core mechanistic interpretation.

We thank the reviewer for this important remark. We agree that FT-IR, XPS, and CO-DRIFTS alone provide indirect evidence for the coordination mode of the thiol ligands. To more directly elucidate the binding configuration of the functional groups on the AuPd surface, we performed an extensive DFT investigation of sulfur adsorption on structurally optimized AuPd alloy surface.

The models used to construct the training set for AuPd-alloy global optimization were generated from a face-centered cubic (fcc) $Au_{32}Pd_{32}$ supercell using a Monte Carlo selection algorithm. A total of 14,400 structures were generated and subsequently relaxed by optimizing both atomic positions and cell parameters. The resulting structures were ranked by their relative energies using machine-learning force fields within the global optimization (MLFF-GO) workflow. The 12 most stable AuPd configurations identified were validated at an accurate DFT level (**Table 1**). Among them, the lowest-energy structure, $Au_{32}Pd_{32}$-01, was selected for detailed adsorption studies at the AuPd(111) surface.

Table 1. Twelve most stable AuPd alloy surfaces identified by MLFF-GO, with energies relative to the global minimum.

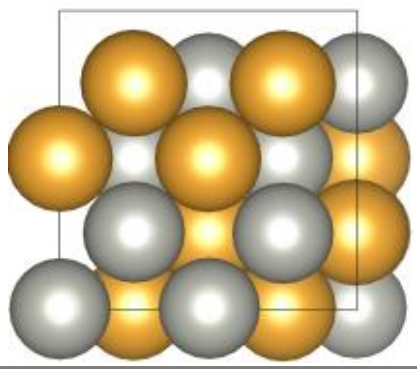	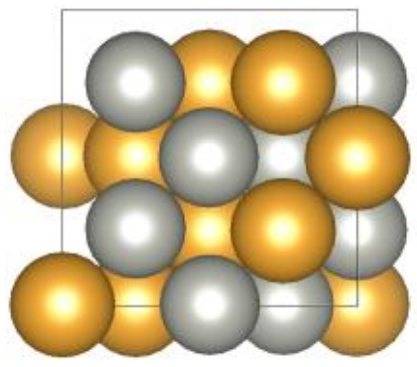	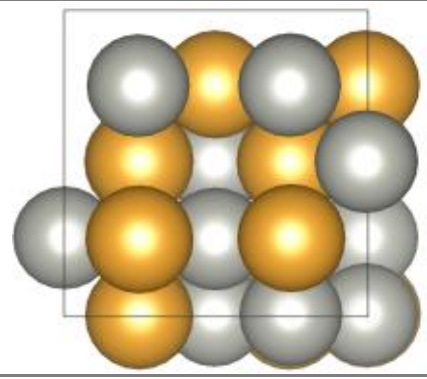
**$Au_{32}Pd_{32}$-01**<br>**ΔE = 0.000 eV**	$Au_{32}Pd_{32}$-02<br>ΔE = 0.018 eV	$Au_{32}Pd_{32}$-03<br>ΔE = 0.022 eV
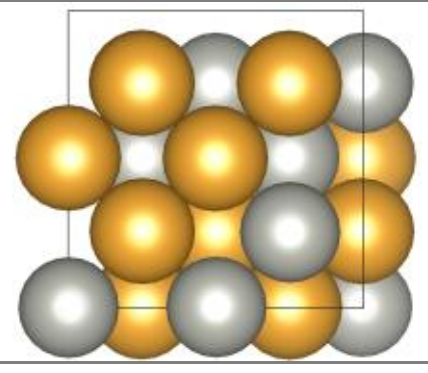	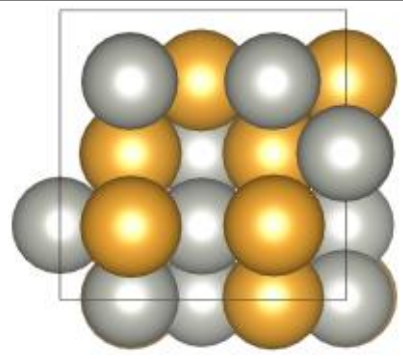	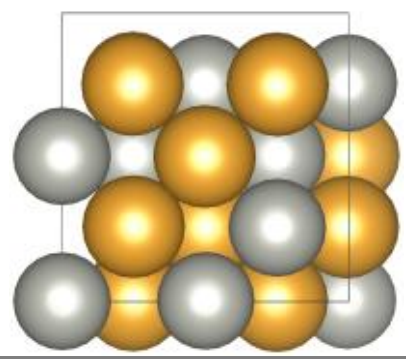
$Au_{32}Pd_{32}$-04<br>ΔE = 0.025 eV	$Au_{32}Pd_{32}$-05<br>ΔE = 0.028 eV	$Au_{32}Pd_{32}$-06<br>ΔE = 0.029 eV
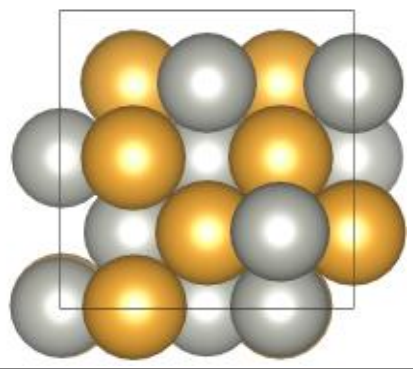	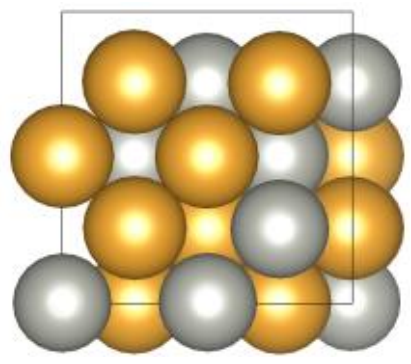	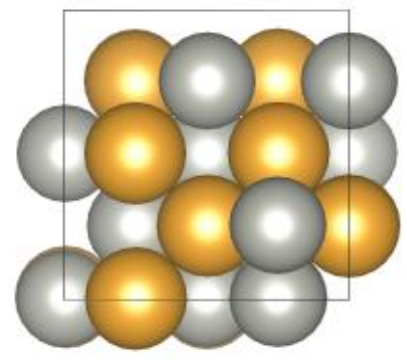
$Au_{32}Pd_{32}$-07<br>ΔE = 0.030 eV	$Au_{32}Pd_{32}$-08<br>ΔE = 0.032 eV	$Au_{32}Pd_{32}$-09<br>ΔE = 0.032 eV
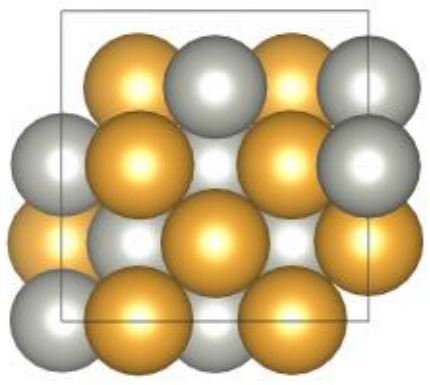	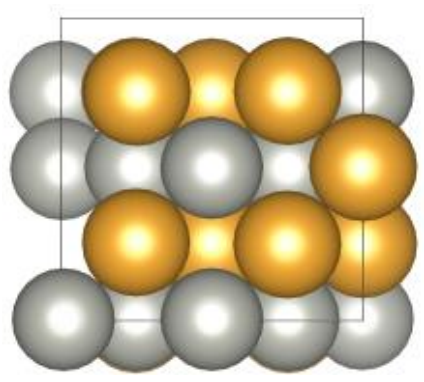	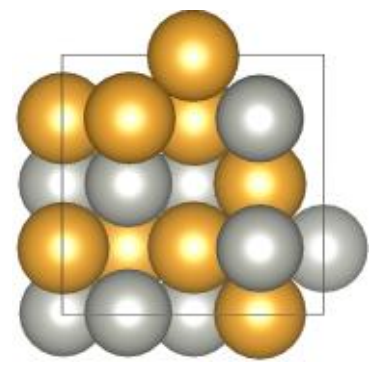
$Au_{32}Pd_{32}$-10<br>ΔE = 0.033 eV	$Au_{32}Pd_{32}$-11<br>ΔE = 0.034 eV	$Au_{32}Pd_{32}$-12<br>ΔE = 0.035 eV

We subsequently investigated the non-equivalent adsorption sites for butanethiol (S-but) on the $Au_{32}Pd_{32}$-01 surface (111) to identify the preferred coordination environment. The optimized geometry shows that the thiol preferentially adsorbs at a bridge site between two nearby surface Pd atoms, with Pd–S bond distances of 2.308 Å and with an adsorption energy of -1.57 eV (**Figure 1**).

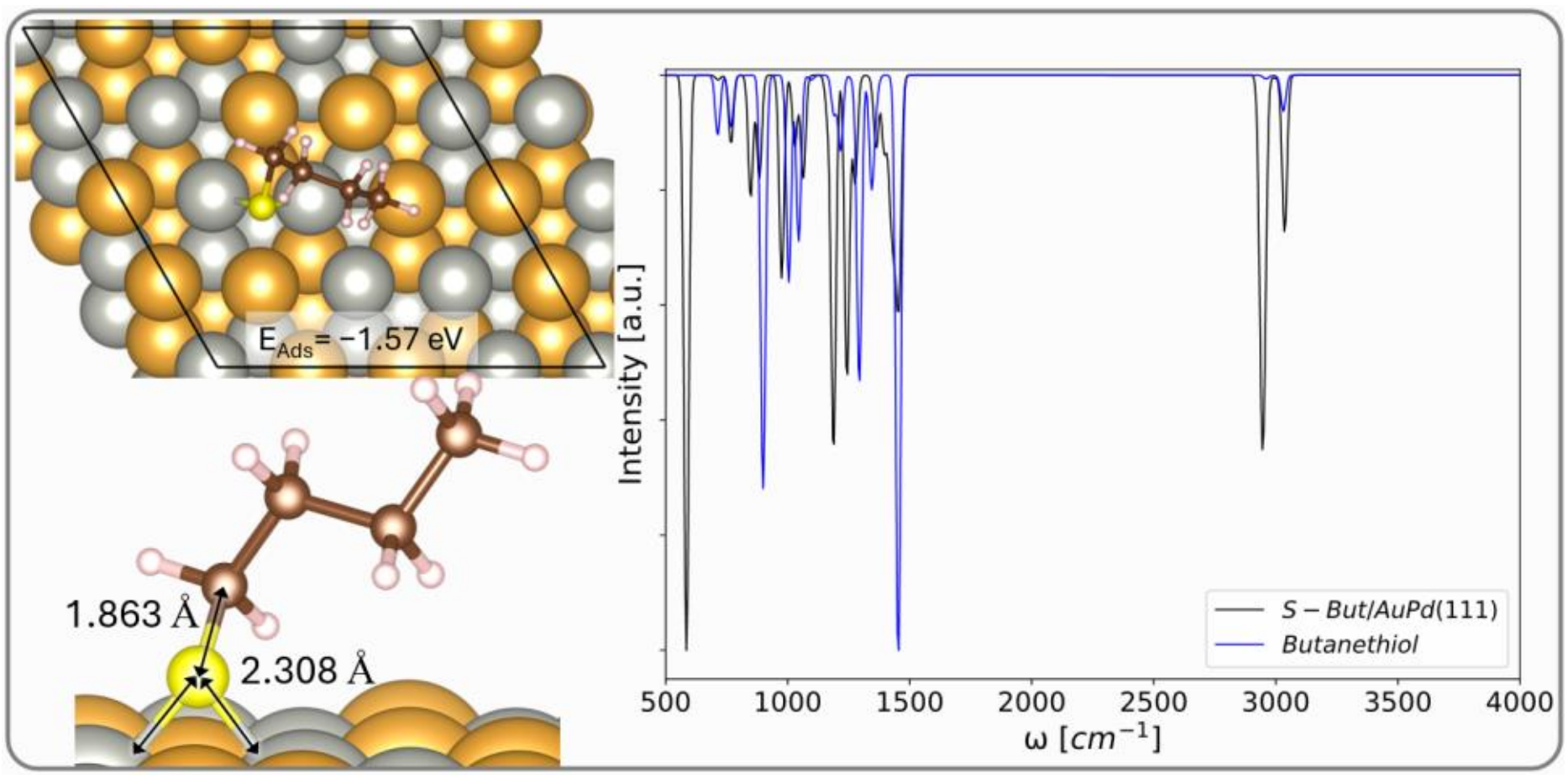

Figure 1. Optimized geometry of butanethiol adsorbed on the AuPd(111) surface and simulated IR-spectra for butanethiol@AuPd (black) compared to gas-phase butanethiol (blue).

To further support this assignment, vibrational spectra were calculated for both gas-phase butanethiol and adsorbed butanethiol@AuPd(111). Upon adsorption, the S–H stretching mode present in the gas phase disappears and is replaced by new vibrational features associated with Pd–S interactions, consistent with

dissociative chemisorption of the thiol on the metal surface. In addition, the S–C vibrational mode shifts from approximately 900 cm^{-1} in the gas phase to around 1200 cm^{-1} upon adsorption. This shift is accompanied by an elongation of the S–C bond from 1.845 Å to 1.863 Å, indicating modification of the sulfur electronic environment upon coordination to Pd.

These theoretical results strongly support the interpretation derived from the CO-DRIFTS experiments. Specifically, the preferential adsorption of sulfur on bridged Pd sites is consistent with the disappearance of bridged CO adsorption bands after thiol functionalization in SH-AuPd/SiO_2 and SH-AuPd/SiO_2-F-60 μL. Besides, the fact that linear CO adsorption bands vanish can be explained by the presence of electron-depleted Pd sites surrounded by Au and adsorbed butanethiol discouraging Pd $\rightarrow$ CO π-back donation. Taken together, the DFT adsorption geometries, adsorption energetics, and computed vibrational signatures provide direct molecular-level evidence of Pd–S coordination and substantiate the proposed mechanistic interpretation of thiol-induced surface modification.

For clarity, the above **Figure 1** has been added to the Supplementary Information (**Supplementary Figure 5**), and the section 'Characterization of hydrophobic catalysts' has been updated as follows:

'Analysis by diffuse reflectance infrared Fourier transform spectroscopy, using CO as a probe molecule~~.~~, was used to assess surface accessibility of metal sites. CO-DRIFTS analysis of AuPd/SiO_2 and AuPd/SiO_2-F-60 μL catalysts reveals typical CO adsorption bands on Pd at ~2050-2100 cm^{-1} (linear mode) and ~1800-2000 cm^{-1} (bridged mode), indicating the presence of accessible metallic sites for CO binding (**Supplementary Figure 3e**).[26] In contrast, the thiol-functionalized samples, SH-AuPd/SiO_2 and SH-AuPd/SiO_2-F-60 μL, show no detectable CO adsorption features, which is attributed to strong coordination between Pd(0) centers and thiolate ligands, ~~which~~ effectively blockings~~s~~ CO access to the active Pd sites.[27,28]

Density functional theory (DFT) calculations further substantiate this interpretation. The results show that butanethiol preferentially adsorbs on Pd sites of the AuPd surface, binding at bridge positions with Pd–S bond distances of ~2.31 Å and a strong adsorption energy of -1.57 eV (**Supplementary Figure 5**). This selective chemisorption leads to effective occupation of Pd surface ensembles. The calculated binding configuration therefore provides molecular-level evidence that thiol ligands directly coordinate to nearby Pd atoms, rationalizing the disappearance of bridged-type CO adsorption bands observed experimentally upon thiol functionalization. Besides, the fact that linear CO adsorption bands vanish can be explained by the presence of electron-depleted isolated Pd sites surrounded by Au and adsorbed butanethiol discouraging Pd $\rightarrow$ CO π-back donation.

To further support this assignment, vibrational spectra were calculated for both gas-phase butanethiol and adsorbed butanethiol@AuPd(111) (**Supplementary Figure 5**). Upon adsorption, the S–H stretching mode present in the gas phase disappears and is replaced by new vibrational features associated with Pd–S interactions, consistent with dissociative chemisorption of the thiol on the metal surface. In addition, the S–C vibrational mode shifts from approximately 900 cm^{-1} in the gas phase to around 1200 cm^{-1} after adsorption. This shift is accompanied by elongation of the S–C bond from 1.845 Å to 1.863 Å, indicating modification of the sulfur electronic environment upon coordination to Pd.'

Besides, a computational section has been added to the Methods section.

2.The manuscript does not provide a clear and experimentally verifiable definition of the true active sites responsible for the direct H_2O_2 synthesis. The CO-DRIFTS results only demonstrate that thiols block the CO adsorption sites on Pd, which is insufficient to identify whether H_2O_2 formation occurs on Au sites, Pd sites, or at the Au-Pd interfacial regions. It is therefore recommended that additional experimental evidence and/or theoretical validation be provided to explicitly clarify the nature and location of the active sites involved in H_2O_2 production.

We thank the reviewer for this important remark. We agree that the original CO-DRIFTS results alone cannot unambiguously identify the true active sites responsible for H_2O_2 formation. To clarify the nature of the catalytically active surface ensembles, we performed additional DFT calculations to investigate the adsorption and co-adsorption behavior of H_2O_2, O_2, H_2O, and butanethiol on the AuPd(111) alloy surface.

Using the computational methodology described above, we first investigated the preferred adsorption site for H_2O_2 on the AuPd surface. The calculations show that H_2O_2 adsorbs most favorably on top of Pd atoms, with an adsorption energy of -1.03 eV and a Pd–O bond distance of 2.412 Å (**Figure 2**). Upon adsorption, the O–O bond length increases slightly from 1.483 Å in the gas phase to 1.488 Å at the surface, indicating

moderate activation of the peroxide molecule. In addition, new vibrational modes appear near 500 and 3500 cm^{-1} in the calculated IR spectrum, confirming interaction between H_2O_2 and the metal surface.

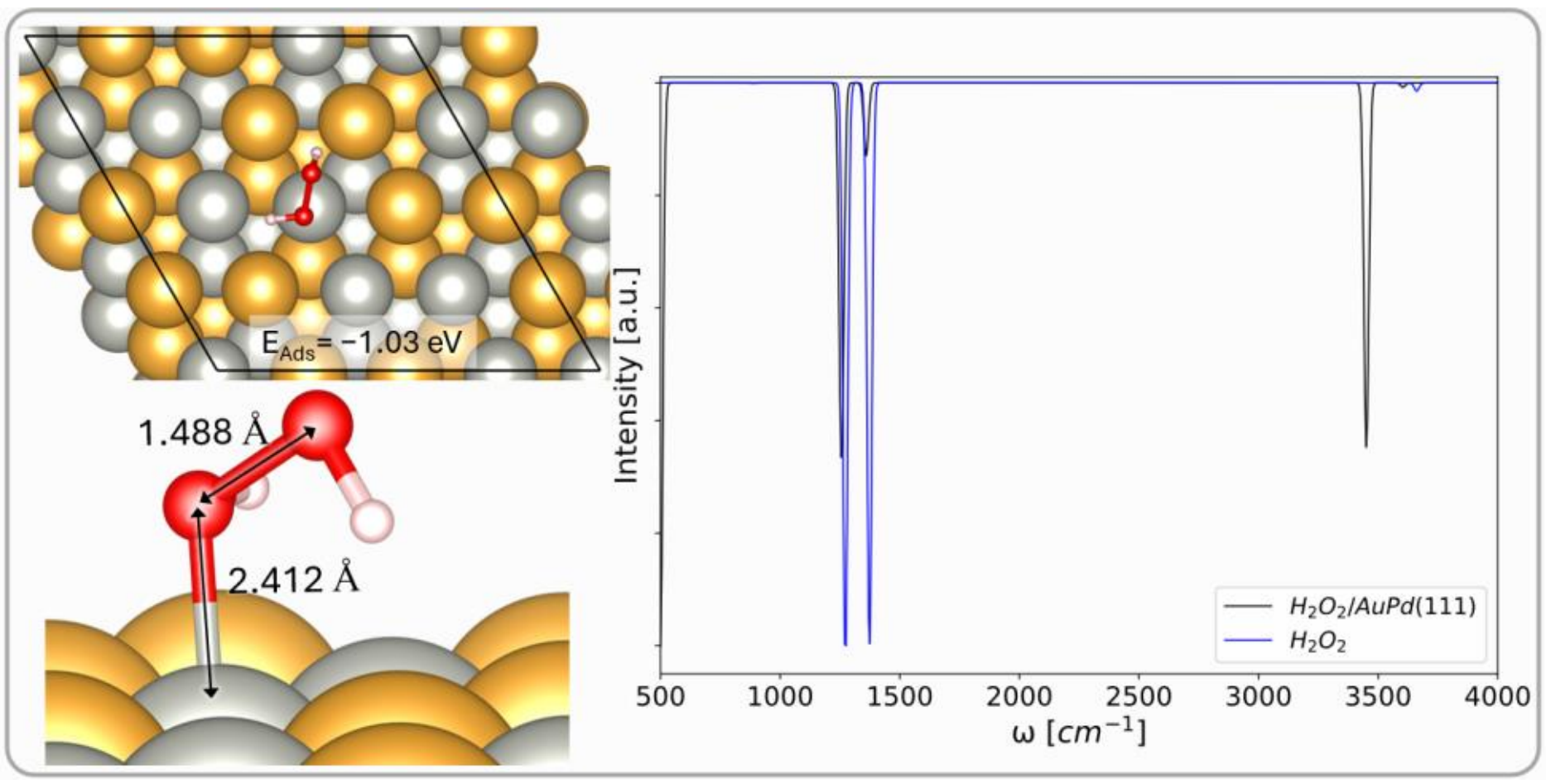

Figure 2. Optimized geometry of H_2O_2 adsorbed on the AuPd(111) surface and simulated IR-spectra for H_2O_2@AuPd(111) (black) compared to gas-phase butanethiol (blue).

To further understand the influence of thiol ligands on the catalytic interface, we subsequently investigated the co-adsorption of butanethiol (S-but) and H_2O_2 on the AuPd(111) surface. In the optimized structure, both molecules retain their preferred adsorption configurations: butanethiol adsorbs at a Pd–Pd bridge site, whereas H_2O_2 adsorbs on top of a neighboring Pd atom, yielding a total adsorption energy of -2.58 eV (**Figure 3**). The sulfur atom maintains two Pd–S interactions with bond distances of 2.308 and 2.317 Å, while the Pd–O distance for adsorbed H_2O_2 decreases slightly from 2.412 Å for isolated H_2O_2 adsorption to 2.400 Å in the co-adsorbed system. This result indicates that thiol adsorption does not inhibit H_2O_2 adsorption on adjacent Pd sites and may even strengthen the local metal–adsorbate interaction through cooperative electronic effects.

To probe the nature of the catalytically active sites during H_2O_2 synthesis, we further compared the co-adsorption behavior of O_2 and H_2O in the presence of butanethiol (**Figure 4a**). The calculations reveal that O_2 adsorbs preferentially on Pd-rich site ensembles with a strong total adsorption energy of -3.34 eV, i.e., in the co-adsorbed configuration with butanethiol. The O_2 molecule bridges neighboring Pd atoms with Pd–O distances of approximately 2.080 Å, while the O–O bond elongates substantially from 1.240 Å in the gas phase to 1.327 Å after adsorption. This pronounced bond activation demonstrates that Pd-containing sites remain highly active for O_2 activation even in the presence of coordinated thiol ligands.

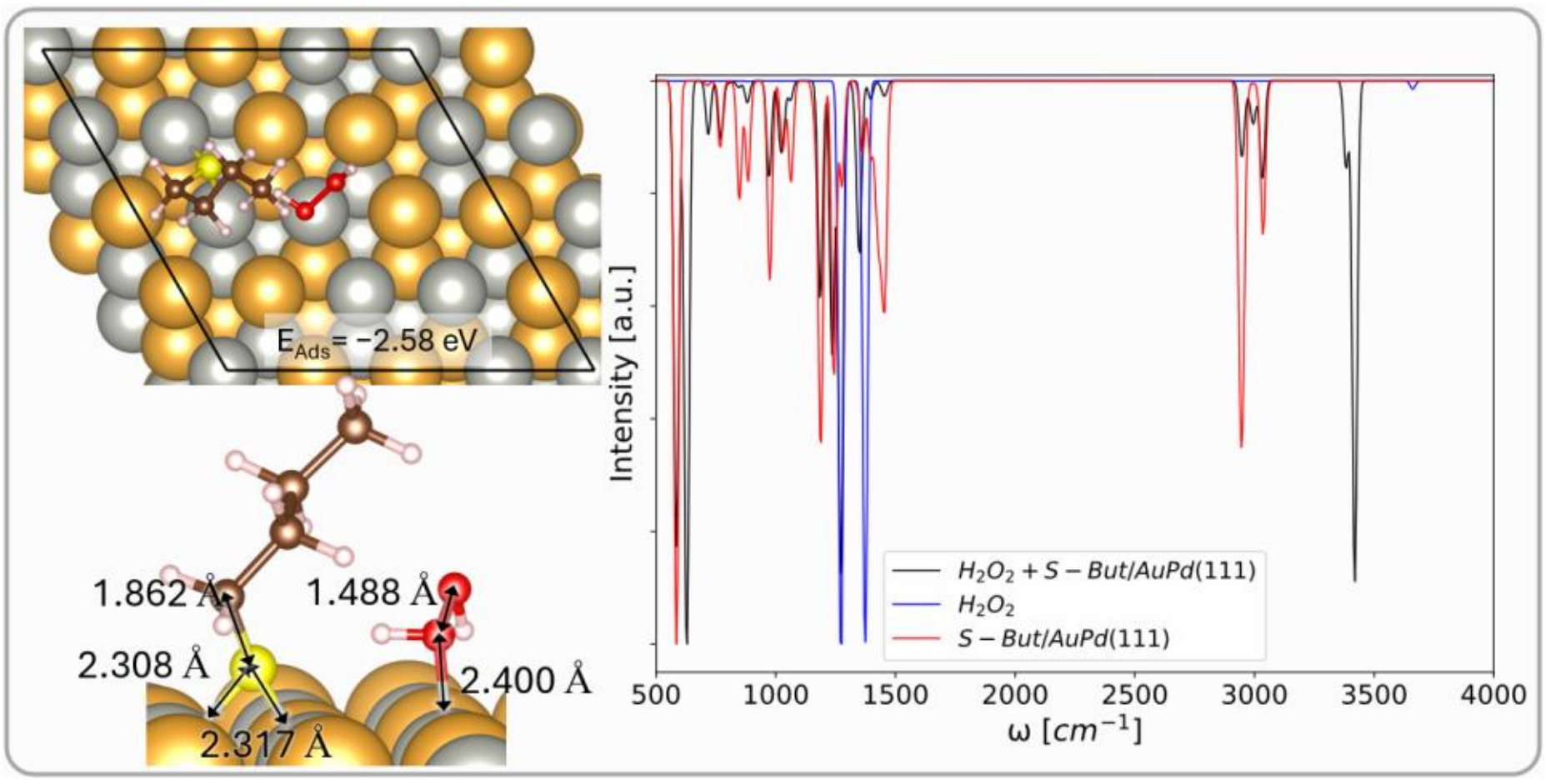

Figure 3. Optimized geometry of co-adsorbed H_2O_2/butanethiol on AuPd(111) and simulated IR-spectra for gas-phase H_2O_2 (blue) and adsorbed butanethiol@AuPd(111) (red).

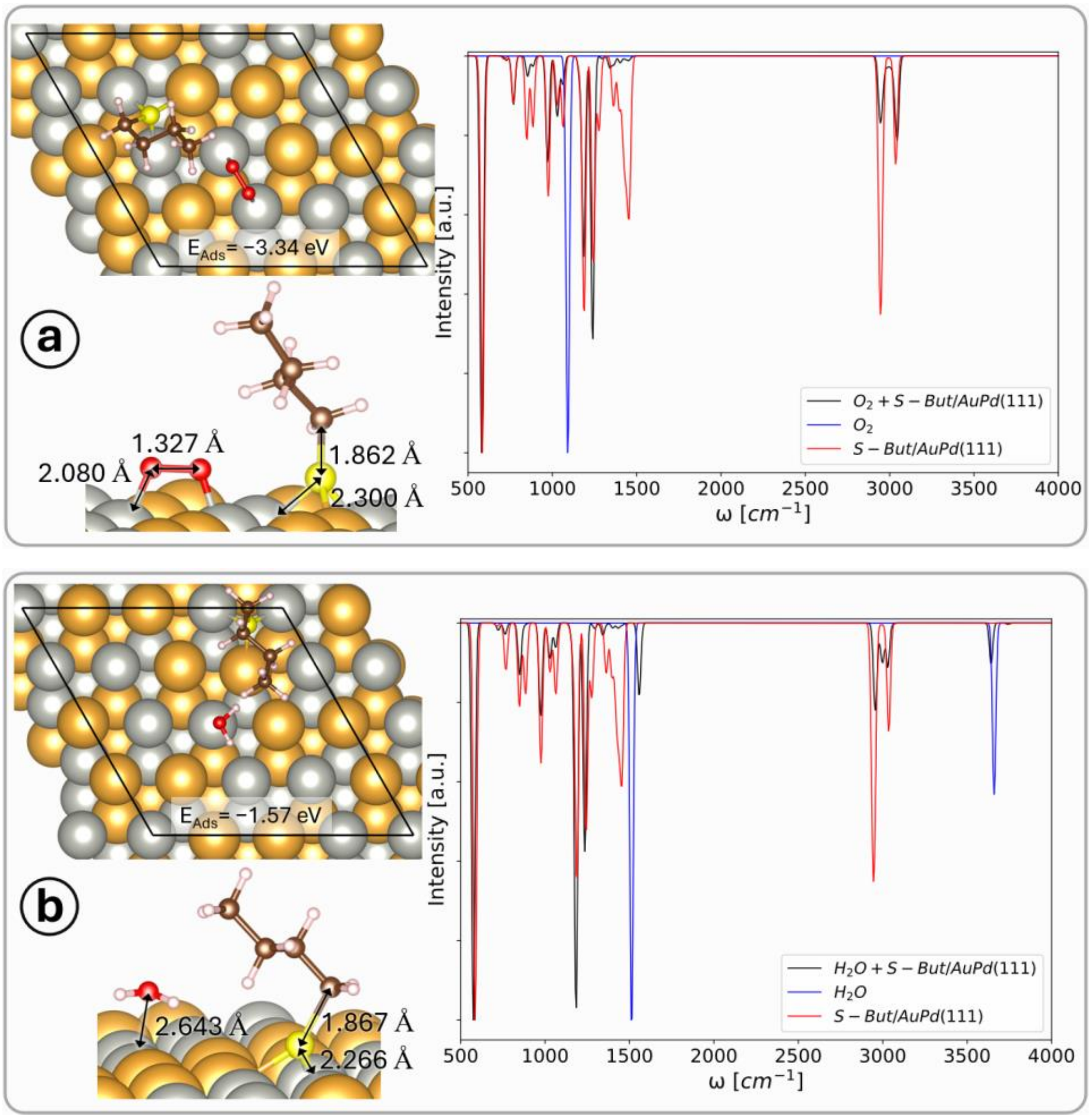

Figure 4. Optimized geometry of (a) O_2 and (b) H_2O co-adsorbed with butanethiol on AuPd(111), together with simulated IR-spectra of the co-adsorbed systems, isolated gas-phase molecules, and adsorbed butanethiol@AuPd(111).

Interestingly, the adsorption geometry of butanethiol changes under O_2 co-adsorption from a simple Pd–Pd bridge configuration to a threefold interaction involving two Pd atoms and one Au atom, with metal–S distances around 2.3 Å. This observation suggests that Au–Pd interfacial ensembles participate in stabilizing the thiol-modified catalytic surface. In contrast, H_2O adsorption near the thiol-functionalized surface is significantly weaker, with a co-adsorption energy of only -1.57 eV (**Figure 4b**). Water adsorbs weakly on top of Pd atoms at a Pd–O distance of 2.643 Å. At the same time, the butanethiol ligand maintains its threefold metal interaction geometry, yet closer to the Pd atoms.

The calculated adsorption energies are ranked in the following order:

$$E_{ads}(\text{S-but}, O_2) > E_{ads}(\text{S-but}, H_2O_2) > E_{ads}(\text{S-but}, H_2O).$$

These results provide several important mechanistic insights. First, the presence of butanethiol does not suppress the ability of Pd-containing surface ensembles to adsorb and activate O_2, indicating that partially thiol-decorated Pd sites remain catalytically accessible. Second, the stronger adsorption of O_2 and H_2O_2 relative to H_2O suggests that the thiol-modified hydrophobic interface preferentially stabilizes reactants and products involved in the direct H_2O_2 synthesis pathway while disfavoring water accumulation near the active sites. Since adsorbed water and surface hydroxyl species are known to facilitate H_2O_2 decomposition through proton-transfer and O–O bond cleavage pathways (Plauck *et al*, *Proc. Natl. Acad. Sci. USA*, **2016**, *113*, E1973-E1982), weaker water adsorption is expected to enhance H_2O_2 stability.

Taken together, these results support a mechanism in which partially thiol-decorated Pd sites and Au–Pd interfacial ensembles serve as the primary active sites for direct H_2O_2 synthesis. The thiol ligands do not fully passivate the metal surface; rather, they selectively modify the local adsorption environment, preserving O_2 activation capability while suppressing water-assisted H_2O_2 decomposition pathways.

The manuscript has been revised accordingly to clarify the nature and location of the catalytically active sites. For clarity, the above **Figures 2-4** have been added to the Supplementary Information (**Supplementary Figures 6-8**) and the remaining figures have been renumbered accordingly. Also, the following section has been added after 'Characterisation of hydrophobic catalysts':

'Computational investigation of the active sites for H_2O_2 synthesis. *To further clarify the nature of the active sites involved in direct H_2O_2 synthesis, density functional theory (DFT) calculations were performed on the AuPd(111) surface to investigate the adsorption behavior of H_2O_2, O_2, H_2O, and butanethiol (S-but). The calculations reveal that H_2O_2 adsorbs most favorably on top of Pd atoms with an adsorption energy of -1.03 eV and a Pd–O distance of 2.412 Å (**Supplementary Figure 6**). Upon adsorption, the O–O bond length increases slightly from 1.483 Å in the gas phase to 1.488 Å at the AuPd surface, accompanied by the appearance of new vibrational modes at 500 and 3500 cm^{-1}, indicating direct interaction between H_2O_2 and Pd surface atoms.*

*We next investigated the co-adsorption of butanethiol and H_2O_2 on AuPd(111). The calculations show that butanethiol preferentially adsorbs at a Pd–Pd bridge configuration, while H_2O_2 remains adsorbed on top of a Pd atom, resulting in a total adsorption energy of -2.58 eV (**Supplementary Figure 7**). The adsorbed thiolate forms two Pd–S interactions with bond distances of 2.308 and 2.317 Å. Interestingly, the Pd–O distance for H_2O_2 decreases from 2.412 Å in the isolated adsorption state to 2.400 Å in the co-adsorbed configuration, suggesting a strengthened metal–adsorbate interaction through synergistic effects between the two adsorbates.*

*To further probe the active sites involved in the reaction pathway, we compared the co-adsorption of butanethiol with O_2 and H_2O on AuPd(111). O_2 adsorbs preferentially on Pd-rich regions, forming a bridge configuration between neighboring Pd atoms with an adsorption energy of -3.34 eV (**Supplementary Figure 8**). The Pd–O bond distances are approximately 2.080 Å, while the O–O bond elongates from 1.240 Å in the gas phase to 1.327 Å upon adsorption, demonstrating efficient activation of molecular oxygen at Pd ensembles. In contrast, H_2O adsorption is considerably weaker (-1.57 eV), with the molecule adsorbing on top of a Pd atom at a distance of 2.643 Å.*

The calculated adsorption energies follow the order: E_{ads}(S-but/O_2) > E_{ads}(S-but/H_2O_2) > E_{ads}(S-but/H_2O). These results provide strong theoretical evidence that Pd surface atoms constitute the primary active sites for both O_2 activation and H_2O_2 adsorption during direct synthesis. Importantly, the calculations further demonstrate that thiol functionalization does not block the ability of Pd ensembles to activate O_2. Instead, butanethiol modifies the local adsorption environment by preferentially destabilizing water adsorption relative to O_2 and H_2O_2. This effect suppresses the formation of surface hydroxyl species that are known to promote H_2O_2 decomposition,[29] while simultaneously favoring H_2O_2 stabilization at the catalyst interface. Collectively, these findings support the conclusion that direct H_2O_2 synthesis predominantly occurs on Pd-rich surface ensembles of the AuPd nanoalloy, whereas the thiol layer acts as a local interfacial modifier that enhances selectivity and suppresses H_2O_2 degradation.'

3.The authors provide indirect evidence for the existence of the gas plastron based on the amount of bubble generation and confocal microscopy observations. However, key metrics such as the plastron thickness, stability, and its temporal evolution have not been quantitatively established. It would be highly beneficial to further characterize the dynamic formation process and long-term stability of the plastron, e.g., via in situ AFM or other suitable operando/interfacial probing techniques, to strengthen the central arguments of the manuscript.

We thank the reviewer for this thoughtful and constructive suggestion. We fully agree that direct, quantitative characterization of the plastron thickness, stability, and temporal evolution would provide valuable mechanistic insight. We would like to clarify however that the evidence presented in our study is not solely indirect. In addition to confocal microscopy and gas-release visualization, we quantitatively evaluated both the plastron thickness and its temporal stability using experimentally measurable physical parameters.

First, the plastron thickness was estimated using a buoyancy–gravity equilibrium model, as detailed in Section S1.5 of the Supplementary Information. Based on the measured flotation behavior and particle dimensions, the average plastron thickness surrounding the ~200 nm silica particles was calculated to be approximately 30 nm. This analysis provides a quantitative estimate of the gas-layer thickness under the reaction conditions.

Second, the temporal evolution and stability of the plastron were quantitatively monitored using time-dependent optical transmittance/absorbance measurements of particle dispersions (**Supplementary Figure 5g-h, now Figure 8g-h**). In this method, stable plastron formation leads to sustained dispersion of the particles in water due to the buoyancy provided by the trapped gas layer, whereas particles without a stable

plastron gradually sediment. The SH-AuPd/SiO_2-F-60 μL particles maintained nearly constant absorbance over time, indicating stable plastron retention, while the hydrophilic SH-AuPd/SiO_2 particles rapidly sedimented within approximately 30 min due to the absence of a gas layer. Furthermore, long-term measurements demonstrate that the plastron surrounding SH-AuPd/SiO_2-F-60 μL remains stable for at least 48 h (**Supplementary Figure 5h, now Figure 8h**).

We therefore believe that the combination of buoyancy-based thickness estimation, direct visualization, and quantitative time-resolved dispersion measurements provides experimentally grounded evidence for both the existence and stability of the plastron.

Regarding the reviewer's suggestion of *in-situ* AFM or related interfacial force microscopy techniques, such measurements are currently extremely challenging for this system because the catalyst consists of freely dispersed nanoparticles suspended under stirring in a dynamic gas-liquid environment. Conventional AFM techniques generally require immobilized flat interfaces and cannot reliably track nanoscale gas layers surrounding mobile colloidal particles under pressurized three-phase reaction conditions. To the best of our knowledge, nanometer-resolution operando AFM characterization of transient plastrons on freely diffusing catalytic nanoparticles has not yet been demonstrated in comparable systems.

For clarity, the section 'Experimental validation of plastron formation' in the manuscript has been rewritten as follows:

'Experimental validation and quantification of plastron formation. *To investigate plastron formation, different hydrophobic catalyst dispersions were prepared in pure water by ultrasonication using a pressure-resistant tube (**Supplementary Figure 1d & Movie 1**). The sealed tube was saturated with a 4% H_2:air mixture at 3 bar, followed by depressurization. Only a few bubbles are generated in a control experiment without the catalyst and when using the hydrophilic SH-AuPd/SiO_2 catalyst (**Figure 2a1,a2**). In contrast, a dispersion containing hydrophobic SH-AuPd/SiO_2-F-60μL particles shows a dramatic increase in bubble generation immediately after depressurization (**Figure 2a3**). This behavior is attributed to the release of gas trapped in plastrons (gas layers, **Supplementary Figure 1e**) formed on the surface of SH-AuPd/SiO_2-F-60μL, which expand and detach as visible bubbles upon pressure release.*

Beyond qualitative visualization, the plastron thickness was quantitatively estimated using a buoyancy-gravity equilibrium model (Supplementary Section S1.5). Based on the measured flotation behavior of the particles and the density difference between gas and liquid phases, the average plastron thickness surrounding the 200 nm silica particles was calculated to be approximately 30 nm. This thickness is consistent with a stable nanoscale gas layer capable of locally enriching dissolved reactant gases at the catalyst interface.

*The temporal evolution and stability of the plastron were further quantified by monitoring the light transmittance (turbidity) of catalyst suspensions over time (**Supplementary Figure 8d**). The SH-AuPd/SiO_2-F-60 μL particles maintain nearly constant turbidity over at least 30 min, demonstrating persistent suspension stability associated with stable plastron retention. In contrast, the hydrophilic SH-AuPd/SiO_2 particles progressively sediment within 30 min due to the absence of a stabilizing gas layer. Plastron stability was quantified as the ratio between the absorbance after 30 min and the initial absorbance, showing a clear positive correlation with particle hydrophobicity (**Supplementary Figure 8g,h**). Zeta potential measurements revealed lower negative surface charge for SH-AuPd/SiO_2 than SH-AuPd/SiO_2-F-60μL (-36 mV vs -24 mV) (**Supplementary Figure ~~5f~~8e**). This finding indicates that plastron formation, rather than electrostatic repulsion, plays a dominant role in maintaining dispersion stability in aqueous media. To investigate the relationship between hydrophobicity and plastron stability, two additional catalysts were synthesized, designated as SH-AuPd/SiO_2-F-15 μL and SH-AuPd/SiO_2-F-45 μL. The corresponding contact angle images are provided in **Supplementary Figure 4b and 4d**. The correlation between hydrophobicity and plastron stability is illustrated in **Supplementary Figure 8f**.*

*SH-AuPd/SiO_2-F-30μL generate~~d~~ fewer bubbles than SH-AuPd/SiO_2-F-60μL, which dissipate within 8 s, consistent with its lower hydrophobicity and reduced plastron stability. ~~This reduced bubble release is consistent with the lower hydrophobicity.~~ The SH-AuPd/SiO_2-F-120 μL formulation, despite exhibiting the highest contact angle ~~being the most hydrophobic catalyst~~, also ~~exhibits~~ shows less bubble release~~, as~~ because the particles float~~ed~~ ~~on~~ at the air-water interface ~~water surface~~, ~~thus~~ suppressing effective plastron formation around suspended particles (**Supplementary Figure ~~5~~8a~~,b~~-c**).*

*~~The SH-AuPd/SiO_2-F-60μL showed the highest turbidity and suspension stability in water, which was attributed to plastron formation promoting suspension rather than sedimentation (**Supplementary Figure 5d,e**).~~ ~~Zeta potential measurements revealed lower negative surface charge for SH-AuPd/SiO_2 than SH-AuPd/SiO_2-F-60μL (-36 mV vs -24 mV) (**Supplementary Figure 5f5e**). This finding indicates that plastron formation, rather than electrostatic repulsion, plays a dominant role in maintaining dispersion stability in aqueous media.~~*

*Confocal microscopy was additionally used to directly visualize plastron formation on catalyst surfaces.[29] Hydrophilic and hydrophobic silica particles – modified with Rhodamine B (red fluorescence) to yield SiO_2-RB and SiO_2-F-RB, respectively – were dispersed into an aqueous solution of fluorescein isothiocyanate (FITC, green fluorescence). A distinct black halo devoid of fluorescence was observed surrounding the SiO_2-F-RB particles (**Figure***

*2b1), indicating the presence of an interfacial air layer ~~(plastron)~~ between the red fluorescent particle (hydrophobic) and surrounding aqueous phase (green FITC solution). In contrast, SiO_2-RB particles (hydrophilic) display yellow fluorescence resulting from the overlayp of red (Rhodamine B) and green (FITC) emissions against a non-luminous background (**Figure 2b2**),confirming direct contact between the particle surface and the surrounding aqueous solution with no plastron formation. The intensity profile across the scan line (**Figure 2b3**) shows maximum red intensity at the particle center and a gradual decrease toward the edges, inversely correlated with green intensity. ~~This confirms that hydrophilic SiO_2-RB particles absorb FITC from the solution, and no air layer forms at the interface.~~*

While advanced operando methods such as in-situ AFM could in principle provide additional interfacial information, their implementation under the present reaction conditions remains experimentally challenging because freely diffusing nanoparticles suspended in bulk liquid cannot currently be tracked with sufficient spatial and temporal resolution under reactive gas-liquid-solid conditions. Therefore, the combined use of buoyancy-based thickness estimation, time-resolved turbidity measurements, bubble-release analysis, and confocal microscopy provides a robust and experimentally accessible characterization of plastron formation, stability, and temporal evolution in the present catalytic system.'

4.In the manuscript, the authors tune the hydrophobicity of the catalysts by varying the PFOTES loading and attempt to correlate the hydrophobic nature of the catalyst with the ease of plastron formation and the resulting catalytic performance. However, excessively hydrophobic catalysts tend to float in the electrolyte, which suppresses plastron formation and leads to performance deactivation, thereby complicating the proposed correlation. It would therefore be valuable to design an expanded set of catalysts with graded hydrophobicity to systematically establish a correlation model linking contact angle, plastron stability, and catalytic performance.

We thank the reviewer for this insightful remark. Two additional catalysts were synthesized, namely SH-AuPd/SiO_2-F-15 µL and SH-AuPd/SiO_2-F-45 µL. The corresponding contact angle measurements are shown in **Supplementary Figure 4b** and **4d**. The light absorbance of the particle dispersions was employed as an indicator of plastron stability. Hydrophilic particles, which do not form plastrons, gradually sediment on the bottom over time. Particles with intermediate hydrophobicity (SH-AuPd/SiO_2-F-15 µL to 60 µL), capable of sustaining a plastron, remain dispersed in water and consequently exhibit high light absorbance. In contrast, highly hydrophobic particles (SH-AuPd/SiO_2-F-120 µL) float at the water surface, resulting in the lowest light absorbance. The resulting correlation model is presented in **Supplementary Figure 9f**. Besides, two additional particles, SH-SH-AuPd/SiO_2-F-15µL and SH-AuPd/SiO_2-F-45µL, with different degrees of hydrophobicity, afford a H_2O_2 concentration of 290 µM and 750 µM after 0.5 h, respectively, in agreement with the observed trend (**Supplementary Figure 9f**).

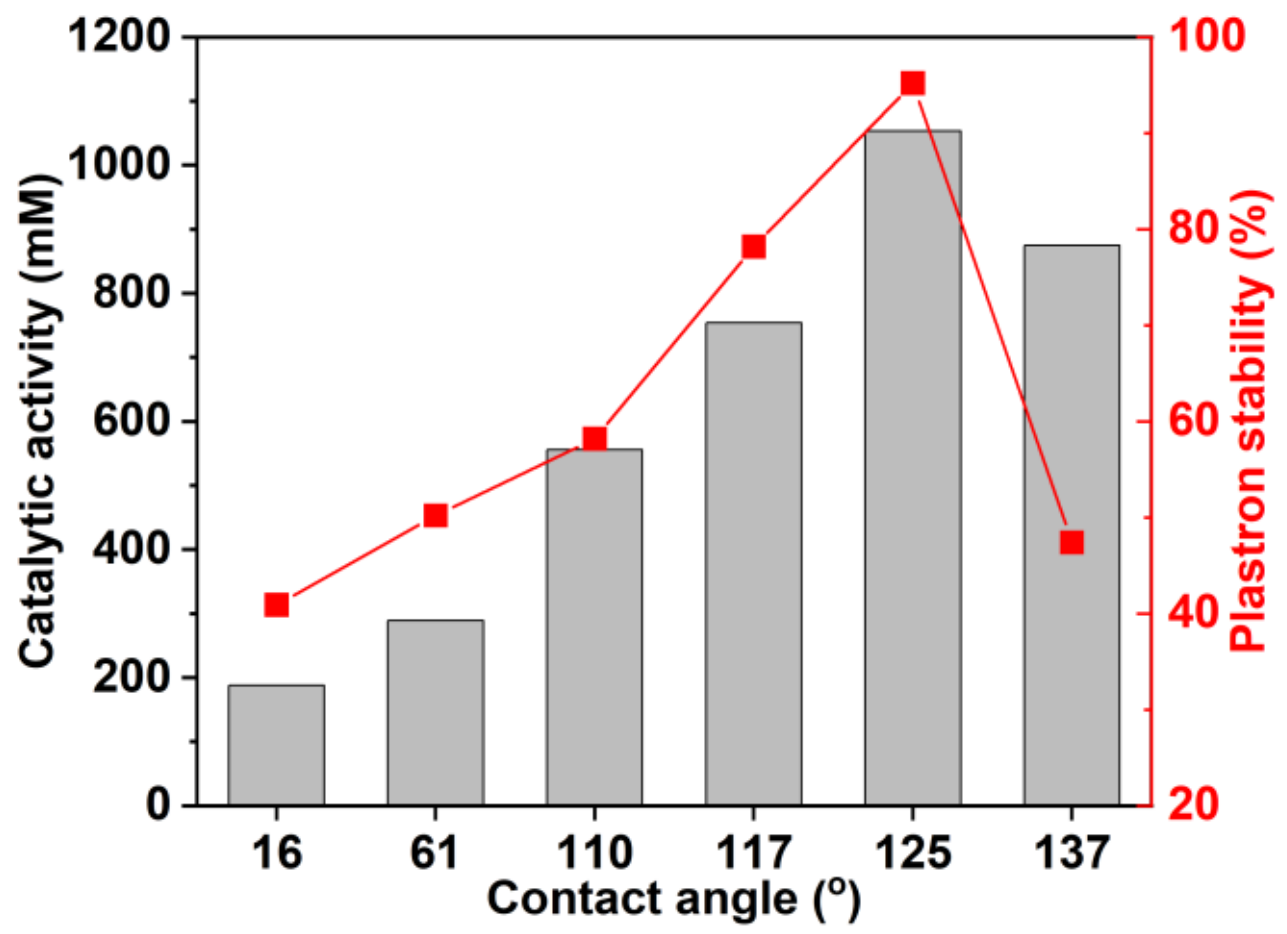

Supplementary Figure ~~5~~9. Turbidity and zeta potential data. f, Correlation between the catalytic activity, plastron stability, and hydrophobicity of the different particles. Particles: 16°: SH-AuPd/SiO_2, 61°: SH-AuPd/SiO_2-F-15µL, 110°: SH-AuPd/SiO_2-F-30µL, 117°: SH-AuPd/SiO_2-F-45µL, 125°: SH-AuPd/SiO_2-F-60µL, 137°: SH-AuPd/SiO_2-F-120µL.

For clarity, the following text has been added to the Methods section:

*"**Plastron stability tests.** The light absorbance of particle dispersions was employed as a quantitative descriptor of plastron stability. Specifically, 2.5 mg of each particle sample was dispersed in 5 mL of water, and 3 mL of the resulting suspension was transferred to a UV-Vis cuvette for absorbance measurement at 600 nm. The corresponding absorbance values are presented in **Supplementary Figure 9f**. Plastron stability was defined as the ratio of the absorbance at 30 min to that at 0 min, expressed as a percentage. The correlation between hydrophobicity and plastron stability is shown in **Supplementary Figure 9f**."*

5. The statement in the manuscript: "The H_2O_2 concentration increased …… to 560 µM and 1050 µM for SH-AuPd/SiO_2-F-60 µL and SH-AuPd/SiO_2-F-30 µL, respectively." appears to be inconsistent with the labeling/order shown in Figure 3a1-a3. Please verify and correct the correspondence between the sample labels and the reported H_2O_2 concentrations to avoid confusion.

We thank the reviewer for the remark. These values correspond to SH-AuPd/SiO_2-F-30 µL and SH-AuPd/SiO_2-F-60 µL (inverse order). This has been corrected in the section 'Catalytic performance under plastron formation').

6.The manuscript reports a pronounced bell-shaped dependence of the H_2O_2 concentration on catalyst loading for the SH-AuPd/SiO_2-F-60 µL catalyst, which is attributed to "competitive H_2O_2 decomposition activity." However, this explanation is currently based on qualitative reasoning and lacks direct experimental support. It is recommended that the authors provide additional evidence, such as H_2O_2 decomposition kinetics measurements or other relevant validation experiments, and discuss alternative physical factors that may contribute to the observed bell-shaped trend, in order to strengthen the robustness of the proposed mechanistic interpretation.

We thank the reviewer for this constructive suggestion. To provide additional direct evidence of the proposed competitive H_2O_2 degradation activity, we conducted H_2O_2 degradation experiments at variable catalyst loadings under identical reaction conditions. As shown in the right Y-axis of the new **Supplementary Figure 11a**, the H_2O_2 degradation rate increases monotonically with catalyst loading. These results support our interpretation that the increased degradation rate competes with H_2O_2 formation at high catalyst loading, supporting the observed bell-shaped curve.

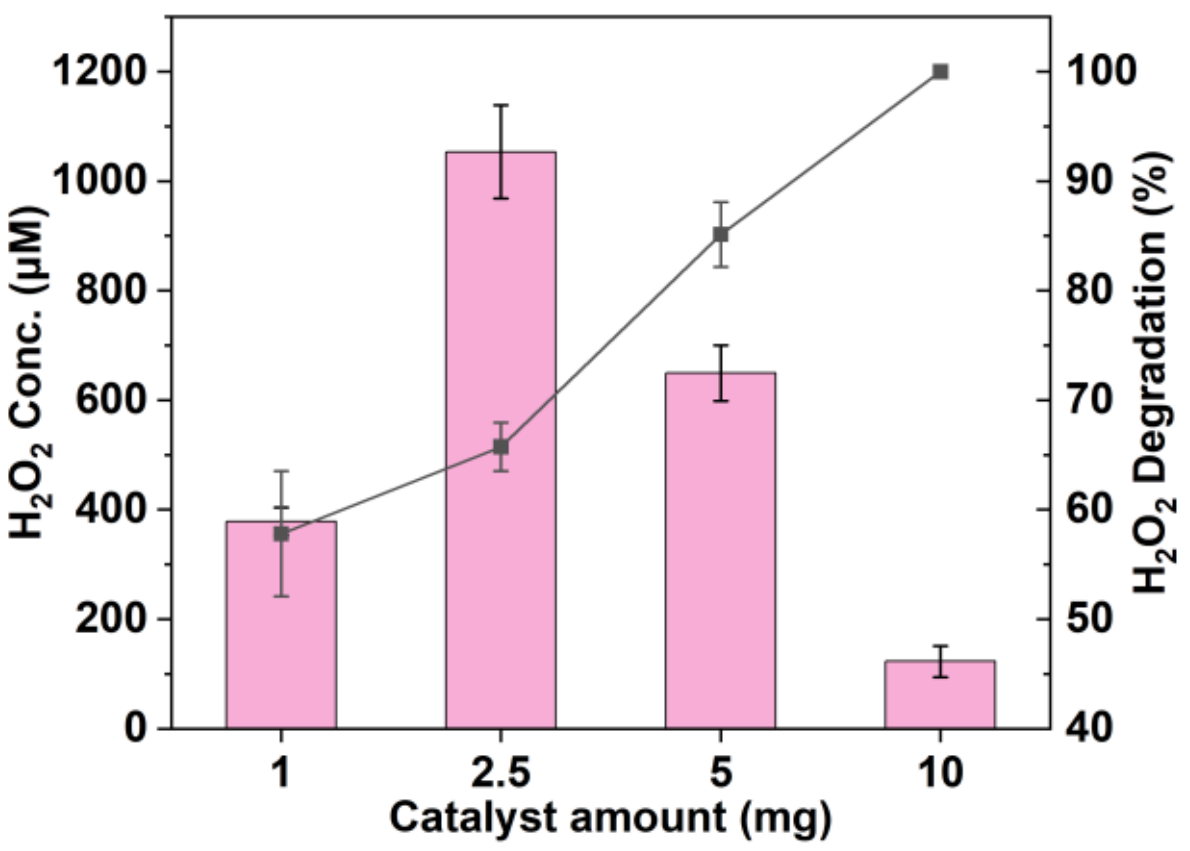

Supplementary Figure 11a. Evolution of the H_2O_2 concentration for SH-AuPd/SiO_2-F-60µL particles at variable loading. Reaction conditions for H_2O_2 production (left axis): 5 mL of deionized water, H_2:Air=4%, 3 bar, 22 °C, 1500 rpm, 30 min. Reaction conditions for H_2O_2 degradation (right axis): 5 mL 2500 µM H_2O_2 solution, H_2:N_2=4%, 3 bar, 22 °C, 1500 rpm, 30 min,

7.The enhanced selectivity is attributed to the "exclusion of H_2O_2 by the gas plastron, which reduces its contact with the catalyst surface." However, no direct evidence is provided to demonstrate the interfacial exclusion of H_2O_2 (e.g., interfacial concentration measurements, adsorption energetics, or in situ/operando characterization). Moreover,

thiol modification itself can block Pd sites and suppress H_2O_2 decomposition, which is intertwined with the proposed plastron effect, making it difficult to disentangle the respective contributions of surface site blocking and gas-plastron-induced interfacial regulation. Clarification through targeted control experiments or additional mechanistic evidence would be necessary to deconvolute these effects.

We thank the reviewer for this insightful and important comment. We agree that distinguishing the respective contributions of thiol site modification and gas-plastron-induced interfacial regulation is critical for understanding the origin of the enhanced H_2O_2 selectivity. To clarify this point, we performed additional DFT calculations to evaluate the adsorption energetics of relevant species on the thiol-functionalized AuPd surface. In line with the answer to question 2, the calculated adsorption energies follow the order:

$$E_{ads}(\text{S-but, } O_2) > E_{ads}(\text{S-but, } H_2O_2) > E_{ads}(\text{S-but, } H_2O).$$

These results indicate that the thiol-modified AuPd surface thermodynamically favors the adsorption of O_2 and H_2O_2 over H_2O. Importantly, water adsorption is significantly weakened in the presence of butanethiol ligands. Since adsorbed water and surface hydroxyl species are known to promote H_2O_2 decomposition through O–O bond activation and proton-transfer pathways (Plauck *et al*, *Proc. Natl. Acad. Sci. USA*, **2016**, *113*, E1973-E1982), the reduced water affinity induced by the hydrophobic interface is expected to suppress H_2O_2 degradation reactions over AuPd.

Accordingly, we have revised our mechanistic interpretation. Rather than proposing a direct exclusion of H_2O_2 from the catalyst surface, the computational results support a mechanism in which the hydrophobic thiol environment preferentially excludes water from the vicinity of the active sites while maintaining favorable accessibility for gaseous reactants and H_2O_2. This interfacial regulation decreases the probability of water-assisted H_2O_2 decomposition pathways and thereby improves selectivity.

To further disentangle the contributions of butanethiol functionalization and plastron formation, we compared catalysts with different hydrophobicity while maintaining the same AuPd loading and thiol treatment. Catalysts exhibiting stable plastron formation consistently showed higher H_2O_2 productivity and lower degradation activity than the corresponding non-plastron systems. In addition, the temperature-dependent kinetic analysis revealed that the plastron system transitions from gas-liquid mass-transfer limitation to kinetically controlled behavior, further supporting the distinct mechanistic role of the gas-plastron interface beyond simple site blocking by thiols.

We acknowledge that direct *operando* measurements of interfacial concentrations in dispersed plastron systems remain experimentally challenging. Nevertheless, the combined adsorption-energy calculations, degradation experiments, hydrophobicity-dependent catalytic trends, and kinetic analysis collectively support the conclusion that both thiol-induced surface modification and plastron-assisted interfacial regulation contribute cooperatively to the enhanced H_2O_2 selectivity and that butanethiol does not block the active sites.

In line with the answer to question 2, the following section has been added after 'Characterisation of hydrophobic catalysts':

'Computational investigation of the active sites for H_2O_2 synthesis. *To further clarify the nature of the active sites involved in direct H_2O_2 synthesis, density functional theory (DFT) calculations were performed on the AuPd(111) surface to investigate the adsorption behavior of H_2O_2, O_2, H_2O, and butanethiol (S-but). The calculations reveal that H_2O_2 adsorbs most favorably on top of Pd atoms with an adsorption energy of -1.03 eV and a Pd–O distance of 2.412 Å (**Supplementary Figure 6**). Upon adsorption, the O–O bond length increases slightly from 1.483 Å in the gas phase to 1.488 Å at the AuPd surface, accompanied by the appearance of new vibrational modes at 500 and 3500 cm^{-1}, indicating direct interaction between H_2O_2 and Pd surface atoms.*

*We next investigated the co-adsorption of butanethiol and H_2O_2 on AuPd(111). The calculations show that butanethiol preferentially adsorbs at a Pd–Pd bridge configuration, while H_2O_2 remains adsorbed on top of a Pd atom, resulting in a total adsorption energy of -2.58 eV (**Supplementary Figure 7**). The adsorbed thiolate forms two Pd–S interactions with bond distances of 2.308 and 2.317 Å. Interestingly, the Pd–O distance for H_2O_2 decreases from 2.412 Å in the isolated adsorption state to 2.400 Å in the co-adsorbed configuration, suggesting a strengthened metal–adsorbate interaction through synergistic effects between the two adsorbates.*

To further probe the active sites involved in the reaction pathway, we compared the co-adsorption of butanethiol

*with O_2 and H_2O on AuPd(111). O_2 adsorbs preferentially on Pd-rich regions, forming a bridge configuration between neighboring Pd atoms with an adsorption energy of -3.34 eV (**Supplementary Figure 8**). The Pd–O bond distances are approximately 2.080 Å, while the O–O bond elongates from 1.240 Å in the gas phase to 1.327 Å upon adsorption, demonstrating efficient activation of molecular oxygen at Pd ensembles. In contrast, H_2O adsorption is considerably weaker (-1.57 eV), with the molecule adsorbing on top of a Pd atom at a distance of 2.643 Å.*

The calculated adsorption energies follow the order: E_{ads}(S-but/O_2) > E_{ads}(S-but/H_2O_2) > E_{ads}(S-but/H_2O). These results provide strong theoretical evidence that Pd surface atoms constitute the primary active sites for both O_2 activation and H_2O_2 adsorption during direct synthesis. Importantly, the calculations further demonstrate that thiol functionalization does not block the ability of Pd ensembles to activate O_2. Instead, butanethiol modifies the local adsorption environment by preferentially destabilizing water adsorption relative to O_2 and H_2O_2. This effect suppresses the formation of surface hydroxyl species that are known to promote H_2O_2 decomposition,[29] while simultaneously favoring H_2O_2 stabilization at the catalyst interface. Collectively, these findings support the conclusion that direct H_2O_2 synthesis predominantly occurs on Pd-rich surface ensembles of the AuPd nanoalloy, whereas the thiol layer acts as a local interfacial modifier that enhances selectivity and suppresses H_2O_2 degradation.'

8.The manuscript reports only two cycling runs, and a noticeable activity decay is observed. This raise concerns regarding whether the gas plastron can be sustainably maintained during long-term operation and whether the hydrophobic layer may be degraded by reactants and/or products. It is recommended that the authors extend the reaction duration to several hours and monitor both the plastron (gas-shield) stability and the evolution of catalytic activity over time. In addition, surface analyses of the post-reaction catalysts (e.g., XPS and contact-angle measurements) are suggested to evaluate the integrity of the hydrophobic layer and possible surface energy changes after operation.

In response to the reviewer's comment, the long-term plastron stability of SH-AuPd/SiO_2-F-60 μL particles was further investigated using the light absorbance method (see new **Supplementary Figure 9h**). The results indicate that the plastron remains stable for at least 48 h. To assess potential surface modifications after catalysis, post-reaction characterizations were performed. XPS analysis of the used catalysts is presented in the new **Supplementary Figure 3g-i**, and the corresponding post-reaction water contact angle measurements are shown in **Supplementary Figure 4h**. Both analyses reveal no significant differences compared to the fresh samples, suggesting that the surface chemical composition and hydrophobicity are largely preserved. In addition, the post-reaction solutions were examined by ^{1}H NMR spectroscopy to detect possible leaching of fluorinated hydrophobic chains (**Supplementary Figure 9j**). No signals attributable to fluorinated chain leaching were observed.

Collectively, these results confirm that the hydrophobic layer remains intact during the reaction. The observed activity decay is therefore attributed to the leaching of thiol groups, as the catalytic activity can be restored upon re-doping the catalyst with butanethiol, as shown in the **Figure 3f**.

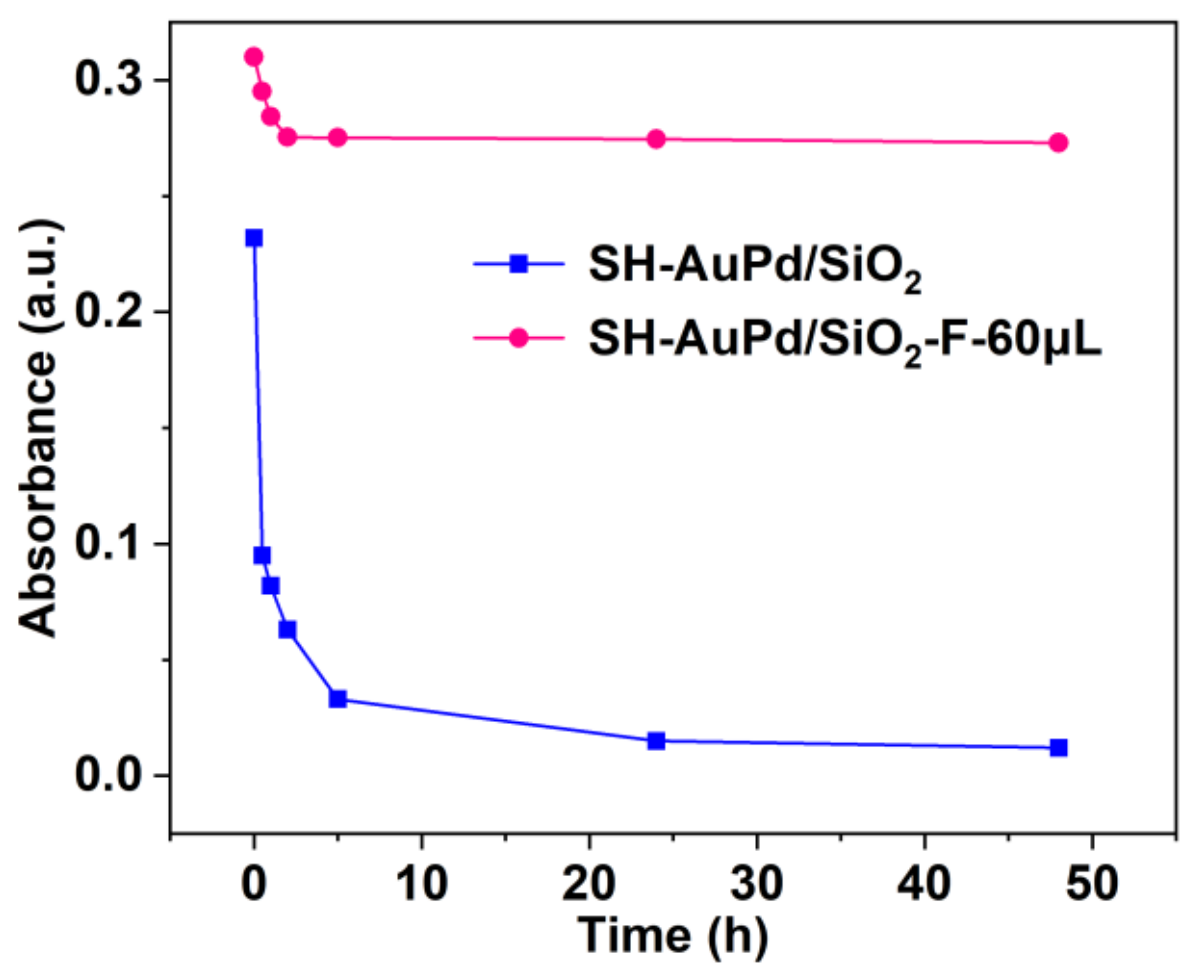

Supplementary Figure 9h. Plastron stability of the SH-AuPd/SiO_2-F-60μL particle.

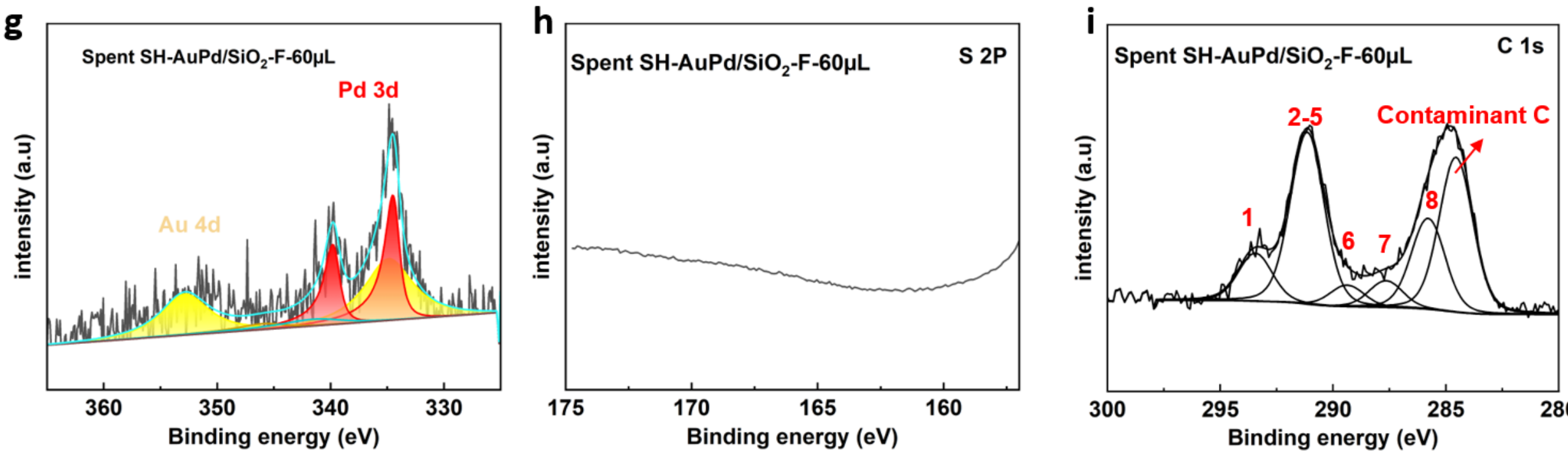

Supplementary Figure 3g. Au(4d) and Pd(3d); h, S 2p, i,C(1s) of the spent SH-AuPd/SiO_2-F-60 μL.

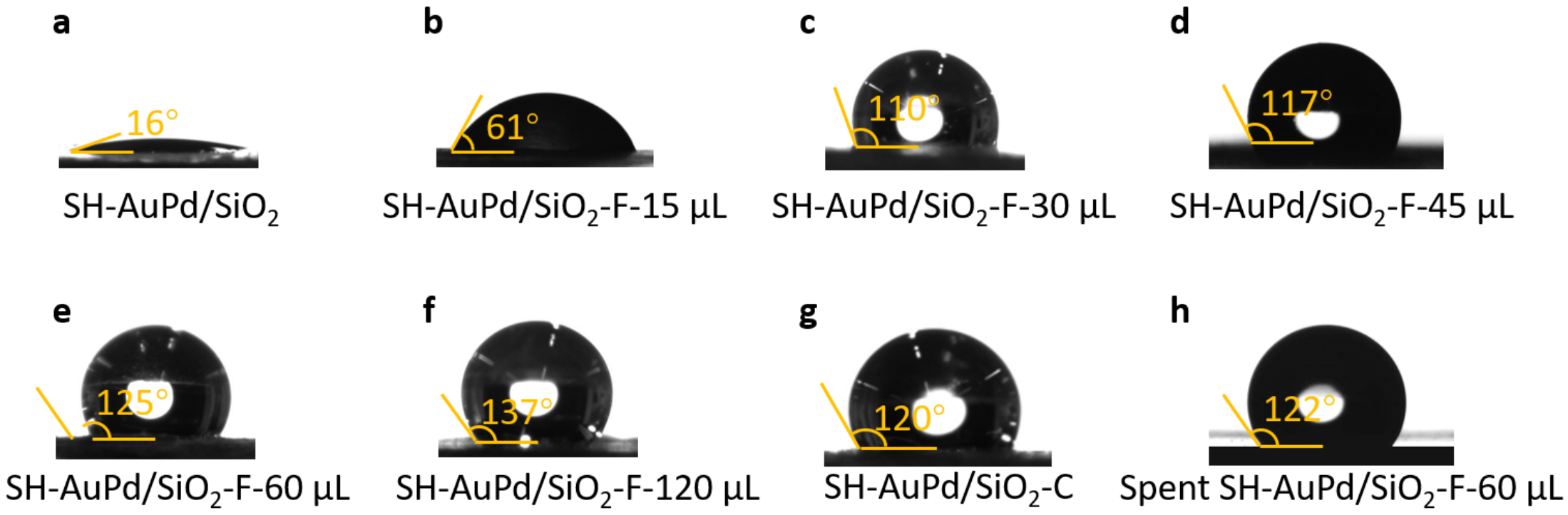

Supplementary Figure 4. Contact angle of water with pellets based on a, SH-AuPd/SiO_2; b, SH-AuPd/SiO_2-F-15 μL;c, SH-AuPd/SiO_2-F-30 μL; d, SH-AuPd/SiO_2-F-45 μL; e, SH-AuPd/SiO_2-F-60 μL; f, SH-AuPd/SiO_2-F-120 μL; g, SH-AuPd/SiO_2-C-60 μL; and h, spent SH-AuPd/SiO_2-C-60 μL.

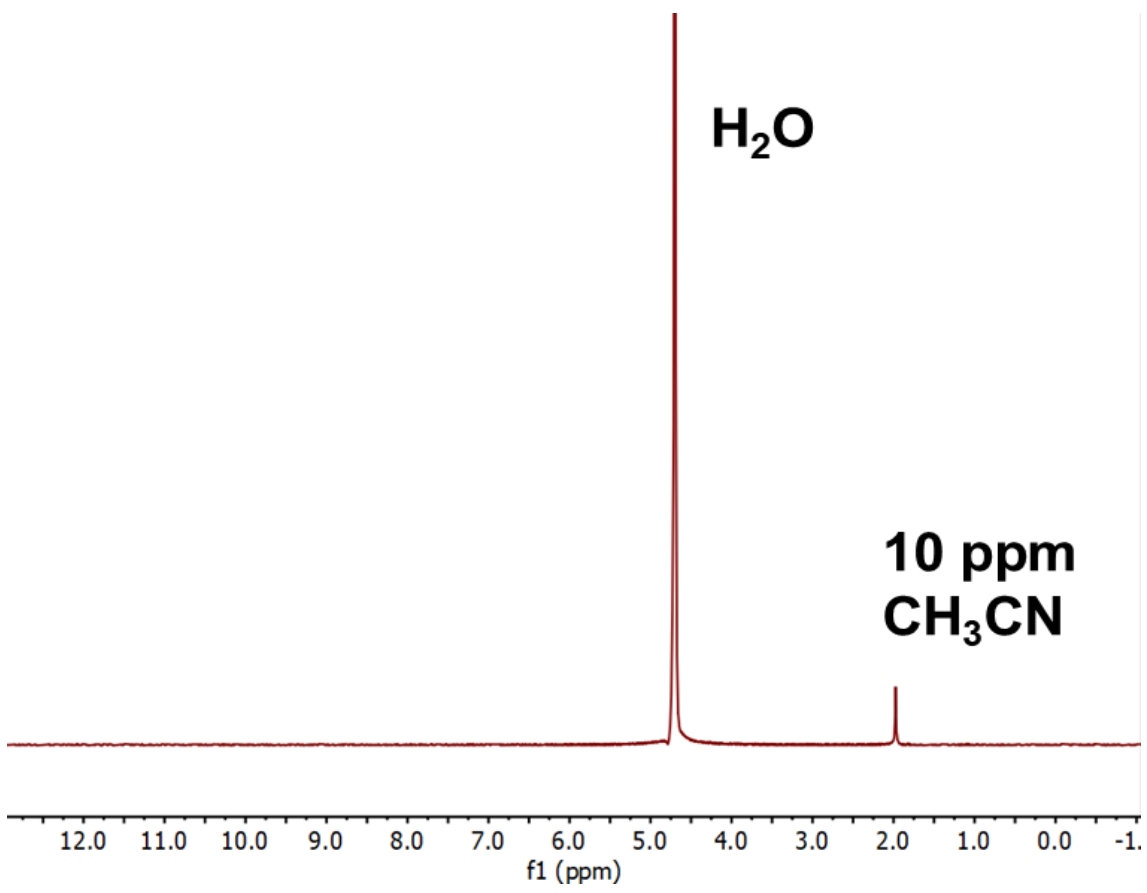

Supplementary Figure 9j. ^{1}H NMR spectrum of the filtrate after solution. Experimental conditions: 10 ppm CH_3CN was used as the internal standard.

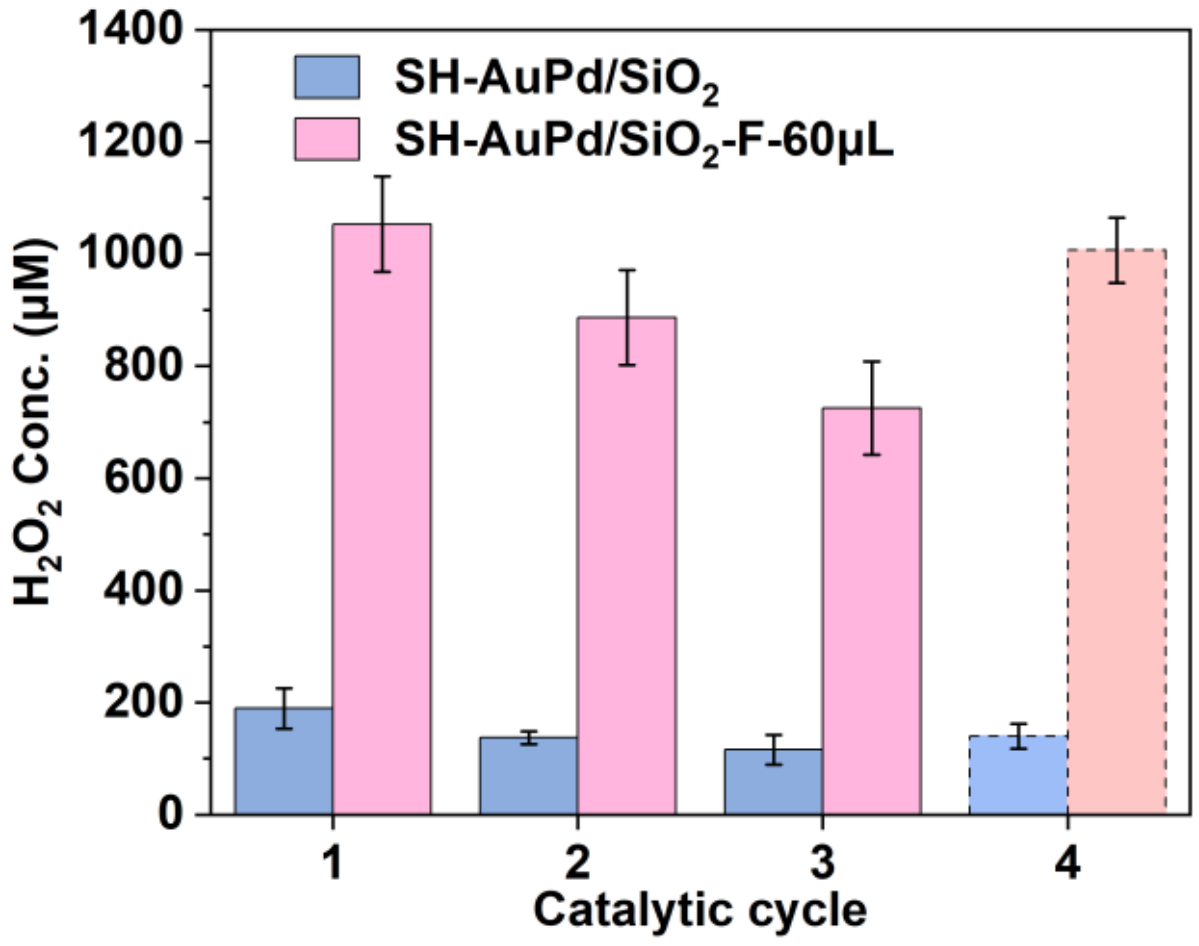

Figure 3f. Recyclability and reuse of SH-AuPd/SiO_2 and SH-AuPd/SiO_2-F-60µL particles. Reaction conditions: H_2:Air = 4%, 3 bar, 22 °C, 30 min, 2.5 mg catalyst, 5 mL of deionized water, 1500 rpm. run 4 was carried out after re-doping the spent catalyst from run 3 with the same concentration of butanethiol as fresh catalyst.

9.The identification of the active sites and their mechanistic roles remain unclear. The manuscript emphasizes the formation of self-assembled monolayers (SAMs) of butanethiol to modulate surface properties and reports that CO-DRIFTS shows no CO adsorption after thiol treatment, suggesting Pd sites are blocked. Yet, the reaction proceeds efficiently, raising critical questions: What are the true active sites? If Pd is fully covered by thiols, how is H_2 activation occurring? Could there be exposed Au-Pd interfacial sites or defect sites contributing to activity? Do the hydrophobic chains (PFOTES/OTES) exclusively modify the SiO_2 support, or do they partially cover the metal nanoparticles as well? It is recommended that additional experiments be conducted to probe the chemical state of the metals under reaction conditions, and/or that theoretical studies be provided to elucidate the mechanistic role of the metal-support interface during the reaction.

We thank the reviewer for this important and insightful comment. We agree that clarifying the nature of the active sites and the respective roles of thiol ligands and hydrophobic modifiers is essential for understanding the catalytic mechanism.

To elucidate the nature of active sites, we performed DFT calculations on the AuPd(111) alloy surface. The calculations show that butanethiol preferentially adsorbs in a Pd–Pd bridge configuration with Pd–S distances of approximately 2.31 Å, while O_2 adsorbs strongly on neighboring Pd sites and remains significantly activated, as evidenced by elongation of the O–O bond from 1.240 Å in the gas phase to 1.327 Å upon adsorption. Importantly, the co-adsorption calculations demonstrate that thiol adsorption does not fully suppress O_2 activation at the AuPd surface. Instead, the sulfur ligands modify the local adsorption environment while preserving adjacent catalytically active Pd-containing ensembles.

Furthermore, the calculations indicate that H_2O_2 preferentially adsorbs on Pd top sites with moderate adsorption strength, whereas water adsorption is comparatively weaker in the presence of thiol ligands. These results support a mechanism in which partially thiol-decorated Pd sites and Au–Pd interfacial regions cooperatively participate in H_2 and O_2 activation while the surrounding hydrophobic environment suppresses water-induced H_2O_2 decomposition pathways.

The preferential adsorption of sulfur on bridged Pd sites is consistent with the disappearance of bridged CO adsorption bands after thiol functionalization in SH-AuPd/SiO_2 and SH-AuPd/SiO_2-F-60 µL. Besides, the fact that linear CO adsorption bands vanish can be explained by the presence of electron-depleted isolated Pd sites surrounded by Au and adsorbed butanethiol discouraging Pd $\rightarrow$ CO π-back donation.

In AuPd/SiO_2 particles, silane molecules primarily react with the silica surface, not with the gold-palladium nanoparticles. This is because SiO_2 has abundant surface silanol groups (Si–OH), which readily undergo

condensation with hydrolyzed silanes to form stable Si-O-Si bonds. In contrast, AuPd nanoparticles have mostly metallic surfaces with few reactive hydroxyl groups, so silanes do not form strong covalent bonds with them under normal conditions. This interpretation is supported by the preservation of the Au and Pd XPS signals after fluorosilane treatment, as well as the maintained catalytic activity.

To further probe catalyst stability and surface chemistry after reaction, we additionally performed post-reaction XPS characterization, contact-angle measurements, zeta-potential analysis, and particle-size-distribution measurements. These analyses show that the fluorinated hydrophobic layer remains largely intact after catalysis, while the catalytic deactivation is primarily associated with partial thiol loss rather than collapse of the hydrophobic interface. The catalytic activity could be restored after re-doping the catalyst with butanethiol, further supporting this interpretation.

For clarity, the corresponding discussion has been expanded in the revised manuscript (see text in the answer to question 7).

Answers to the comments of Reviewer 2

This manuscript presents a well-designed study on the use of gas layer trapped on a hydrophobic catalyst (plastron) to enhance direct H_2O_2 synthesis under mild conditions. The work combining catalyst synthesis, advanced characterization, simulation, and catalytic testing is commendable, which offers significant theoretical supports toward the development of direct H_2O_2 synthesis. However, some issues still need to be addressed before the further consideration of this manuscript.

We thank the reviewer for the warm consideration of our study.

1. While confocal microscopy and 2D numerical simulations suggest visualize plastron formation on catalyst surface, in-situ Raman/IR spectroscopy techniques are suggested to be further employed to directly analyze the reaction sites and the processes of direct H_2O_2 synthesis.

We thank the reviewer for this valuable suggestion. We agree that *in-situ* vibrational spectroscopy would provide additional insight into the reaction environment and interfacial processes associated with plastron-assisted H_2O_2 synthesis.

In the present work, we employed a combination of confocal microscopy, gas-release visualization, wettability analysis, turbidity evolution measurements, and simulations to characterize the formation and stability of the gas plastron. In addition, we performed extensive DFT calculations combined with simulated IR spectra to investigate the adsorption configurations of butanethiol, O_2, H_2O, and H_2O_2 on the AuPd alloy surface. These calculations provide molecular-level insight into the interaction between the catalyst surface and reaction intermediates.

Experimentally, implementing *in-situ* Raman or IR spectroscopy under the present reaction conditions is highly challenging because the catalytic system consists of freely dispersed nanoparticles in an aqueous three-phase environment under pressurized gas conditions. Under these dynamic conditions, obtaining stable and spatially resolved vibrational signals from transient interfacial species is currently difficult. Moreover, the low concentration of reaction intermediates and the strong scattering from suspended particles significantly complicate operando spectral acquisition.

Nevertheless, to further strengthen the mechanistic interpretation, we incorporated additional theoretical analysis of adsorption energetics and vibrational signatures. The calculations show that O_2 adsorption remains favorable on partially thiol-covered AuPd surfaces and that the hydrophobic environment weakens competitive water adsorption. These results support the proposed role of the plastron in facilitating gas accessibility while suppressing H_2O_2 decomposition pathways.

2. Please perform a more detailed kinetic study at different temperatures to determine the apparent activation energy (Ea) for H_2O_2 formation with and without the plastron. A significant difference in Ea would provide strong evidence for a change in the rate-limiting step or mechanism.

We thank the reviewer for this valuable suggestion. We agree that a more comprehensive kinetic investigation over a range of temperatures would strengthen our understanding of the plastron system. Accordingly, we conducted temperature-dependent experiments to determine the apparent activation energy (Ea) for H_2O_2 formation in both the plastron and non-plastron systems.

The apparent activation energies were obtained from Arrhenius plots based on the initial H_2O_2 formation rates measured at 273, 293, 313, and 333 K. For the SH-AuPd/SiO_2-F-60 μL catalyzed plastron system, the H_2O_2 production rate increased with the temperature (**Supplementary Figure 11e**), indicating kinetically controlled behavior. In contrast, for SH-AuPd/SiO_2 without plastron, the H_2O_2 production rate remained nearly constant over the investigated temperature range (**Supplementary Figure 11f**), suggesting that the reaction is controlled by gas-liquid mass transfer rather than by intrinsic surface kinetics. To exclude the possibility that enhanced H_2O_2 degradation at elevated temperature offsets the rate of formation, we increased the stirring speed using a larger stirring bar. At these conditions, the H_2O_2 production rate increased significantly, further confirming that the reaction over SH-AuPd/SiO_2 is limited by mass transfer.

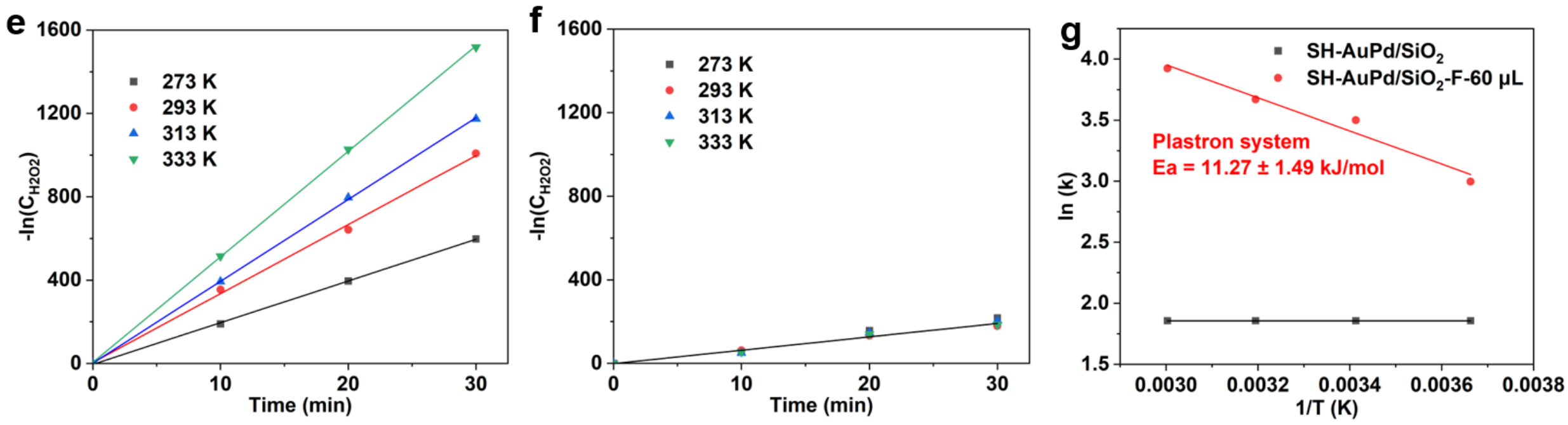

Supplementary Figure 11e. Kinetic plots of -ln(C_{H2O2}) against time for the H_2O_2 direct synthesis catalyzed by SH-AuPd/SiO_2-F-60μL with plastron. **f**, Kinetic plots of -ln(C_{H2O2}) against time for the H_2O_2 direct synthesis catalyzed by SH-AuPd/SiO_2 without plastron. **g**, Variation of TOF at time t=0 with reciprocal temperature for SH-AuPd/SiO_2-F-60μL and SH-AuPd/SiO_2.

The apparent activation energies are presented in **Supplementary Figure 11g**. The distinct difference in Ea values between the two systems demonstrates that the plastron configuration effectively reduces gas-liquid mass transfer resistance, thereby enabling intrinsic kinetic control.

For clarity, this analysis has been added to the Supplementary Information. The following text has been added to the section 'Catalytic performance under plastron formation':

*'The apparent activation energies were obtained from Arrhenius plots based on the initial H_2O_2 formation rates measured at different temperatures. For the SH-AuPd/SiO_2-F-60 μL catalyzed plastron system, the H_2O_2 production rate increased with the temperature (**Supplementary Figure 11e**), indicating a kinetically controlled behavior. In contrast, for SH-AuPd/SiO_2 without plastron, the H_2O_2 production rate remained nearly constant over the investigated temperature range (**Supplementary Figure 11f**), suggesting that the reaction is controlled by gas-liquid mass transfer rather than intrinsic surface kinetics. The corresponding apparent activation energies are presented in **Supplementary Figure 11g**. The distinct difference in Ea values between both catalysts demonstrates that the plastron configuration effectively reduces gas-liquid mass transfer resistance, thereby enabling intrinsic kinetic control'.*

3. The production rate of H_2O_2 in the recycling experiment significantly decreased. The authors discussed that the observed increase in H_2 conversion for the spent catalyst could be attributed to enhanced gas-metal interactions due to the reduced coverage of thiol groups. Please provide XPS S 2p spectra of the fresh and spent catalysts to directly demonstrate the loss of S species. In addition, the Zeta potential, hydrophobicity, particle size distribution and other aspects of the catalyst before and after reaction should also be analyzed to comprehensively investigate the reasons for the decrease in activity.

The fresh catalysts show no XPS S2p peak in the **Supplementary Figure 3a** because of the low metal loading and thiol amount. For clarity, we added the XPS S2p spectra of the spent catalyst in **Supplementary Figure 3h**.

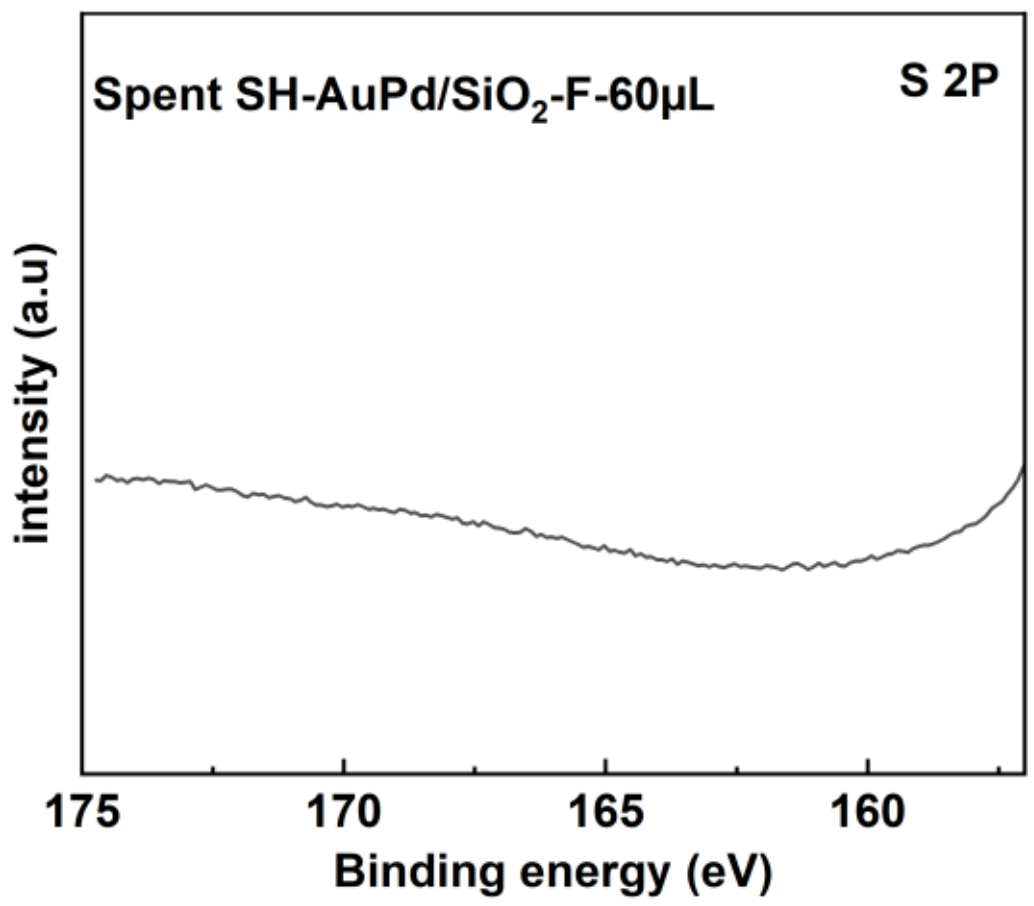

Supplementary Figure 3h. S 2p of the spent SH-AuPd/SiO_2-F-60 μL.

The contact angle, zeta potential, and particle size distribution (dynamic light scattering) were analysed after reaction in **Supplementary Figure 4h, 9e, and 2d**. They show no difference compared to the fresh catalyst. Besides, the metal nanoparticle size distribution after reaction is shown in **Supplementary Figure 2d**.

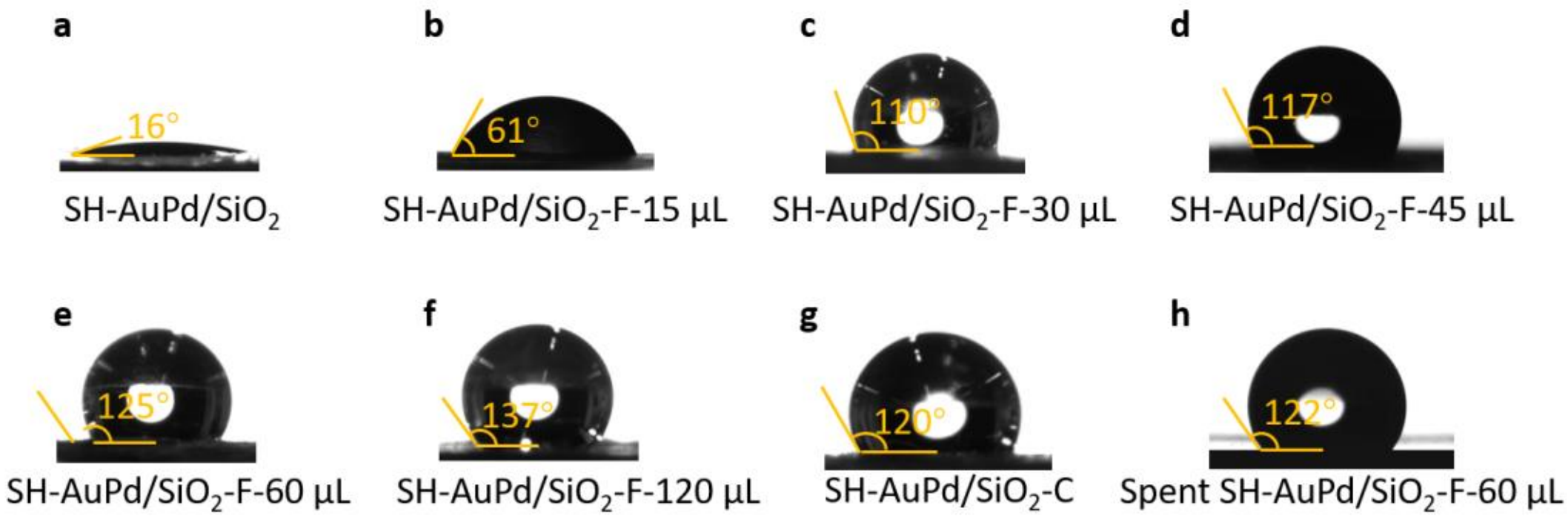

Supplementary Figure 4. Contact angle of water with pellets based on a, SH-AuPd/SiO_2; b, SH-AuPd/SiO_2-F-15 μL;c, SH-AuPd/SiO_2-F-30 μL; d, SH-AuPd/SiO_2-F-45 μL; e, SH-AuPd/SiO_2-F-60 μL; f, SH-AuPd/SiO_2-F-120 μL; g, SH-AuPd/SiO_2-C-60 μL; and h, spent SH-AuPd/SiO_2-C-60 μL.

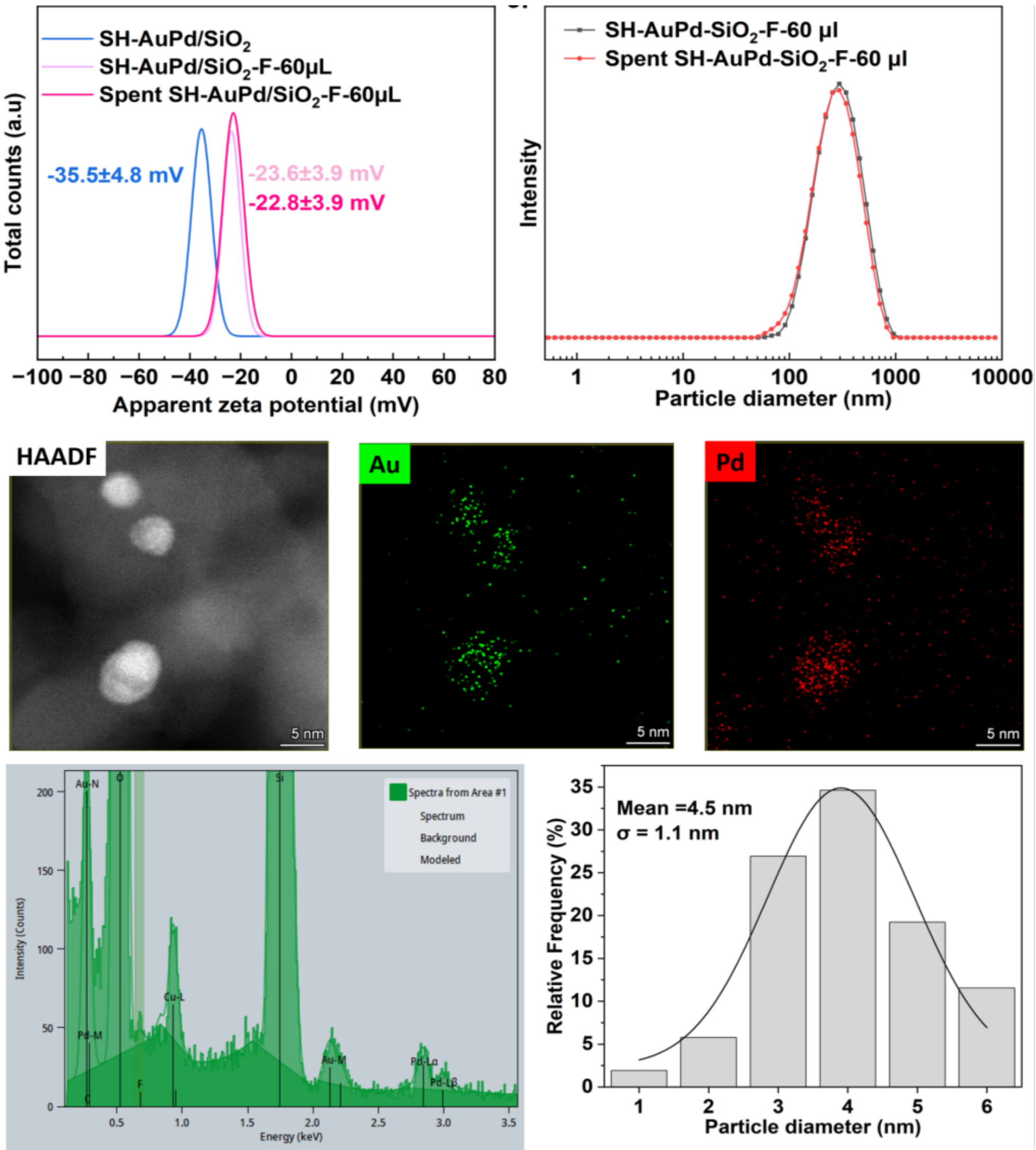

Supplementary Figure 9e (top images). Zeta potential of SH-AuPd/SiO_2, SH-AuPd/SiO_2-F-60µL, and Spent SH-AuPd/SiO_2-F-60µL dispersions in water. **2c (middle and bottom images).** Particle size distribution of spent SH-AuPd/SiO_2-F-60µL particles. Conditions: Samples were prepared using a dry deposition method onto a carbon-coated copper grid, and the grids were plasma-cleaned for 2 min.

4. Although SH-AuPd/SiO_2-F-60µL exhibits lower decomposition activity compared to the SH-AuPd/SiO_2, the H_2O_2 decomposition rate of SH-AuPd/SiO_2-F-60µL remains very high, which contradicts the fact that the hydrophobic modification significantly inhibits the decomposition process of H_2O_2. The stability of the catalyst may be an important factor influencing the decomposition of H_2O_2.

We thank the reviewer for this insightful comment. As shown in the original **Figure 3e**, SH-AuPd/SiO_2-F-60µL exhibited slightly lower H_2O_2 decomposition and degradation activity compared to SH-AuPd/SiO_2, although the activity was still high. To confirm this result, we prepared a batch of fresh catalyst and repeated the catalytic tests under identical conditions. The results obtained with the freshly prepared catalyst show a decrease in both H_2O_2 decomposition and degradation rates. We therefore attribute the previously observed high activities to potential aging effects in the earlier catalyst sample, as it was prepared several months prior to testing, which may have affected its catalytic performance. Accordingly, **Figure 3e** has been replaced with data obtained from the freshly prepared catalyst.

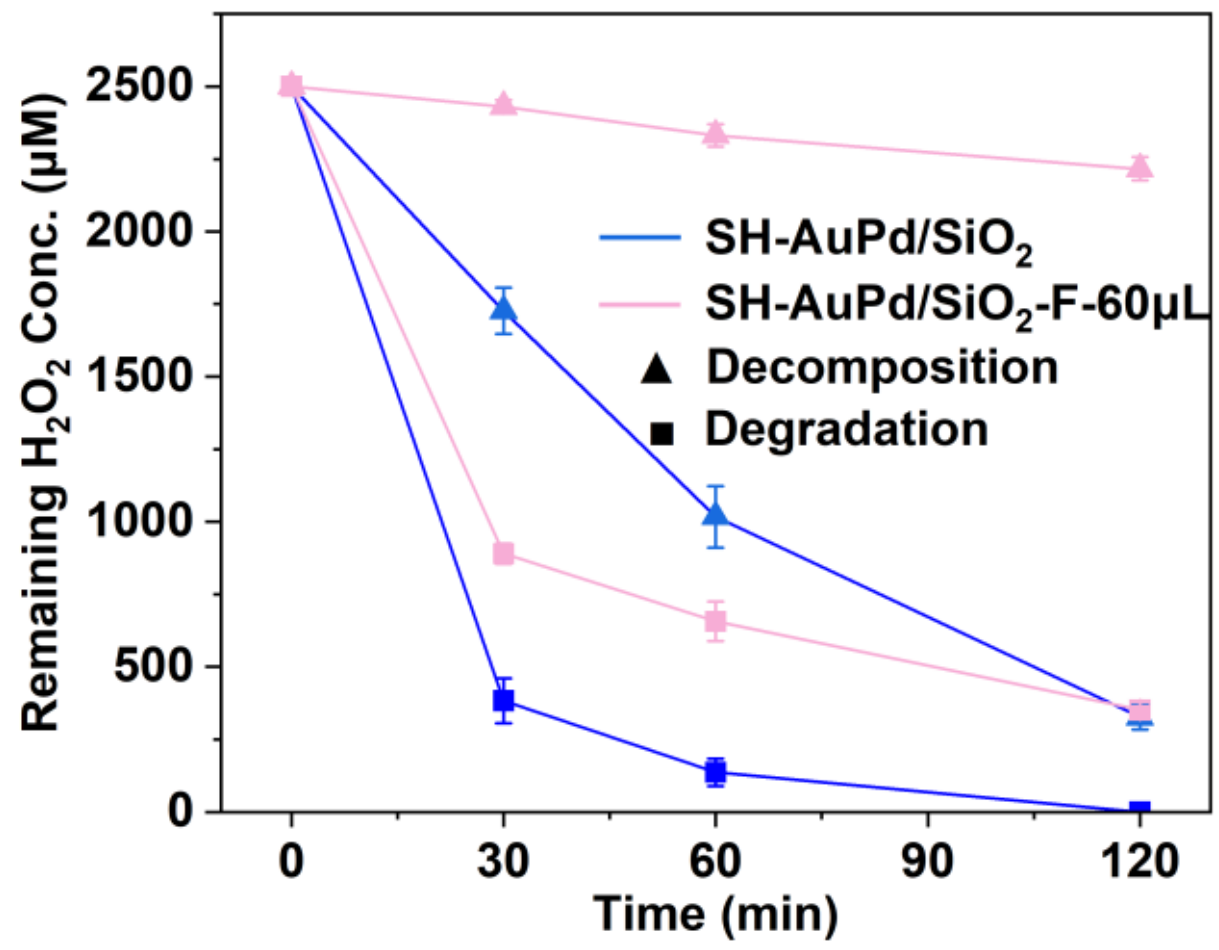

Figure 3e. Time-evolution of H_2O_2 decomposition and degradation (Triangles, decomposition; Squares, degradation). Reaction conditions: 2.5 mg of catalyst, 5 mL of deionized water with 2500 μM H_2O_2, 3 bar air (decomposition) or 3 bar H_2:N_2 = 4% (degradation), 22 °C, 1500 rpm, 30, 60, and 120 min.

5. Figure 4a shows the Kinetic profiles for H_2O_2 synthesis over time, but H_2 conversion and H_2O_2 selectivity data of SH-AuPd/SiO$_2$ for the first ten minutes are missing.

We thank the reviewer for raising this point. During the first 10 min, the H_2 conversion was extremely low with relatively large uncertainty. For this reason, these data were not included in the original figure. To clarify this point, we have now added the early-stage data along with error bars in the revised **Figure 4a**.

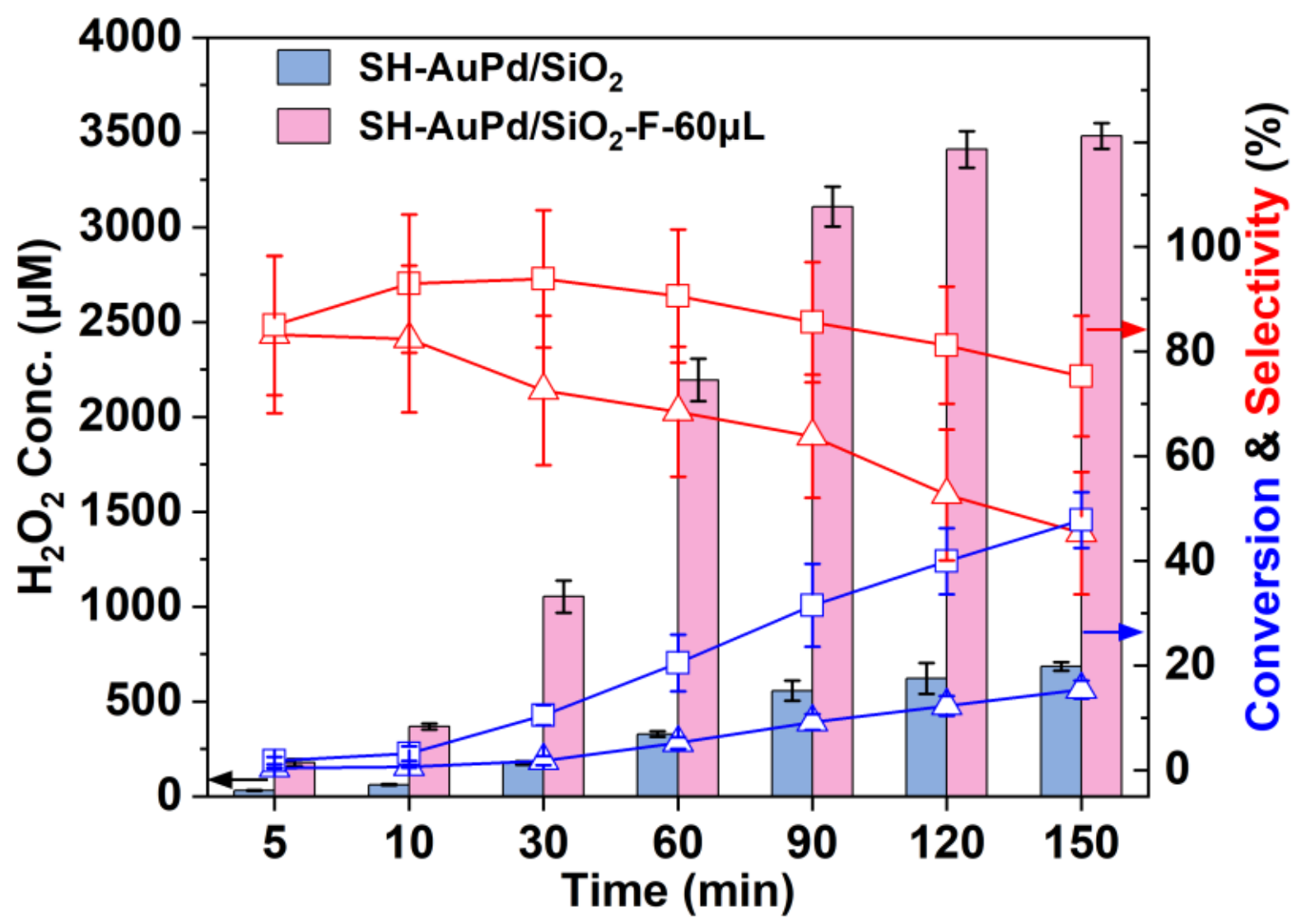

Figure 4. Kinetic profiles for H_2O_2 direct synthesis for SH-AuPd/SiO$_2$ and SH-AuPd/SiO$_2$-F-60μL particles. Conversion and selectivity (triangle, SH-AuPd/SiO$_2$; squares, SH-AuPd/SiO$_2$-F-60μL). Reaction conditions: 2.5 mg of catalyst, 5 mL of deionized water, 3 bar H_2:Air = 4%, 22 °C, 1500 rpm, variable times (5, 10, 30, 60, 90, 120, 150 min).

6. Potential leaching of other elements (e.g., Si from support, F from PFOTES) and their impact on the activity/selectivity should be supplemented, such as the promoting effect of leaked halides.

Accurate quantification of silicon by ICP techniques is inherently challenging due to ubiquitous background contamination, as silicon is widely present in laboratory environments, as well as significant spectral and matrix interferences. Therefore, silicon was not quantified by ICP in this study. Since silicon is covalently bonded to the organic chains, potential Si leaching would be accompanied by the detachment of these chains.

To evaluate this possibility, post-reaction solutions were analyzed by ^{1}H NMR spectroscopy. The results indicate that no fluorinated chain leaching was detected. The corresponding spectra are provided in **Supplementary Figure 9j**.

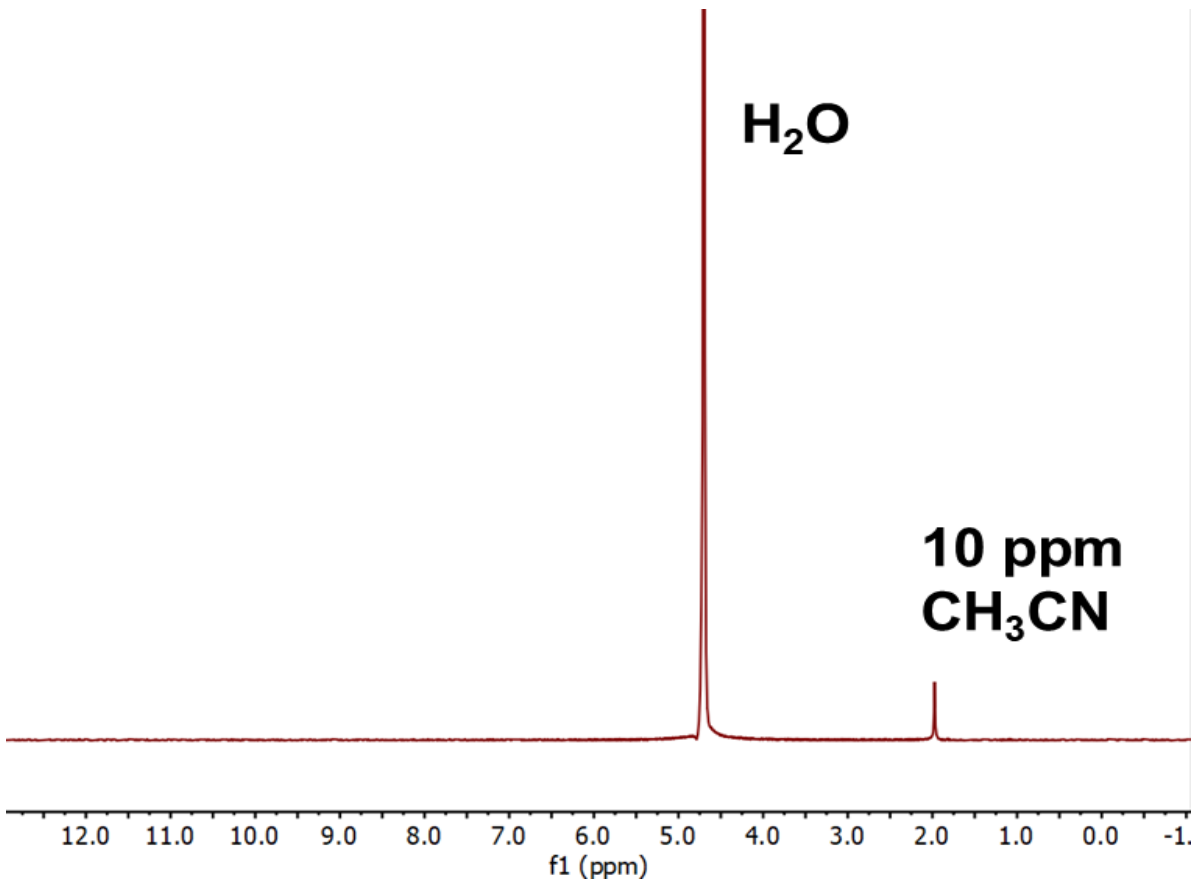

Supplementary Figure 9j. ^{1}H NMR spectrum of the filtrate after solution. Experimental conditions: 10 ppm CH_3CN was used as the internal standard.

7. The author needs to reorganize the layout, format and sequence of the images throughout the entire article. The current version of the readability of the images and writing require further refinement.

We thank the reviewer for this valuable comment. We have carefully reorganized the layout and sequence of images throughout the manuscript to improve clarity and logical flow. In particular, **Figure 1e and Figure 1f** have been switched, and **Supplementary Figures 9 and 11** have been reorganized. However, to group results obtained from the same characterization techniques within the same composite figure for better clarity and comparison, the order of some panels does not strictly follow the sequence in which they are cited in the main text. The formatting has also been refined and the overall readability of both the images and text has been improved. The revised figures and corresponding sections have been updated accordingly in the manuscript.

8. Minor. The reaction conditions of Supplementary Figure 7a were repeated.

Noted and corrected. We added the 'for H_2O_2 production' before 'reaction conditions' to distinguish the 'Reaction conditions for H_2O_2 degradation'.
